# Rotenone causes mitochondrial dysfunction and prevents maturation in porcine oocytes

**DOI:** 10.1371/journal.pone.0277477

**Published:** 2022-11-28

**Authors:** Geun Heo, Ming-Hong Sun, Wen-Jie Jiang, Xiao-Han Li, Song-Hee Lee, Jing Guo, Dongjie Zhou, Xiang-Shun Cui

**Affiliations:** 1 Department of Animal Science, Chungbuk National University, Cheongju, Chungbuk, Republic of Korea; 2 Joint Laboratory of the Modern Agricultural Technology International Cooperation, Ministry of Education, Jilin Agricultural University, Jilin, Changchun, 130118, China; University College London, UNITED KINGDOM

## Abstract

Rotenone is a commonly used insecticidal chemical in agriculture and it is an inhibitor of mitochondrial complex Ⅰ. Previous studies have found that rotenone induces the production of reactive oxygen species (ROS) by inhibiting electron transport in the mitochondria of somatic and germ cells. However, there is little precise information on the effects of rotenone exposure in porcine oocytes during in vitro maturation, and the mechanisms underlying these effects have not been determined. The Cumulus-oocyte complexes were supplemented with different concentrations of rotenone to elucidate the effects of rotenone exposure on the meiotic maturation of porcine oocytes during in vitro maturation for about 48 hours. First, we found that the maturation rate and expansion of cumulus cells were significantly reduced in the 3 and 5 μM rotenone-treated groups. Subsequently, the concentration of rotenone was determined to be 3 μM. Also, immunofluorescence, western blotting, and image quantification analyses were performed to test the rotenone exposure on the meiotic maturation, total and mitochondrial ROS, mitochondrial function and biogenesis, mitophagy and apoptosis in porcine oocytes. Further experiments showed that rotenone treatment induced mitochondrial dysfunction and failure of mitochondrial biogenesis by repressing the level of SIRT1 during in vitro maturation of porcine oocytes. In addition, rotenone treatment reduced the ratio of active mitochondria to total mitochondria, increased ROS production, and decreased ATP production. The levels of LC3 and active-caspase 3 were significantly increased by rotenone treatment, indicating that mitochondrial dysfunction induced by rotenone increased mitophagy but eventually led to apoptosis. Collectively, these results suggest that rotenone interferes with porcine oocyte maturation by inhibiting mitochondrial function.

## Introduction

In mammals, the maturation of oocytes implies preparing them for functions of oocyte development at later stages such as zygote formation and embryogenesis [1]. The complex mechanisms that occur during oocyte maturation include nuclear and cytoplasmic maturation. Although these are distinct processes, nuclear and cytoplasmic maturation are interconnected events that occur simultaneously at a predetermined time [2]. Mitochondria play an important role in these two processes because they are the major organelles that supply adenosine triphosphate (ATP) during oocyte maturation [3]. In addition, the oocyte relies on its mitochondria not only during maturation and for ATP production but also for the maintenance of cellular homeostasis. Thus, the number and activity levels of mitochondria in an oocyte is appropriately regulated for oocyte maturation, fertilization, and embryonic development [4,5]. While mitochondria synthesize energy, O_2_ is reduced to generate superoxide, which is a reactive oxygen species (ROS) [6]. ROS are highly reactive molecules that are derivatives of molecular oxygen species. They are highly reactive and their destructive effects on cellular components can compromise the proper function of organisms [7]. Nevertheless, cells withstand this threat through various antioxidant defense systems [8]. However, failure of the antioxidant defense system can lead to excessive cellular ROS levels that cause oxidative stress, which can damage lipids, nucleic acids, proteins, membranes, and organelles, such as mitochondria. Low to moderate doses of ROS are essential for the regulation of normal physiological functions, as well as the regulation of the immune system and redox balance [9].

Rotenone is a naturally occurring substance derived from the roots, seeds, and stems of *Lonchocarpus* and *Derris* spp.. It is widely used as a pesticide and piscicide, but abuse of rotenone causes environmental pollution and neurotoxicity in livestock and humans [10]. Exposure to rotenone induces the formation of ROS because it inhibits electron transfer from iron-sulfur centers to ubiquinone in the mitochondrial respiratory chain complex Ⅰ[11,12]. In China, foods such as cabbage have a permissible level of less than 0.5 mg/kg of rotenone. However, since the standard has not been established in feed, livestock can ingest rotenone through the feed. In addition, rotenone has been mainly used as an inhibitor of mitochondrial complex Ⅰ in previous experiments[13,14].

As explained, rotenone-induced ROS production may affect mammalian oocytes during maturation and although there are a few studies on the effects of rotenone-induced ROS production on embryos, the effects on in vitro maturation (IVM) of porcine oocytes have not been investigated. Due to IVM being a pre-step for preparing for IVF, and the quality of oocytes during IVF is very important, bad quality oocytes induced by rotenone may cause the failure of post-IVM techniques [15,16].

Therefore, we hypothesized that rotenone exposure influences the induction of mitochondrial dysfunction via ROS production in porcine cumulus-oocyte complexes (COCs) during IVM. We aimed to investigate the relationship between mitochondrial dysfunction and poor oocyte quality following rotenone exposure, to illustrate the issue of rotenone-induced mitochondrial dysfunction in the ROS production response due to rotenone exposure during porcine oocyte maturation.

## Materials and methods

### Collection and in vitro maturation of porcine oocytes

**Fig 1:**
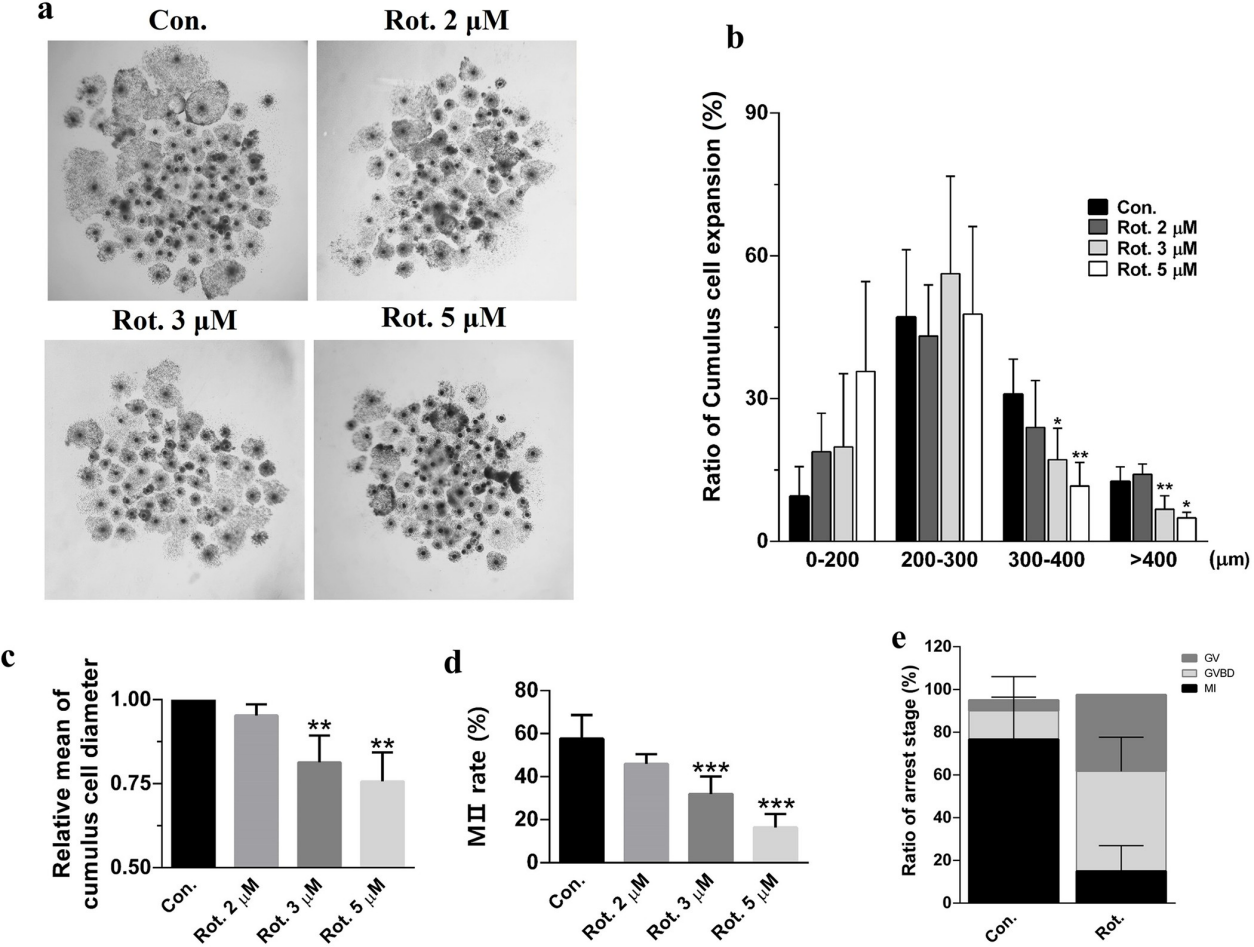
Rotenone reduces the maturation capability of porcine oocytes. **(A)** COC morphology after incubation for 44 h. Scale bar represents 100 μm. **(B)** The ratio of cumulus cells expansion (%) according to grade (μm); **(C)** Relative mean COC diameter; **(D)** Maturation rate (%) in Con. (n = 5,052), Rot 2μM (n = 257), Rot 3μM (n = 6,029), Rot 5μM group (n = 2,845), respectively. **(E)** The ratio of arrest stage in Con. (n = 57) and rotenone Rot 3μM (n = 120). Con., control group. Rot., rotenone-exposed group. *(P < 0.05), **(P < 0.01), ***(P < 0.001) vs control group.

Ovaries from pre-mature pigs were obtained from local slaughterhouses (Farm Story Dodarm B&F, Umsung, Chungbuk, South Korea) and transferred to a laboratory at 38.5°C in saline supplemented with 75 mg/mL penicillin G and 50 mg/mL streptomycin sulfate. Follicles 3–6 mm in diameter were aspirated using an 18-gauge needle attached to a 10 mL disposable syringe. Cumulus-oocyte complexes were selected based on the presence of at least three layers of cumulus cells and a uniformly granulated follicle. After rinsing three times with in-vitro maturation medium TCM-199 (11150‐059; Gibco, Grand Island, NY, USA) supplemented with 0.1 g/L sodium pyruvate, 10 ng/mL epidermal growth factor, 10% (v/v) porcine follicular fluid, 10 IU/mL luteinizing hormone, and 10 IU/mL follicle-stimulating hormone, approximately 70 cumulus-oocyte complexes were each transferred to one well of a 4-well dish (SPL Life Sciences, Seoul, South Korea) containing 500 μL of maturation medium. The medium was covered with mineral oil and the dish was incubated at 38.5°C in a humidified atmosphere containing 5% CO_2_ for 44 h. During incubation, when the experiment was carried out by adding 2, 3, and 5 μM of rotenone, it showed a significant difference from the control group in CC expansion at 3 μM of rotenone. In addition, the ratio of oocytes polar body formation, which means maturation rate, of the 3 μM rotenone-treated group was about half that compared with the control group, so 3 μM was used in the experiment. After measuring CC expansion, the CCs were removed by repeated pipetting in 1 mg/mL hyaluronidase, and only mature oocytes with a polar body were selected. The maturation rate was significantly reduced in the 3 and 5 μM rotenone-treated groups (P < 0.001, Fig 1D).

### Assessment of cumulus cell expansion

After 44 h of IVM, CC expansion was measured using four reference grades. Considering the average CC expansion size in the control group, grades were divided into 0–200, 200–300, 300–400, and > 400 μm. Both the control and treatment groups were separated according to these criteria and the ratios were compared [17].

### ROS measurements

Total ROS levels in mature oocytes were measured using 2’,7’-dichlorodihydrofluorescein diacetate (H2DCF-DA, Cat # D399, Molecular Probes, Eugene, OR, USA) [18]. Oocytes were incubated in phosphate-buffered saline (PBS)/PVA containing 10 μM H2DCF-DA at 38.5°C for 30 min. After incubation, the oocytes were washed three times with PBS/PVA. Fluorescence signals were captured as.jpg files using a digital camera (DP72; Olympus, Tokyo, Japan) connected to a fluorescence microscope (IX70; Olympus). Mitochondrial ROS generation was assessed using the MitoSOX™ mitochondrial peroxide indicator (Thermo Fisher). The oocytes were treated with 200 μM of H_2_O_2_ (Cas: 7722-84-1, Sigma) at 37°C for 15 minutes as a positive control of MitoSOX [19]. Oocytes were incubated in PZM-5 with 10 μM MitoSOX™ solutions at 38.5°C for 30 min. After incubation, oocytes were washed three times with PBS/PVA and fixed in 3.7% paraformaldehyde for 30 min at room temperature (20–25°C). Total and mitochondrial-derived ROS levels were quantified by analyzing the fluorescence intensity of oocytes using Fiji software (National Institutes of Health).

### GSH measurements

To measure GSH levels, oocytes were incubated in PBS/PVA containing 10 μM 4-chloromethyl-6,8-difluoro-7-hydroxycoumarin dye (CellTracker™ Blue CMF2HC Dye, Cat #C12881, Thermo Fisher Scientific, Waltham, USA) or ThiolTracker™ Violet (Cat #T10095, Thermo Fisher Scientific, Waltham, USA) at 38.5°C for 30 min and then washed three times with PBS/PVA. Fluorescence signals were captured as.jpg files using a digital camera (DP72; Olympus, Tokyo, Japan) connected to a fluorescence microscope (IX70, Olympus). GSH levels were quantified by analyzing the fluorescence intensity of oocytes using Fiji software (National Institutes of Health).

### ATP contents assay

ATP contents were measured using a luciferin-luciferase ATP assay system with a luminometer (CentroPRO LB 962), according to the manufacturer’s instructions (A22066, Molecular Probes). Briefly, ten oocytes were collected in a 0.2 mL centrifuge tube containing 20 μL of lysis buffer (20 mM Tris, 0.9% Nonidet-40, and 0.9% Tween 20) and homogenized in a vortex mixer until they were completely lysed. The standard reaction solution was prepared according to the manufacturer’s instructions and placed on ice in the dark before use. Before measurement, the samples (5 μL) were added to 96-well plates and equilibrated for 10 s. Subsequently, 200-μL standard reaction solution was added to each well, and the light signal was integrated for 10 s after a 2 s delay. The light intensity in the control group was arbitrarily set to 1, and the light intensity in the treatment group was measured and expressed as relative values to the control group.

### Immunofluorescence and confocal microscopy

After washing thrice with PBS/PVA, the oocytes were fixed in 3.7% formaldehyde solution at room temperature for 30 min, permeabilized with PBS/PVA containing 0.5% Triton X‐100 at room temperature for 30 min and incubated in PBS/PVA containing 1.0% BSA at room temperature for 1 h. These oocytes were then incubated overnight at 4°C with anti‐TOM20 (Cat #sc-17764, Santa Cruz), anti‐SIRT1 (Cat #60303-1-Ig, Proteintech), anti-ubiquitin (Cat #ab19247, Abcam), anti‐LC3 (Cat #2775S, Cell Signaling Technology), anti‐PINK1 (Cat #ab23707, Abcam), anti-cytochrome C (Cat #ab110325, Abcam), or anti‐Caspase3 (Cat #C8487, Sigma-Aldrich) diluted in blocking solution. The oocytes were treated with 2mM of DTT (Dithiothreitol, Cat #1610611, Bio-rad) at 37°C for 2 hours as a positive control of caspase3 [20]. After washing three times with PBS/PVA, the oocytes were incubated at room temperature for 1 h with Alexa Fluor 488™ donkey anti-mouse IgG (H + L) or Alexa Fluor 546™ donkey anti-rabbit IgG (H + L). To test the antibody specificity of the immunofluorescence method, only the secondary antibody was stained in mature oocytes as a negative control. The oocytes were then stained with 10 μg/mL Hoechst 33342 for 10 min, washed thrice with PBS/PVA, mounted onto slides, and examined under a confocal microscope (Zeiss LSM 710 META). The images were processed using Zen software (version 8.0, Zeiss).

To detect total and active mitochondria, oocytes were first incubated with 500 nM MitoTracker Red CMXRos (Cat #M7512, Invitrogen) at 38.5°C for 30 min. After three washes with PBS/PVA, immunofluorescence staining for TOM20 was performed as described above. Total and active mitochondria were quantified by analyzing the fluorescence intensity of the oocytes using Fiji software (National Institutes of Health, Bethesda, MD, USA).

### Western blot analysis

As previous describe [21], briefly a total of 100 porcine oocytes per group were lysed with 1 × sodium dodecyl sulfate sample buffer by heating at 98°C for 10 min. The proteins were separated by sodium dodecyl sulfate-polyacrylamide gel electrophoresis and transferred onto polyvinylidene fluoride membranes. Next, the membranes were blocked in 5% (w/v) skim milk for 1 h and then incubated at 4°C overnight with anti-SIRT1 (Cat #60303-1-Ig, Proteintech), anti-ubiquitin (Cat #ab19247, Abcam), or GAPDH (Cat #sc-365062, Santa Cruz), followed by incubation at room temperature for 1 h with horseradish peroxidase-conjugated goat anti-mouse IgG or goat anti-rabbit IgG (1:20000; Santa Cruz Biotechnology). The membrane was detected using Pierce ECL substrate (Thermo Fisher Scientific). Blots were visualized using a CCD camera and UVISoft software (UVITEC, Cambridge, UK).

### mtDNA copy number measurement

Each pool of three oocytes was transferred to a 0.2 mL tube containing 8 μL lysis buffer (20 mM Tris, 0.4 mg/mL proteinase K, 0.9% Nonidet-40, and 0.9% Tween 20) at 65°C for 30 min, followed by 95°C for 5 min. The samples were diluted 1:25 in sterile ddH_2_O before analysis. Real-time PCR was performed using the WizPure™ qPCR Master (Super Green) Mix (Cat # W1731-8, Wizbiosolution). Amplification was conducted as follows: 95°C for 3 min, followed by 40 cycles at 95°C for 15 s, 60°C for 25 s, and 72°C for 10 s, with a final extension at 72°C for 5 min. The real-time PCR primer set was: ND1-F (5′- CCT ACT GGC CGT AGC ATT CC-3′), ND1-R (5′-GAG GAT GTG CCT GGT CGT AG -3′). The mitochondrial DNA copy number was analyzed using the 2^−ΔΔCT^ method [22].

### Evaluation of co-localization

Oocytes were stained with Mito-Tracker, cytochrome C, PINK1, Ubiquitin, LC3 or TOM20 according to the method described in ’Immunofluorescence and confocal microscopy’. The oocytes were observed using a laser scanning confocal microscope (Zeiss LSM 710 META, Germany). Co-localization of mitochondrial/cytochrome C, PINK1/TOM20 and LC3/TOM20 was evaluated using Pearson’s correlation coefficient. The overlaps of the Ubiquitin/TOM20 and LC3/TOM20 were visualized using the image calculator function of Fiji software (National Institutes of Health, Bethesda, MD, USA).

### Statistical analysis

**Fig 2:**
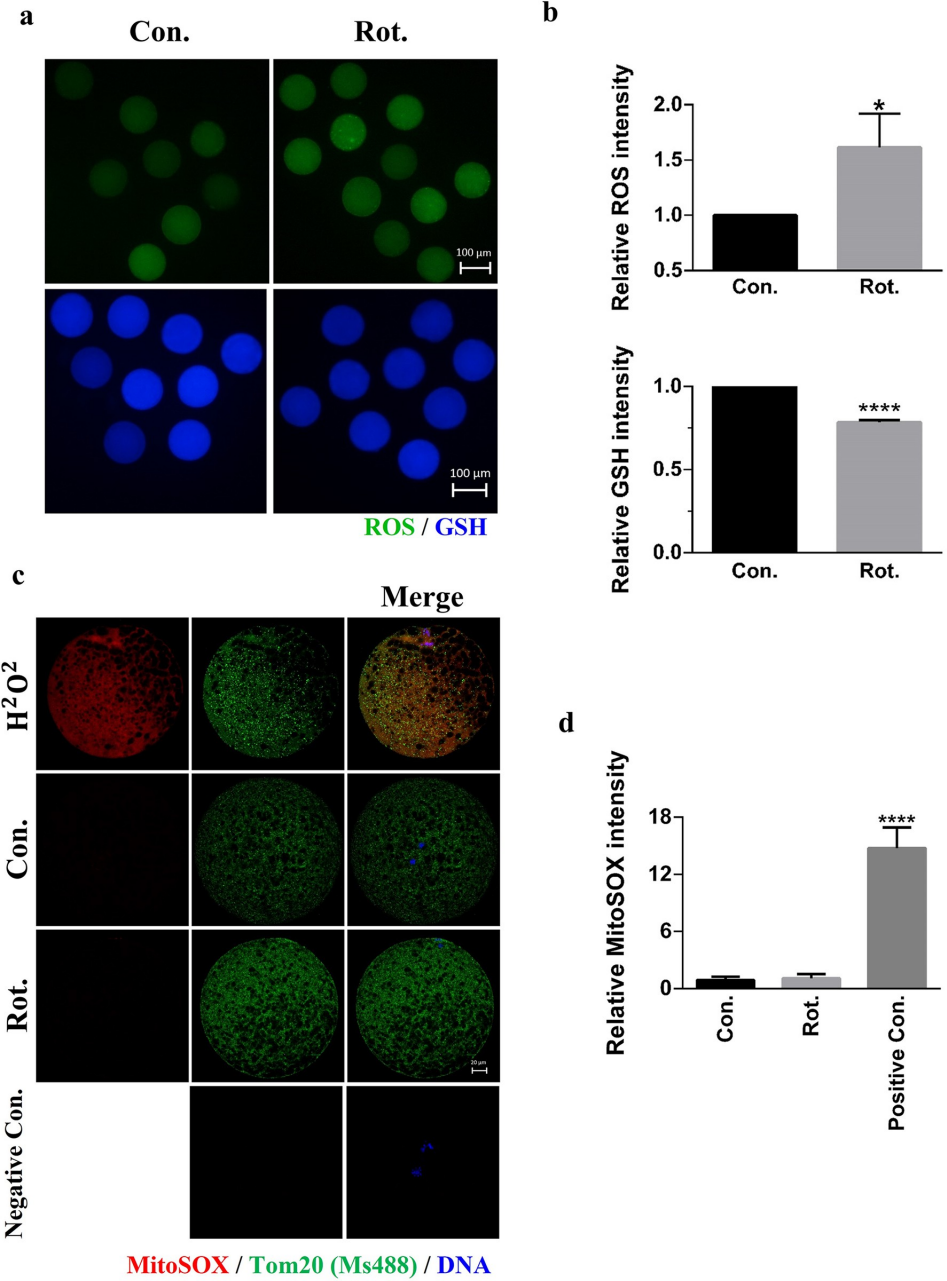
Rotenone induces oxidative stress in porcine oocytes. **(A and B)** Total ROS and total GSH levels in Con. and Rot. Group (n = 30). Scale bars represent 100 μm. Green = ROS, blue = GSH. **(C)** Immunofluorescence images of MitoSOX and TOM20. Red = MitoSOX, green = TOM20, blue = DNA. **(D)** The relative intensity of MitoSOX expression of oocytes in the Con., Rot. and positive control (H_2_O_2_) of MitoSOX Group (n = 15). The oocytes were treated with 200 μM of H_2_O_2_ as a positive control of MitoSOX. Scale bar represents 20 μm. Con., control group. Rot., 3μM rotenone-exposed group. Negative Con., only the secondary antibody was stained in mature oocytes as a negative control. *(P < 0.05), ****(P < 0.0001).

**Fig 3:**
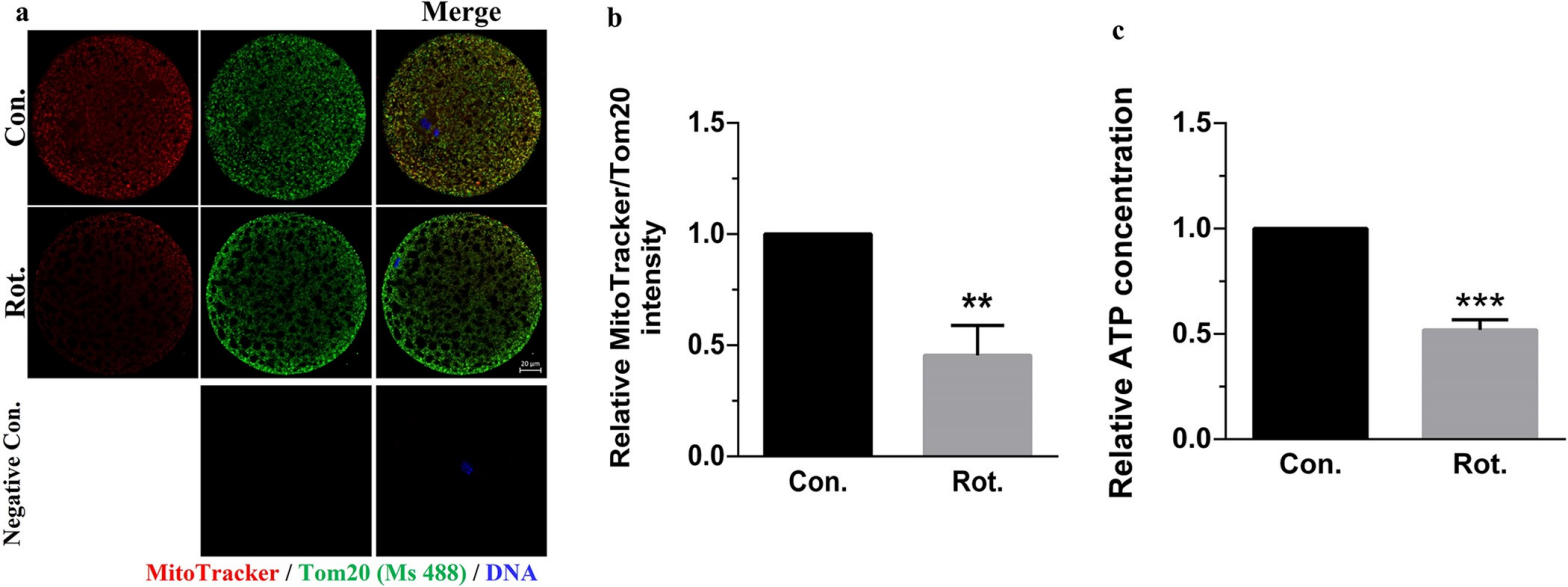
Rotenone induces mitochondrial dysfunction in porcine oocytes. **(A)** Relative fluorescence intensity of Mito-Tracker and TOM20 in porcine oocytes after rotenone treatment. Red = Mito-Tracker, green = TOM20, blue = DNA. Scale bars represent 20 μm. **(B)** Ratio of Mito-Tracker/TOM20, representing the number of functional mitochondria and total mitochondria, respectively (n = 30). **(C)** Relative ATP production in control and rotenone-treated oocytes (n = 16). Con., control group. Rot., 3μM rotenone-exposed group. Negative Con., only the secondary antibody was stained in mature oocytes as a negative control. **(P < 0.01), ***(P < 0.001).

**Fig 4:**
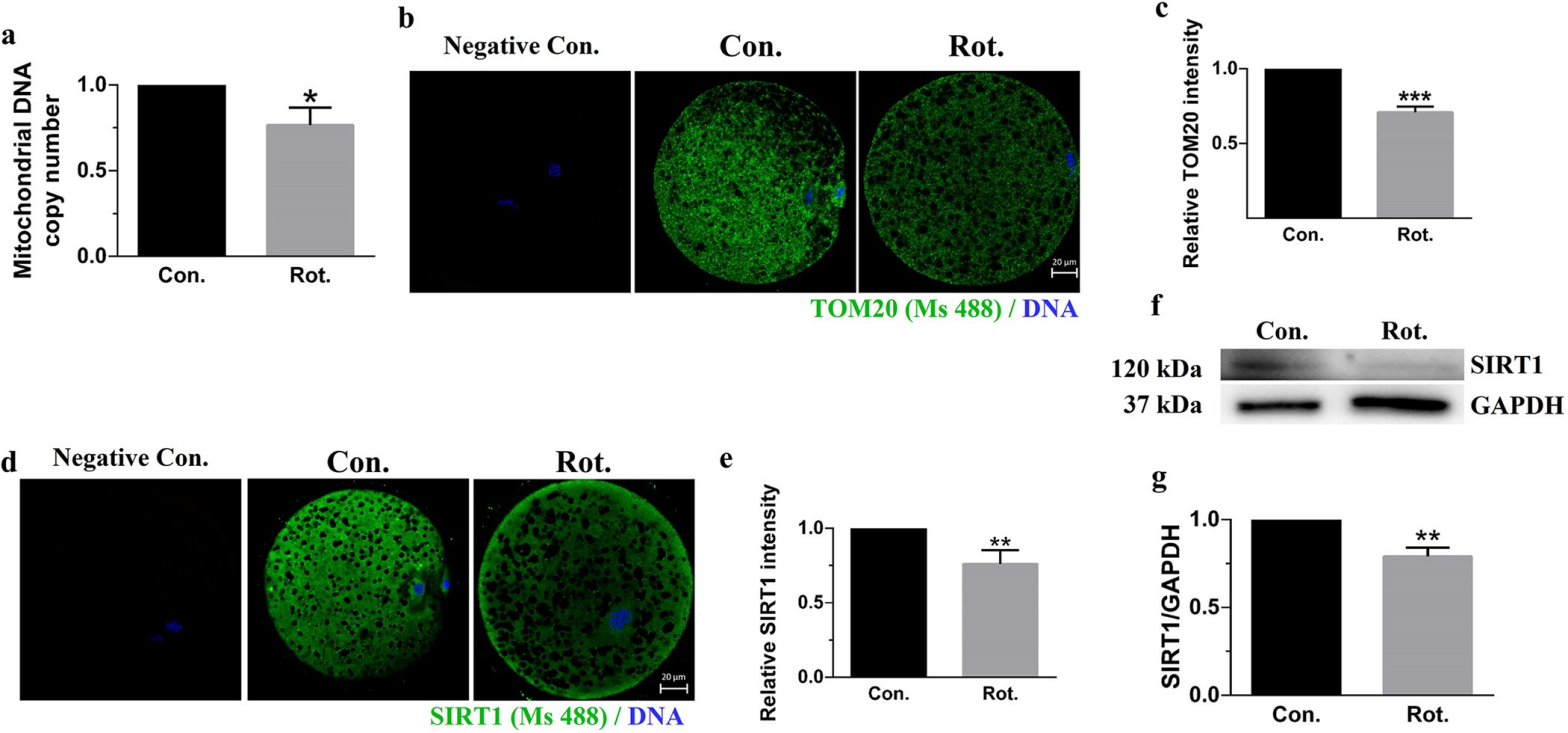
Rotenone disrupts mitochondrial biogenesis in porcine oocytes. **(A)** Relative mitochondrial DNA copy number of oocytes in the control and rotenone-treated groups (n = 3). **(B)** Representative images of TOM20 intensity in the control and rotenone-treated groups. Green = TOM20, blue = DNA. Scale bar represents 20 μm. **(C)** Relative fluorescence intensity of TOM20 in the control and rotenone-treated groups (n = 20) **(D)** Representative images of SIRT1 intensity in the control and rotenone-treated groups. Green = SIRT1, blue = DNA. Scale bar represents 20 μm. **(E)** Relative fluorescence intensity of SIRT1 in the control and rotenone-treated groups (n = 30). **(F)** Western blot of SIRT1 (120 kDa) protein expression in porcine oocytes after rotenone treatment. **(G)** Relative intensity analysis for SIRT1 (n = 3). Con., control group. Rot., 3μM rotenone-exposed group. Negative Con., only the secondary antibody was stained in mature oocytes as a negative control. *(P < 0.05), **(P < 0.01), ***(P < 0.001).

**Fig 5:**
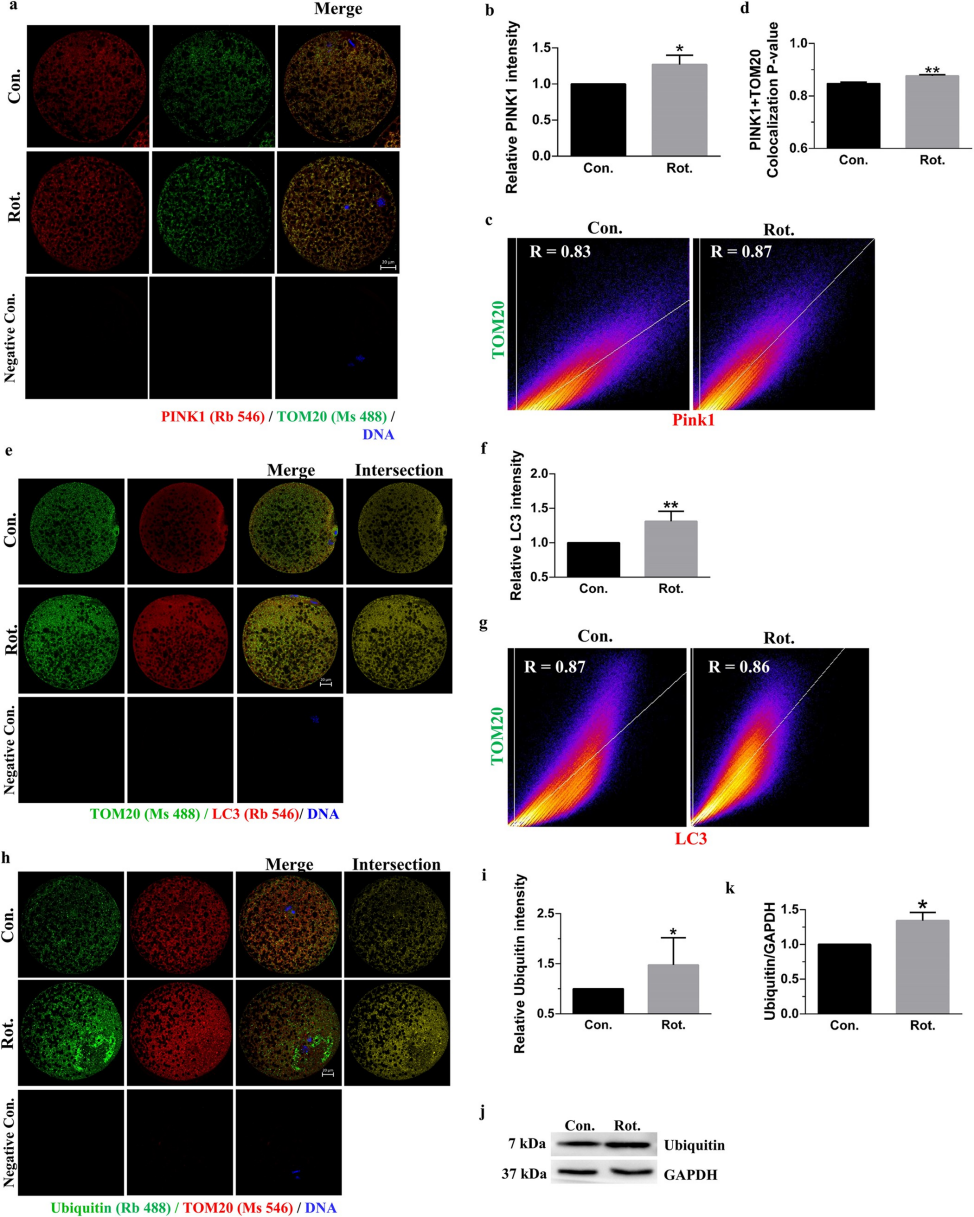
Rotenone induces mitophagy in porcine oocytes. **(A)** Fluorescence images of PINK1 and TOM20 in porcine oocytes after rotenone treatment. Red = PINK1, green = TOM20, blue = DNA. Scale bars represent 20 μm. **(B)** Relative fluorescence intensity of PINK1 in the control and rotenone-treated groups (n = 30). **(C)** Colocalization graphs of PINK1 and TOM20 in the control and rotenone-treated groups. x-axis: PINK1; y-axis: TOM20. **(D)** Pearson value of colocalization of PINK1 and TOM20 in the control and rotenone-treated groups, which could indicate damaged mitochondria (n = 35). **(E)** Images of LC3 fluorescence in porcine oocytes of the control and rotenone-treated groups. Green = TOM20, red = LC3, blue = DNA, yellow = intersection of green and red. Scale bars represents 20 μm. **(F)** Relative fluorescence intensity of LC3 on the intersection of green and red in the control and rotenone-treated groups (n = 28). **(G)** Colocalization graphs of LC3 and TOM20 in the control and rotenone-treated groups. x-axis: LC3; y-axis: TOM20. **(H)** Images of ubiquitin fluorescence in porcine oocytes of the control and rotenone-treated groups. Green = ubiquitin, red = TOM20, blue = DNA, yellow = intersection of green and red. Scale bars represents 20 μm. **(I)** Relative fluorescence intensity of ubiquitin on the intersection of green and red in the control and rotenone-treated groups (n = 24). **(J)** Western blot of monomeric ubiquitin (7 kDa) expression in porcine oocytes after rotenone treatment. **(K)** Band intensity analysis for ubiquitin (n = 3). Con., control group. Rot., 3μM rotenone-exposed group. Negative Con., only the secondary antibody was stained in mature oocytes as a negative control. *(P < 0.05), **(P < 0.01).

**Fig 6:**
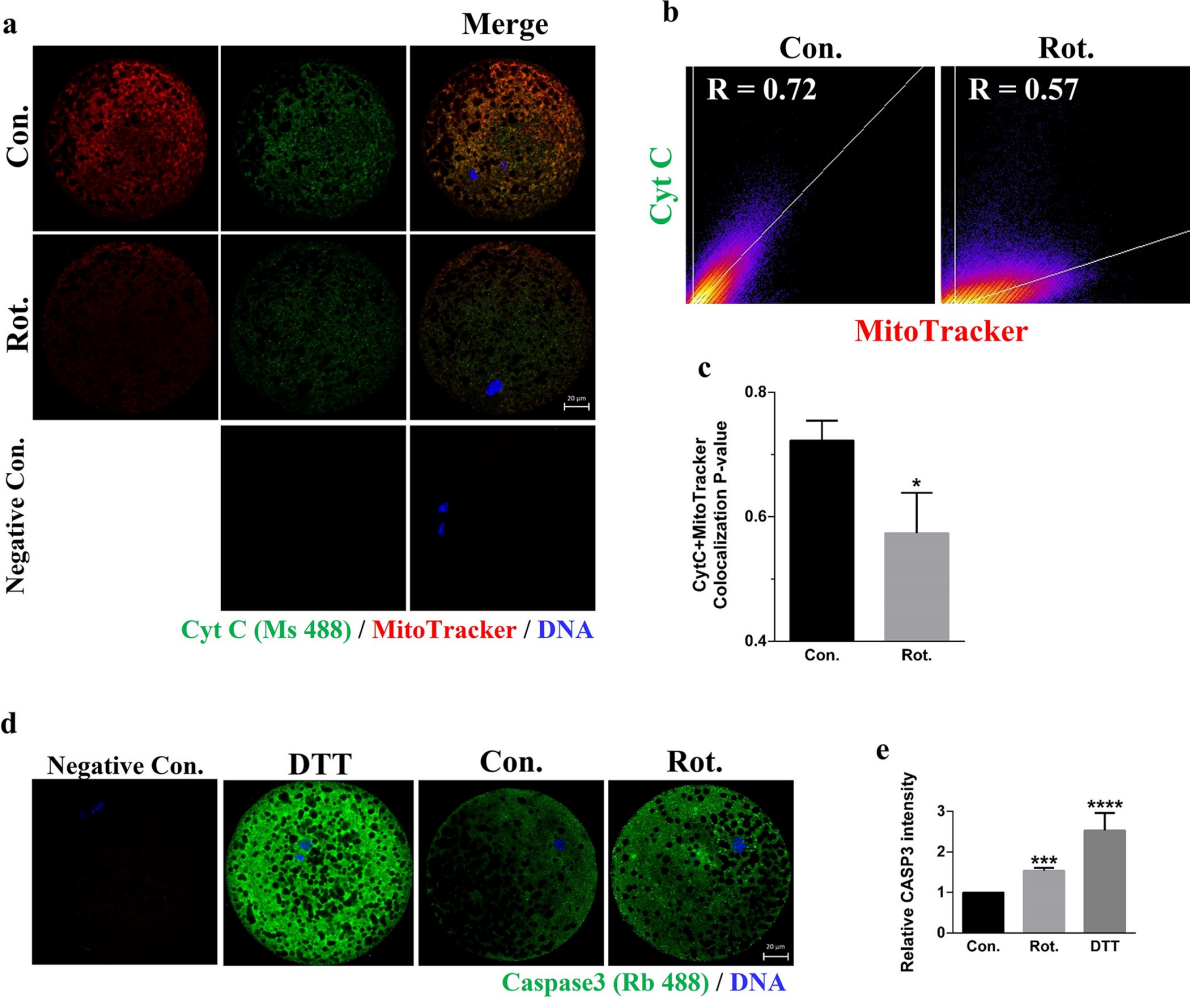
Rotenone leads to apoptosis in porcine oocytes. **(A)** Fluorescence images of cytochrome C and Mito-Tracker in porcine oocytes after rotenone treatment. Green = cytochrome C, red = Mito-Tracker, blue = DNA. Scale bars represent 20 μm. **(B)** Colocalization graphs of cytochrome C and Mito-Tracker in the control and rotenone-treated groups. x-axis: MitoTracker; y-axis: TOM20. **(C)** Pearson value of colocalization of cytochrome C and Mito-Tracker in the control and rotenone-treated groups, which could indicate activation of apoptosis (n = 50). **(D)** Representative images of caspase3 intensity in the positive control (DTT), control and rotenone-treated groups. Green = caspase3, blue = DNA. Scale bar represents 20 μm. **(E)** Relative fluorescence intensity of caspase3 in the control and rotenone-treated groups (n = 19). Con., control group. Rot., 3μM rotenone-exposed group. Negative Con., only the secondary antibody was stained in mature oocytes as a negative control. *(P < 0.05), ***(P < 0.001) ****(P < 0.0001).

**Fig 7:**
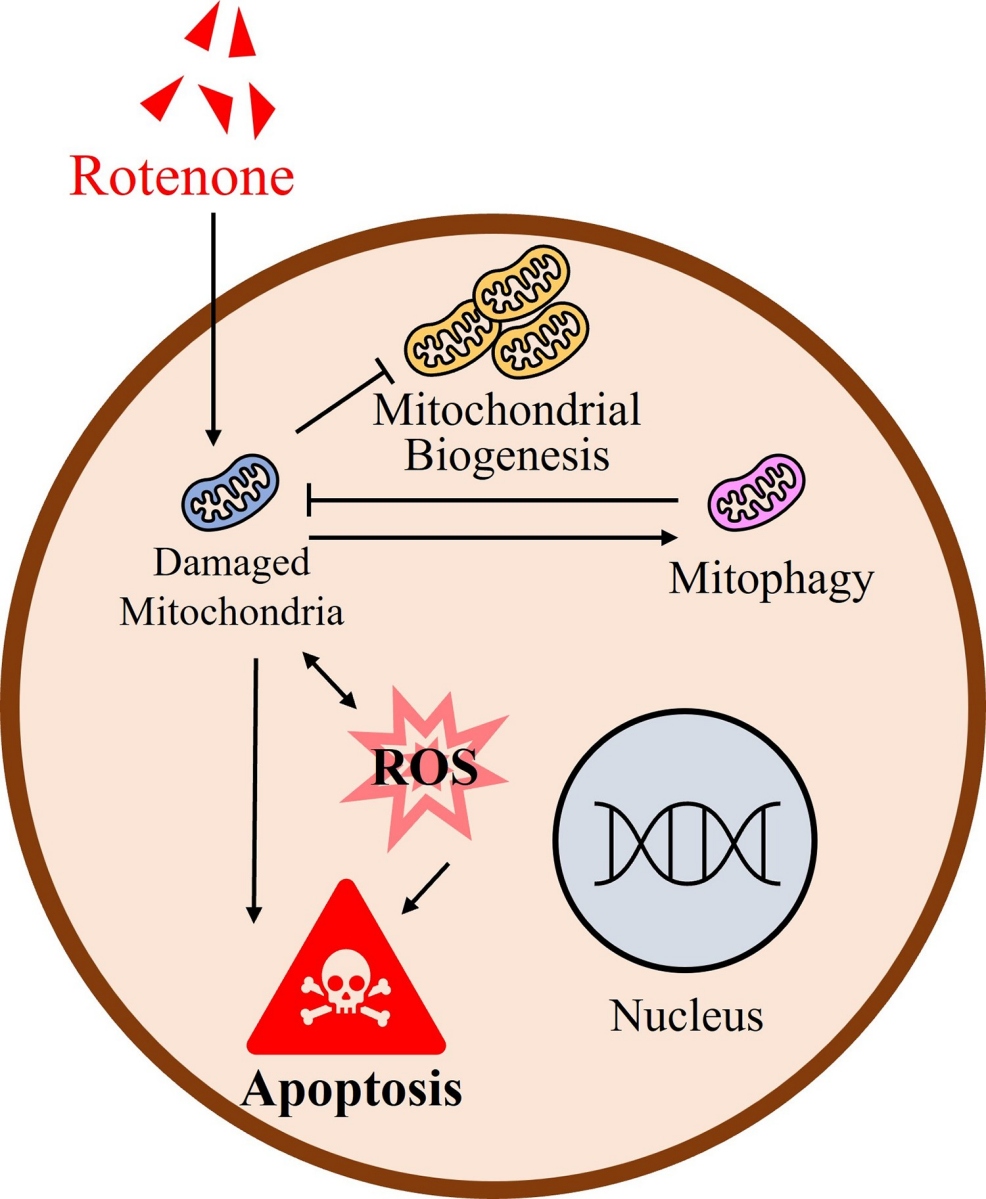
Rotenone exposure disturbs meiotic maturation by inducing mitochondrial dysfunction in porcine oocytes. Rotenone exposure causes mitochondrial dysfunction, and damaged mitochondria inhibit mitochondrial biosynthesis. Damaged mitochondria trigger mitophagy, and mitophagy can mitigate mitochondrial damage. Damaged mitochondria induce ROS, eventually leading to apoptosis.

Each experiment was repeated at least three times, and the representative images are shown in Figs 1–7. All data were analyzed using Student’s t-test. All percentage data were subjected to arcsine transformation before statistical analysis and are presented as the mean ± standard error (SEM). Significance was set at P < 0.05. All calculations were performed using SPSS software v.19 (IBM SPSS, Chicago, IL, USA).

## Results

### Rotenone reduces the maturation capability of porcine oocytes

To determine whether rotenone affects the maturation of porcine oocytes, COCs were cultured in IVM medium supplemented with rotenone (2, 3, and 5 μM) for 44 h (Fig 1A). After 44 hours of IVM, MⅡ stage oocytes were selected for further analysis. Subsequently, cumulus cell (CC) expansion was investigated. The ratio of COCs with diameters larger than 300 μm was reduced after supplementation with 3 and 5 μM rotenone (Fig 1B). In addition, the mean value of CC expansion in the rotenone-treated groups at concentrations of 3 and 5 μM was significantly decreased (P < 0.01; Fig 1C). The total number of COCs in the control group used was n = 938 and the rotenone group was n = 927. Also, the rate of polar body formation, which indicates the maturation of oocytes, was significantly reduced in the rotenone-treated group in which the concentrations of 3uM and 5uM compared to the control group (Fig 1D).

Therefore, rotenone at a concentration of 3 μM was used for further experiments. These results suggest that rotenone affects oocyte metabolism and maturation capacity and that because CC expansion is essential for oocyte maturation [23], rotenone inhibited CC expansion, and thus CCs did not provide metabolic support to the oocytes. The arrest stage of oocytes was investigated, and it was confirmed that the oocytes in the GV and GVBD stages were significantly higher in the rotenone-treated group than in the control group (Fig 1E). In addition, it was confirmed that the antioxidant (melatonin) recovered the oocyte maturation rate induced by rotenone. Furthermore, antioxidants also increased the rate of cleavage after pathogenetic activation but did not recover the blastocyst rate (S1 Fig).

### Rotenone induces oxidative stress in porcine oocytes

We hypothesized that the cause of the decline in the maturation capacity of oocytes was oxidative stress induced by rotenone treatment, and therefore, we measured ROS fluorescence. As shown in Fig 2A and 2B, total ROS was examined in porcine oocytes using the H2DCF-DA reaction to investigate oxidative stress. The relative ROS intensity in the rotenone-treated group was significantly higher than that of the control group (P < 0.05). In addition, the fluorescence of GSH, the opposite of ROS, was decreased in the rotenone-treated group compared with the control group (P < 0.0001, Figs 2A, 2B and S2). Although mitochondrial ROS were detected by MitoSOX (Fig 2C), a mitochondrial peroxide indicator, there was no significant difference between the control and rotenone-treated groups (Fig 2D). As oxidative stress can be caused by increased ROS production [24], this suggests that rotenone treatment causes oxidative stress by producing excess ROS in oocytes.

### Rotenone induces mitochondrial dysfunction in porcine oocytes

To determine whether oxidative stress was mitochondrially induced, we investigated mitochondrial function. In porcine oocytes, the mitochondrial outer membranes were stained with TOM20, and active mitochondria were detected with MitoTracker Red. The ratio of fluorescence intensity (Mito-Tracker/TOM20) was significantly reduced in the rotenone-treated group compared with that of the control group (P < 0.01, Fig 3A and 3B). As expected, relative ATP production was markedly decreased in the rotenone-treated group compared to that of the control group (P < 0.001, Fig 3C). This suggests that rotenone treatment reduces the number of active mitochondria in porcine oocytes, thereby reducing ATP production.

### Rotenone disrupts mitochondrial biogenesis in porcine oocytes

We investigated mitochondrial biosynthesis and mitochondrial DNA copy number to determine whether total mitochondrial number caused decreased ATP production. The mtDNA copy number in rotenone-treated oocytes was lower than that of the control oocytes (P < 0.05, Fig 4A). Furthermore, TOM20 was stained to measure the quantification of mitochondria. The fluorescence of TOM20 was decreased in the rotenone-treated group compared to the control group (Fig 4B and 4C). This suggests that rotenone disturbs mitochondrial biogenesis by inducing mitochondrial dysfunction and deficiency. In addition, SIRT1, which can activate PGC-1α transcription, was investigated (Fig 4D). The fluorescence intensity of SIRT1 in the rotenone group significantly decreased (P < 0.01, Fig 4D and 4E). Additionally, as shown in Fig 4F and 4G, SIRT1 expression, which was reduced in the rotenone-treated group compared with the control group (P < 0.01), was investigated using western blotting.

### Rotenone induces mitophagy in porcine oocytes

We observed mitophagy in oocytes to determine whether the decrease in active and total mitochondrial number was due to the removal of damaged mitochondria. PINK1 accumulates on the outer membrane of damaged mitochondria [25] and can therefore act as a marker of damaged mitochondria [26]. The fluorescence intensity of PINK1 was higher in the rotenone-treated group than in the control group (P < 0.05, Fig 5A and 5B). Colocalization of TOM20, which labels the outer mitochondrial membrane, and PINK1 was also increased in the rotenone-treated group, suggesting that rotenone damages mitochondria (P < 0.01, Fig 5C and 5D).

Ubiquitin activation via the binding of PINK1/Parkin was also investigated. The fluorescence of LC3 and ubiquitin on mitochondria was detected by co-staining with TOM20. As shown in Fig 5E and 5F, the relative intensity of LC3 on the mitochondria was increased after the rotenone treatment (P < 0.01). The colocalization of LC3 and TOM20 was no different in both control and rotenone-treatment groups (Fig 5G), which suggests that the intensity of LC3 correlates with mitochondrial. Likewise, rotenone treatment increased the relative intensity of ubiquitin on mitochondria (P < 0.05; Fig 5H and 5I). Meanwhile, the total protein level was observed by western blotting, which showed that the ubiquitin was increased in the rotenone-treated group (P< 0.05; Fig 5J and 5K). These results indicate that rotenone treatment damaged the mitochondria of porcine oocytes and induced mitophagy.

### Rotenone leads to apoptosis in porcine oocytes

Consequently, we investigated whether rotenone exposure induced apoptosis in porcine oocytes. As shown in Fig 6A–6C, rotenone treatment reduced the colocalization of cytochrome C/Mito-Tracker in comparison with the control (P < 0.05), which indicates that cytochrome C was released from the cristae of mitochondria into the cytoplasm; cytochrome C release can activate apoptosis [27]. Furthermore, activated caspase3 was also examined as an apoptosis marker. As expected, the fluorescence intensity of activated caspase 3 was significantly increased in the rotenone-treated group compared with the control group (P < 0.001; Fig 6D and 6E).

## Discussion

In research, rotenone is commonly used as an inhibitor of the mitochondrial complex Ⅰ. However, most studies on rotenone use somatic cells of Parkinson’s disease, and there are few studies on rotenone in embryos or sperm in mammalian germ cells. There is limited research on using rotenone to reduce the maturation rate of mammalian oocytes, and the exact mechanism has not yet been elucidated. For example, rotenone increases the intensity of NADH and decreases that of FAD in mouse oocytes [28], thereby decreasing the rate of maturation [29]. In addition, research has shown that exposure to rotenone reduces in vitro fertilization rates of bovine oocytes [30]. In pigs, rotenone treatment reduces blastocyst ratio and blastocyst quality [12]. Since the effects of rotenone exposure on porcine oocytes and the underlying mechanisms have not been elucidated, we hypothesized that rotenone induces mitochondrial dysfunction during the maturation of porcine oocytes, thereby reducing their maturation capacity.

During the maturation of porcine oocytes in vitro, rotenone supplementation not only reduced CCs expansion in both groups, but also the average CCs expansion diameter and maturation rate. Previous studies have shown that CCs expansion affects oocyte maturation and quality [31,32], and CCs expansion was used as one of the markers to measure oocyte quality. Thus, it was confirmed that rotenone exposure reduced the maturation capacity of porcine oocytes, and we investigated oxidative stress as the potential cause. Similar to many other studies [33–35], this study showed that ROS was upregulated, and oxidative stress was induced in porcine oocytes as a result of rotenone treatment. In the previous study [36–38], GSH detected using Cell Tracker was the opposite concept of ROS, and the rotenone treated group decreased compared to the control group. As oxidative stress was speculated to be the result of rotenone-mediated suppression of mitochondrial function, mitochondrial function was also investigated. Consistent with a previous study [39], we found that rotenone exposure downregulated active mitochondria in porcine oocytes during in vitro maturation. A decrease in the intensity of Mito-Tracker binding to live mitochondria meant fewer active mitochondria, and we hypothesized that this was responsible for halving ATP production. In addition, rotenone treatment reduced mtDNA copy number in murine PC12 cells and inhibited these effects in porcine oocytes [40]. This evidence that rotenone can inhibit the expression of SIRT1 supports a previous study showing that downregulation of SIRT1 reduces the expression of PGC-1α and mitochondrial biogenesis machinery [41]. This suggests that the cause of the decreased ATP production is not only a decrease in the number of active mitochondria, but also in the total number of mitochondria. Despite the release of mtDNA due to the increase in mitophagy [42], the cause of the decreased mtDNA can be considered to be due to the decrease in SIRT1 [43].

However, this is contrary to previous results [12] showing that the addition of rotenone increased mitochondrial biogenesis (SIRT1) in porcine embryos. This could be explained as part of a mechanism to compensate for rotenone-induced energy shortages because more ATP [44] is required during embryonic development than during oocyte maturation [12].

Similar to previous studies [45,46], our study demonstrates that rotenone treatment resulted in increased mitophagy in porcine oocytes, suggesting that rotenone treatment damaged the mitochondria. Although a recent study suggested that mitophagy may extinguish ATP, we found that mitophagy inhibits apoptosis [47]. We also found that rotenone treatment of porcine oocytes had the opposite effect, upregulating mitophagy and apoptosis concomitantly. In this regard, we suggest rotenone-induced CC deterioration. Rotenone treatment may have inhibited ATP production in oocytes, and the oocytes may not have been able to provide metabolic support for oocyte maturation. As mentioned above, CC expansion of the cumulus-oocyte complex is required for meiotic maturation and acquisition of developmental capacity [23]. Metabolic cooperation between oocytes and cumulus cells plays an important role in oocyte maturation [48], suggesting that their metabolic cooperation is not well achieved. Therefore, we hypothesized that apoptosis also increased for the same reason, despite the increase in oocyte mitophagy. Although the results of this study do not reveal a correlation between mitophagy and apoptosis, they suggest a negative correlation between rotenone treatment and the maturation of porcine oocytes.

In conclusion, rotenone treatment during maturation of porcine oocytes damaged mitochondria. Damaged mitochondria inhibit mitochondrial biosynthesis, induce mitophagy, and cause the production of reactive oxygen species. Eventually, rotenone-treated porcine oocytes induce apoptosis, despite an increase in mitophagy (Fig 7). Therefore, future studies should focus on the correlation between mitophagy, and apoptosis based on the metabolic cooperation between cumulus cells and oocytes.

## Supporting information

S1 Fig Rotenone exposure to oocytes is not recovered during in vitro culture, even if removed after artificial activation. **(A)** Maturation rate (%) in Con. (n = 2,950), Rot 3μM (n = 1,515), Rot. + Mel. group (n = 1,416), respectively. The concentration of melatonin is 1μM. **(B)** Cleavage rate (%) in Con. (n = 440), Rot 3μM (n = 196), Rot. + Mel. group (n = 248), respectively. **(C)** Blastocyst rate (%) in Con. (n = 440), Rot 3μM (n = 196), Rot. + Mel. group (n = 248), respectively. Con., control group. Rot., rotenone-exposed group. Rot. + Mel., Incubated with rotenone 3μM and melatonin 1μM.
(TIF)

S2 Fig Rotenone exposure decreases GSH level in porcine oocytes. **(A)** Representative fluorescence and bright images of GSH intensity in the control and rotenone-treated groups. Blue = 404nm, red = 526nm. (B) Relative fluorescence intensity of GSH in the control (n = 9) and rotenone-treated groups (n = 12). Con., control group. Rot., 3μM rotenone-exposed group. ****(P < 0.0001)
(TIF)

S1 Raw images (PDF)

S2 Raw images (ZIP)

S3 Raw images (ZIP)

S4 Raw images (ZIP)

S5 Raw images (ZIP)

S6 Raw images (ZIP)

S7 Raw images (ZIP)

S8 Raw images (ZIP)

S9 Raw images (ZIP)

S10 Raw images (ZIP)

S11 Raw images (ZIP)

S12 Raw images (ZIP)
