# Supplementary figures and images for: Rotenone causes mitochondrial dysfunction and prevents maturation in porcine oocytes
Source: PLoS One. 2022 Nov 28;17(11):e0277477. doi: 10.1371/journal.pone.0277477 (PMC9704683; doi:10.1371/journal.pone.0277477)

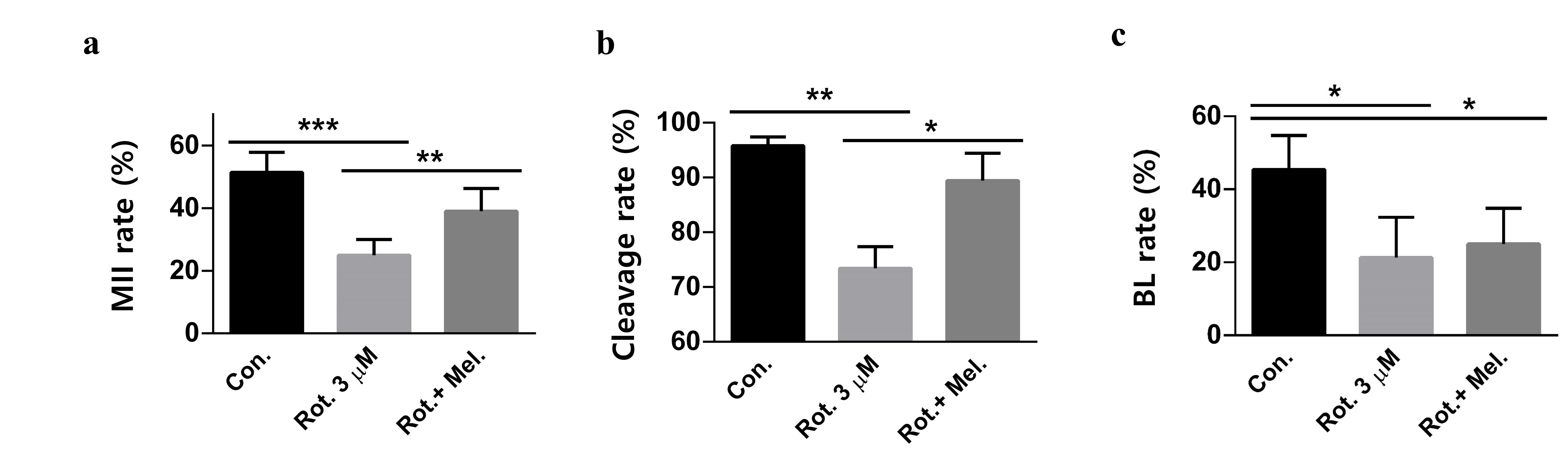

Supplement: S1 Fig — (A) Maturation rate (%) in Con. (n = 2,950), Rot 3μM (n = 1,515), Rot. + Mel. group (n = 1,416), respectively. The concentration of melatonin is 1μM. (B) Cleavage rate (%) in Con. (n = 440), Rot 3μM (n = 196), Rot. + Mel. group (n = 248), respectively. (C) Blastocyst rate (%) in Con. (n = 440), Rot 3μM (n = 196), Rot. + Mel. group (n = 248), respectively. Con., control group. Rot., rotenone-exposed group. Rot. + Mel., Incubated with rotenone 3μM and melatonin 1μM. (TIF) [file pone.0277477.s001.tif]

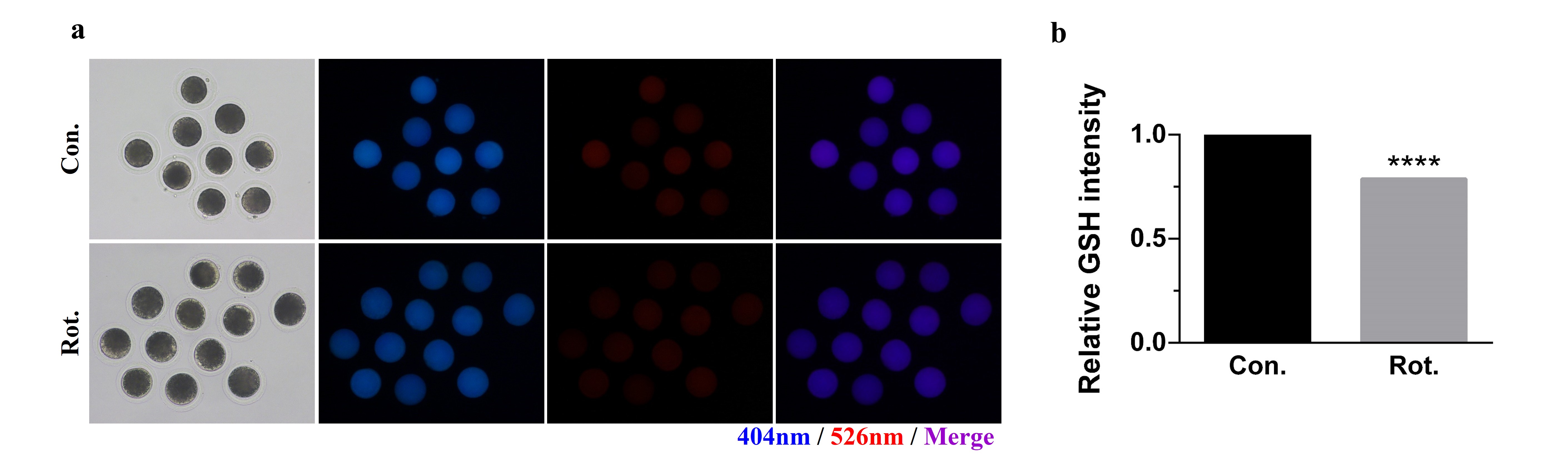

Supplement: S2 Fig — (A) Representative fluorescence and bright images of GSH intensity in the control and rotenone-treated groups. Blue = 404nm, red = 526nm. (B) Relative fluorescence intensity of GSH in the control (n = 9) and rotenone-treated groups (n = 12). Con., control group. Rot., 3μM rotenone-exposed group. ****(P < 0.0001) (TIF) [file pone.0277477.s002.tif]

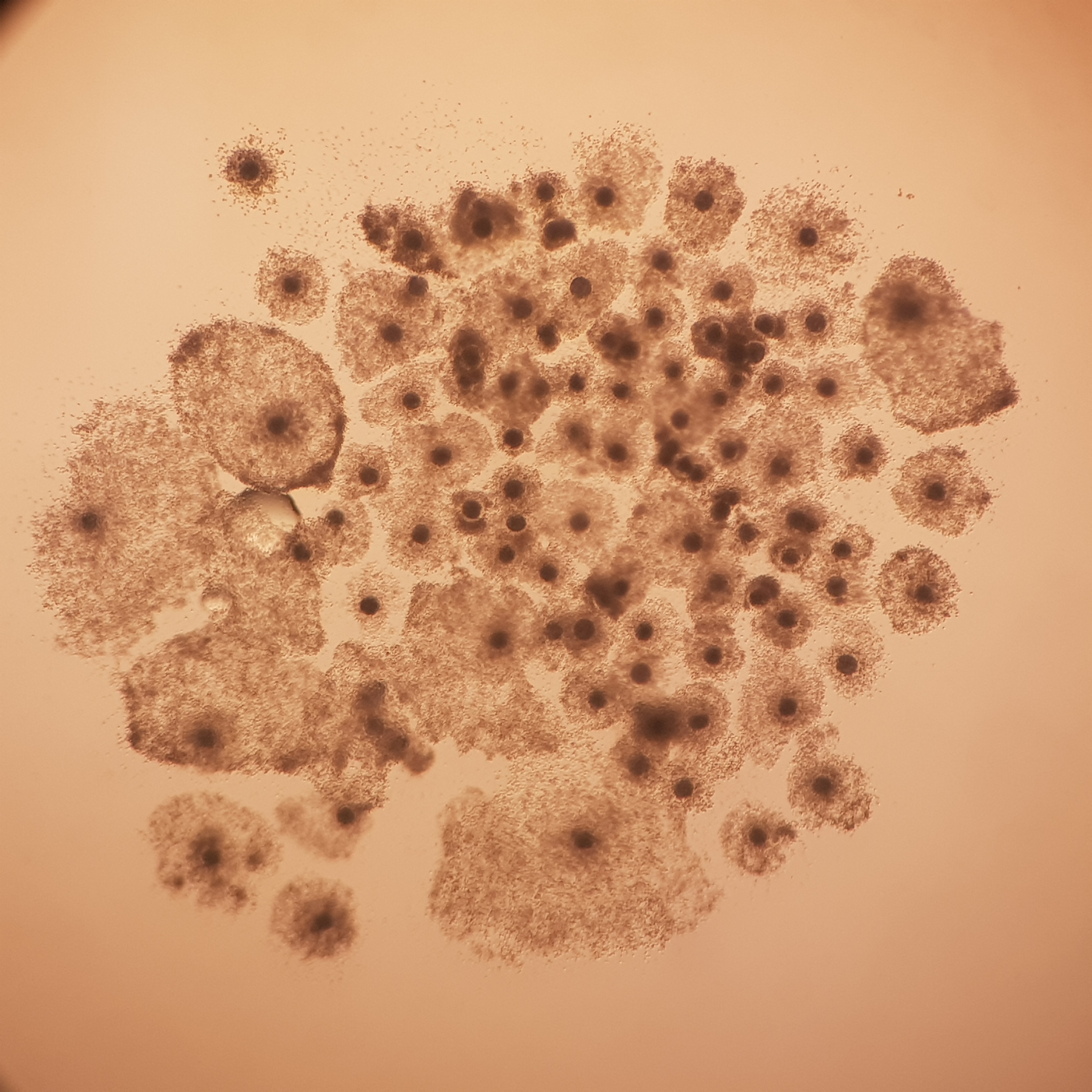

Supplement: S2 Raw images — (ZIP) [file pone.0277477.s004.zip › Fig1a_con.jpg]

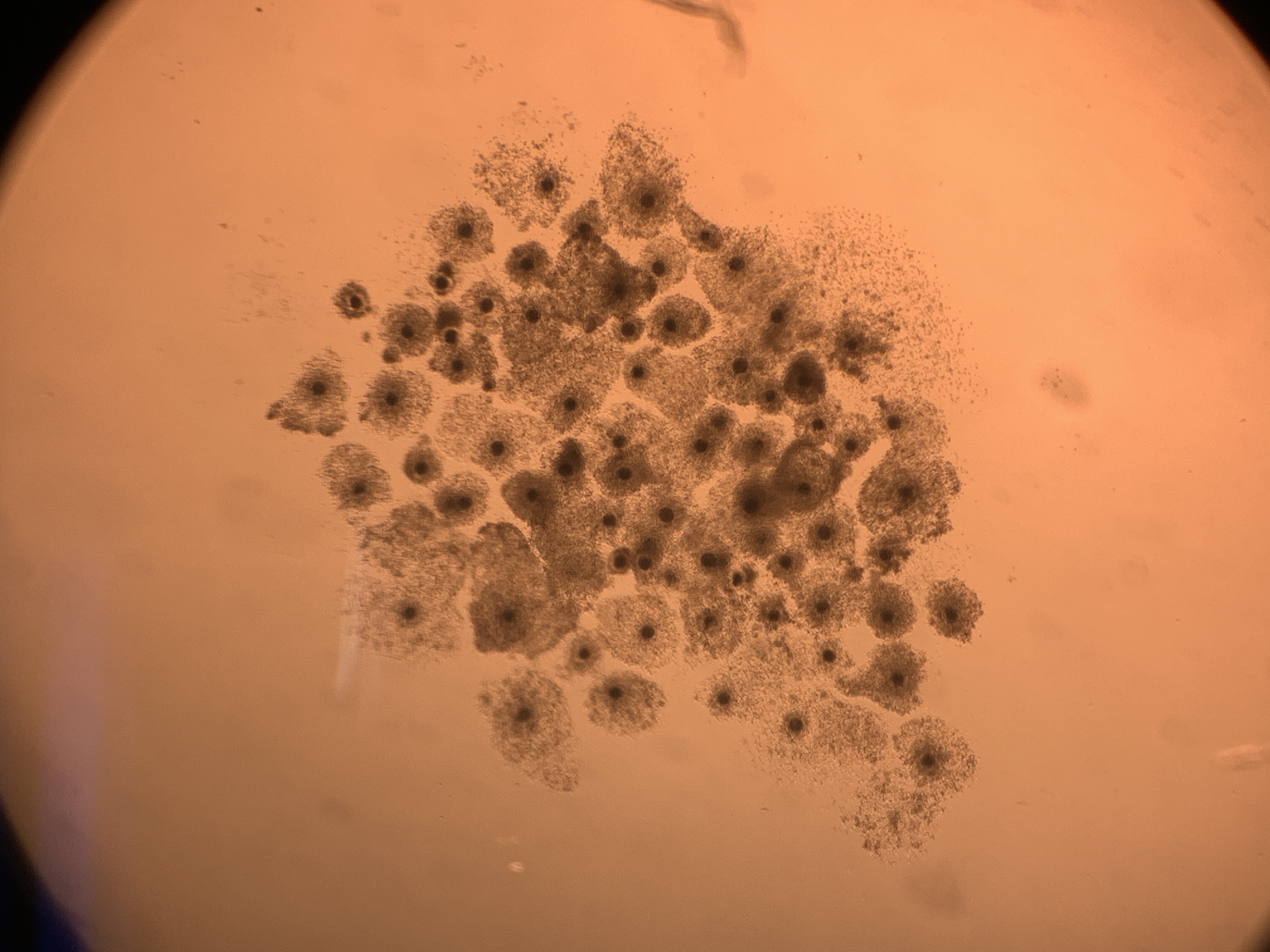

Supplement: S2 Raw images — (ZIP) [file pone.0277477.s004.zip › Fig1a_rot2.JPG]

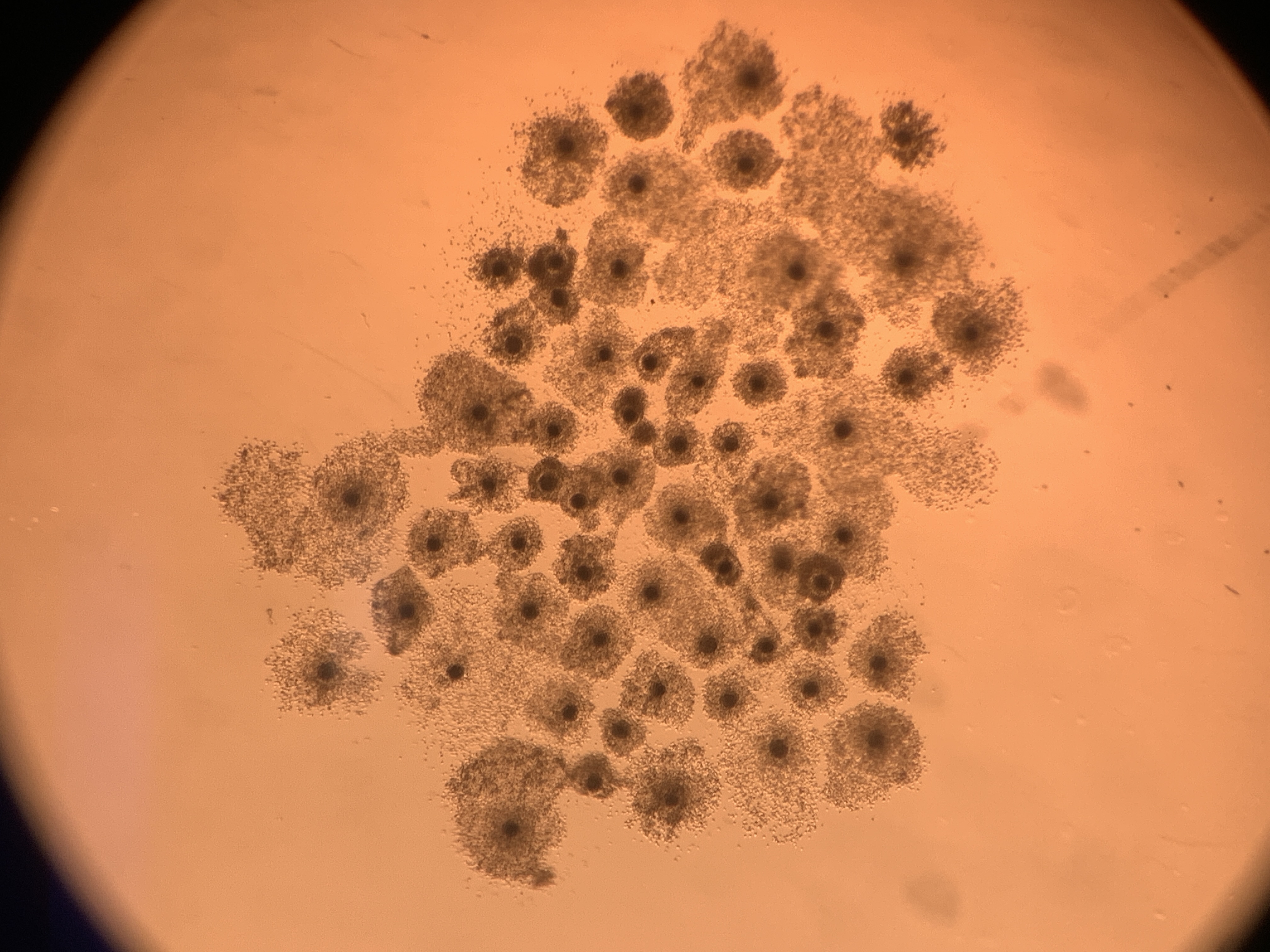

Supplement: S2 Raw images — (ZIP) [file pone.0277477.s004.zip › Fig1a_rot3.JPG]

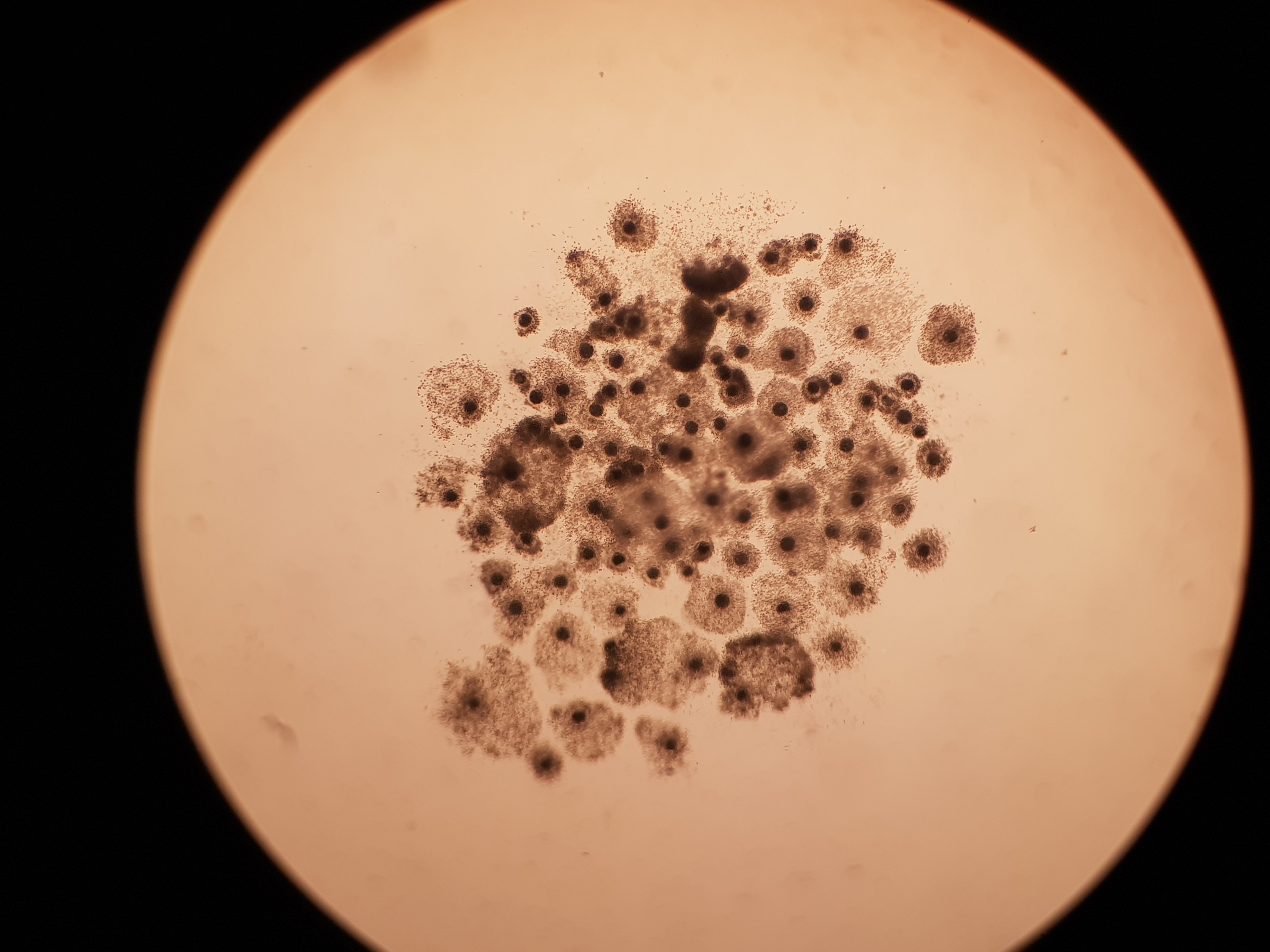

Supplement: S2 Raw images — (ZIP) [file pone.0277477.s004.zip › Fig1a_rot5.jpg]

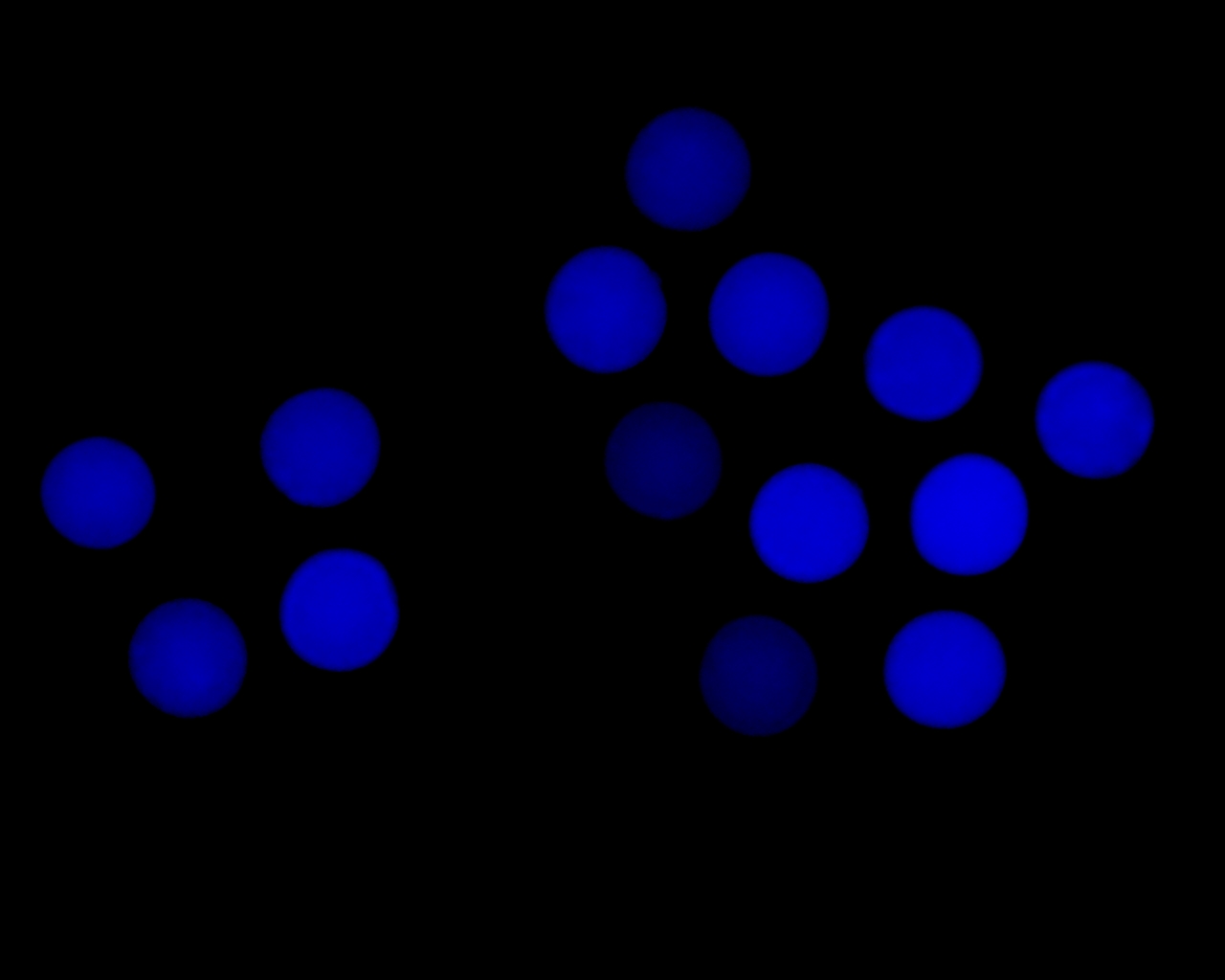

Supplement: S3 Raw images — (ZIP) [file pone.0277477.s005.zip › Fig2a_GSH_con.jpg]

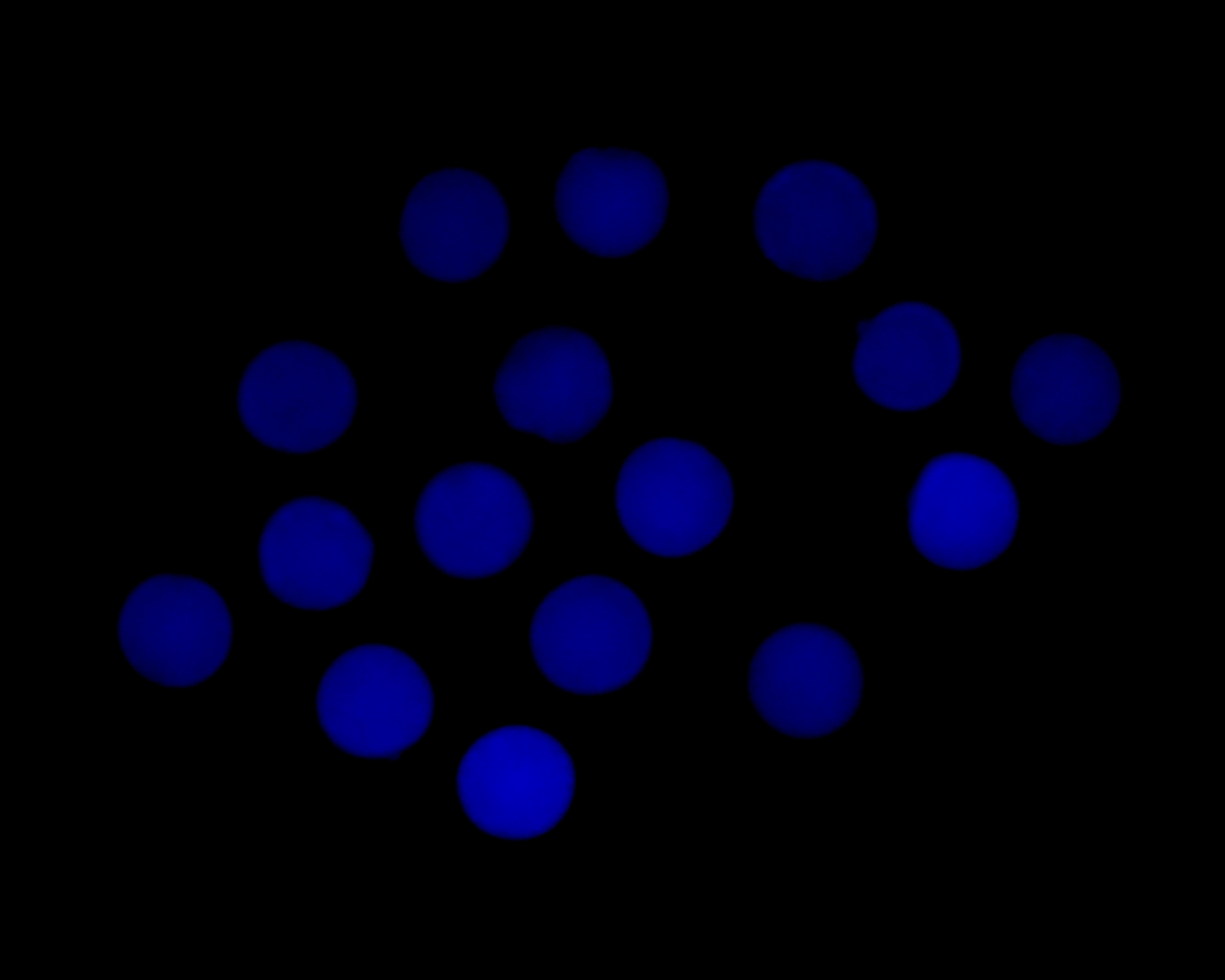

Supplement: S3 Raw images — (ZIP) [file pone.0277477.s005.zip › Fig2a_GSH_rot.jpg]

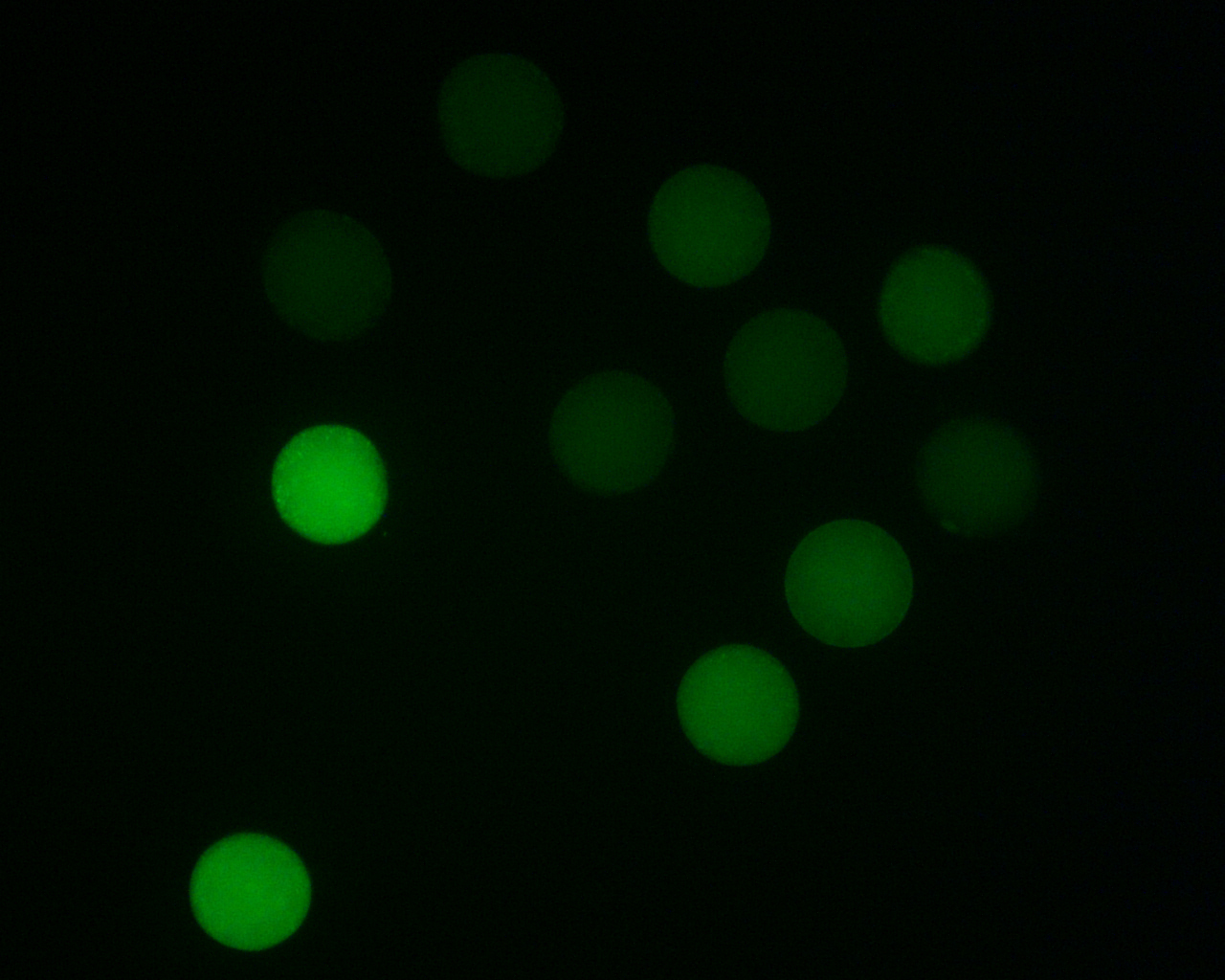

Supplement: S3 Raw images — (ZIP) [file pone.0277477.s005.zip › Fig2a_ROS_con.jpg]

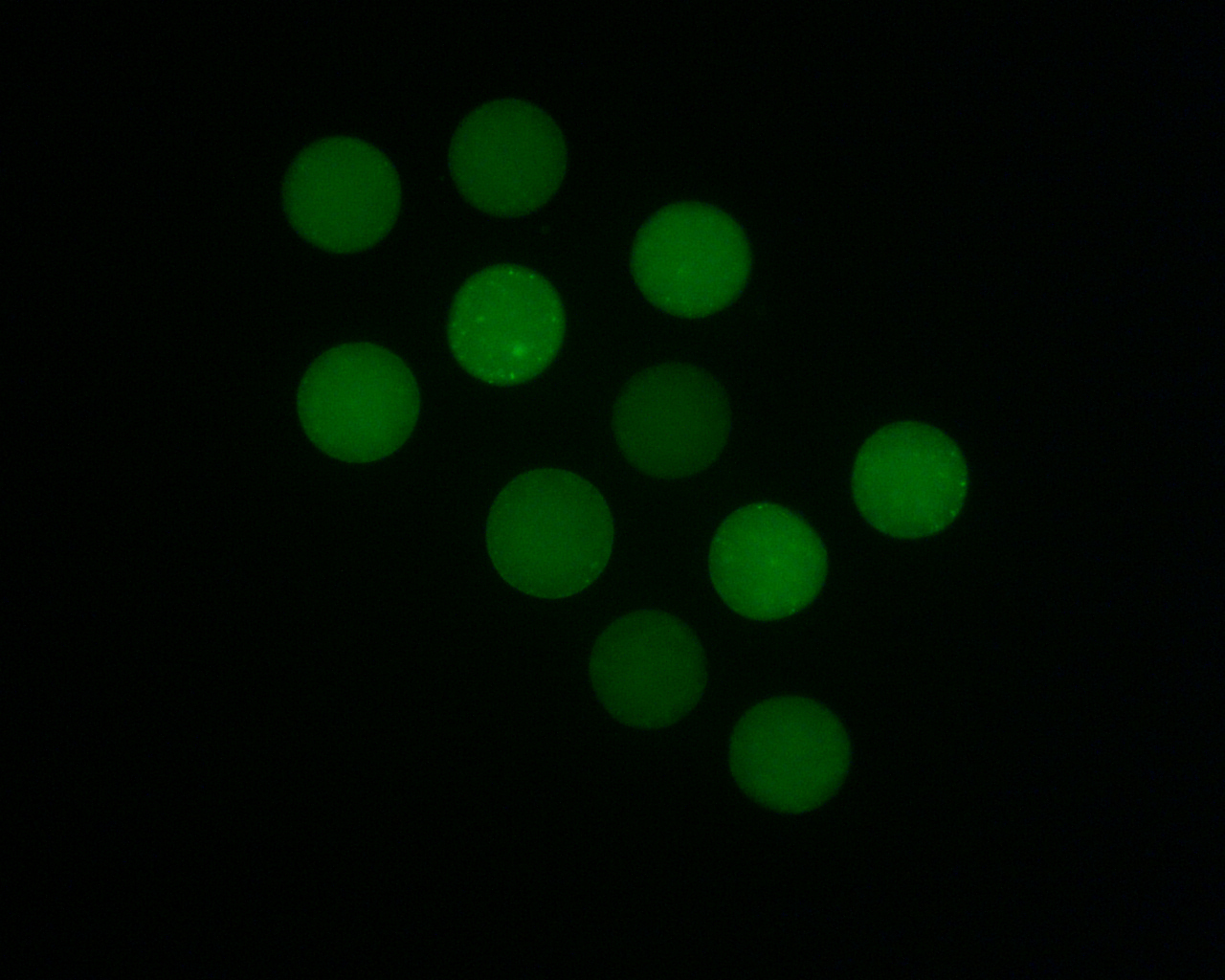

Supplement: S3 Raw images — (ZIP) [file pone.0277477.s005.zip › Fig2a_ROS_rot.jpg]

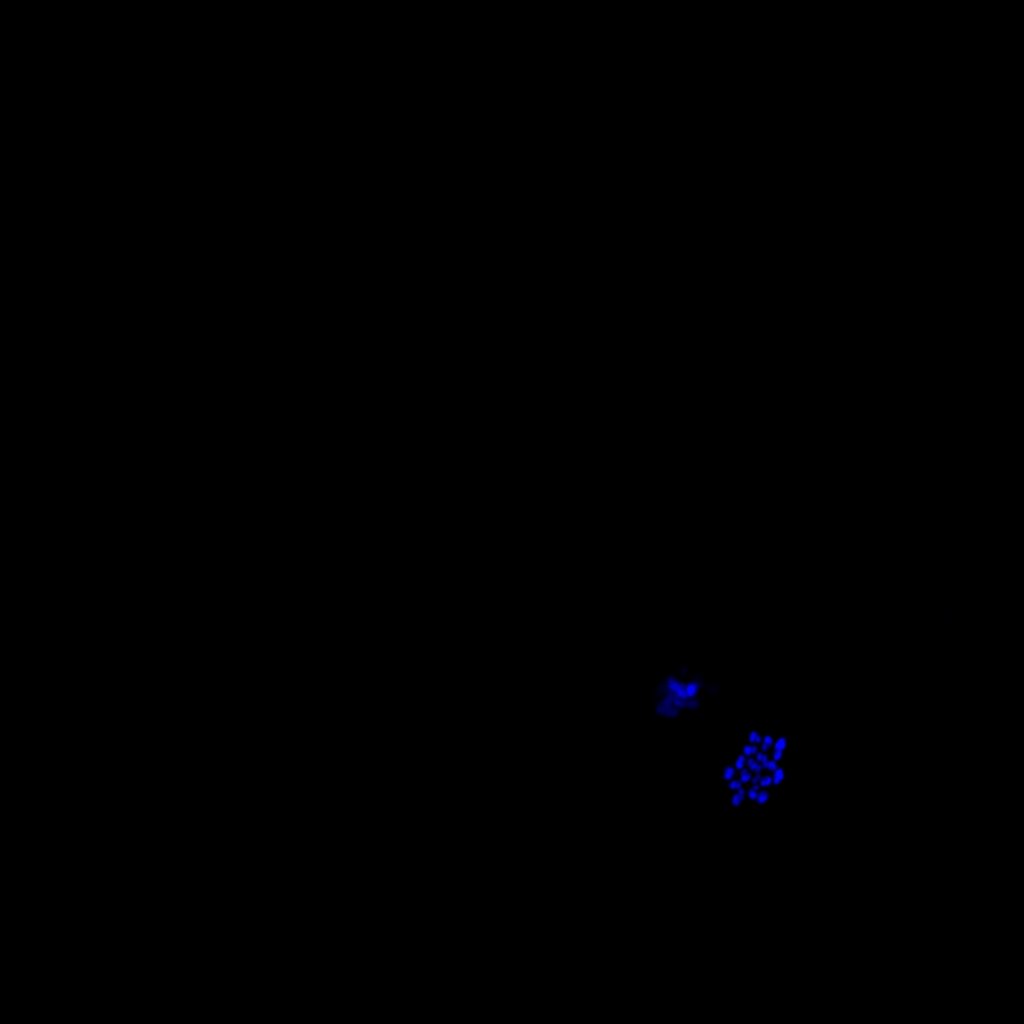

Supplement: S4 Raw images — (ZIP) [file pone.0277477.s006.zip › Fig2c_con_c1.jpg]

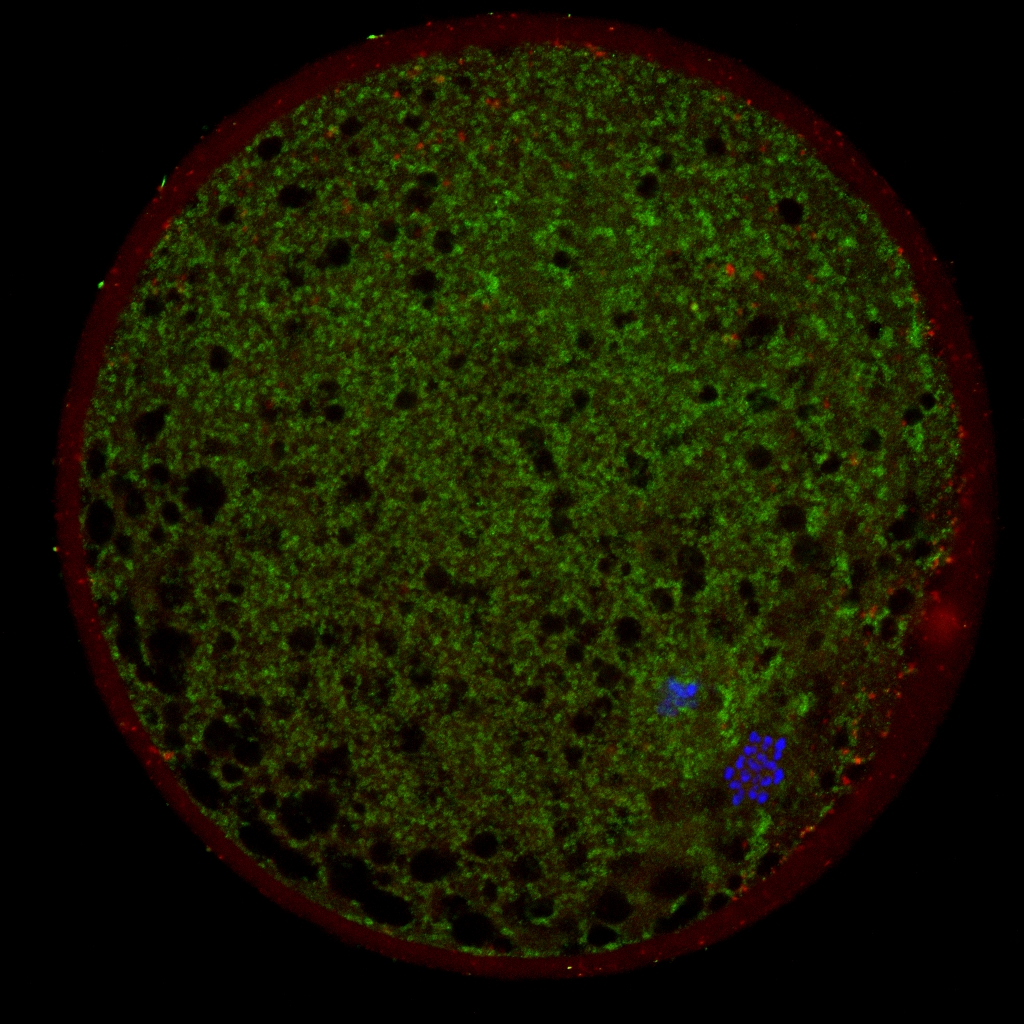

Supplement: S4 Raw images — (ZIP) [file pone.0277477.s006.zip › Fig2c_con_c1+2+3.jpg]

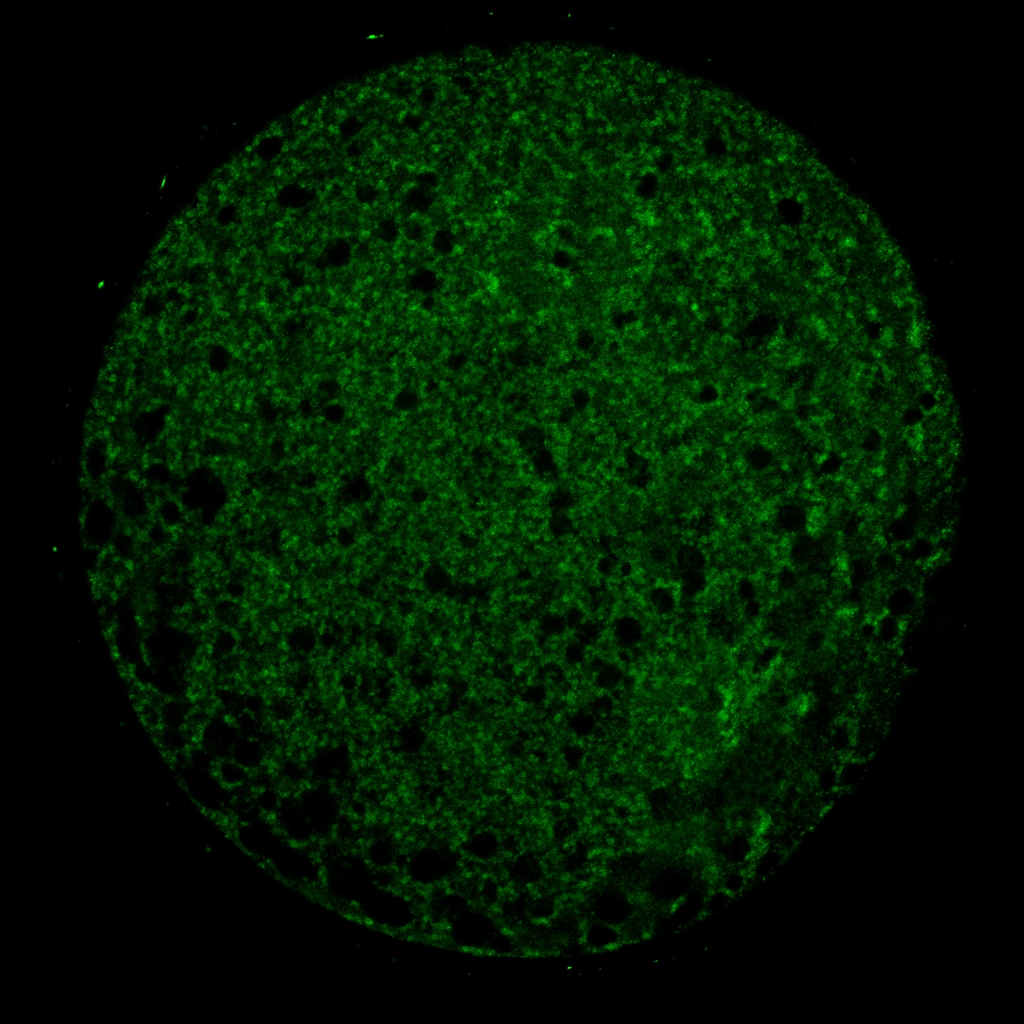

Supplement: S4 Raw images — (ZIP) [file pone.0277477.s006.zip › Fig2c_con_c2.jpg]

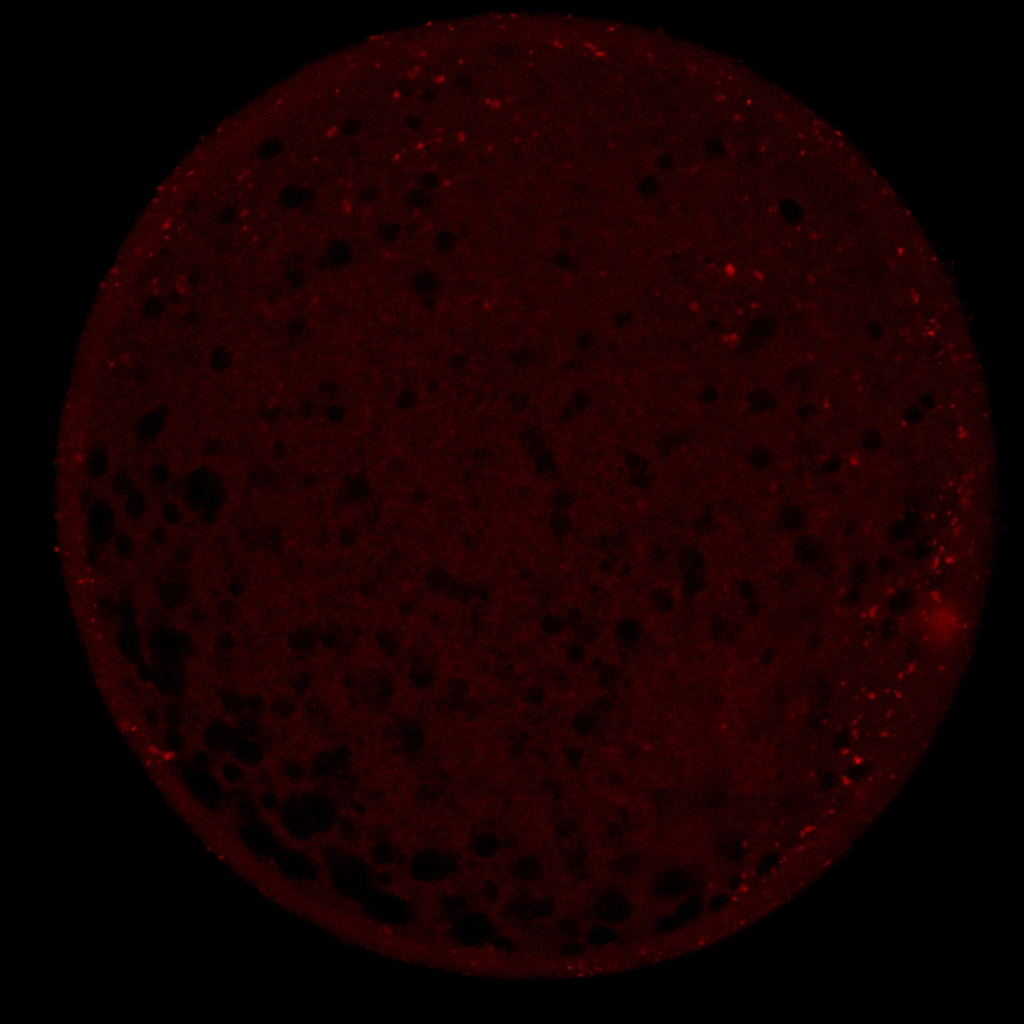

Supplement: S4 Raw images — (ZIP) [file pone.0277477.s006.zip › Fig2c_con_c3.jpg]

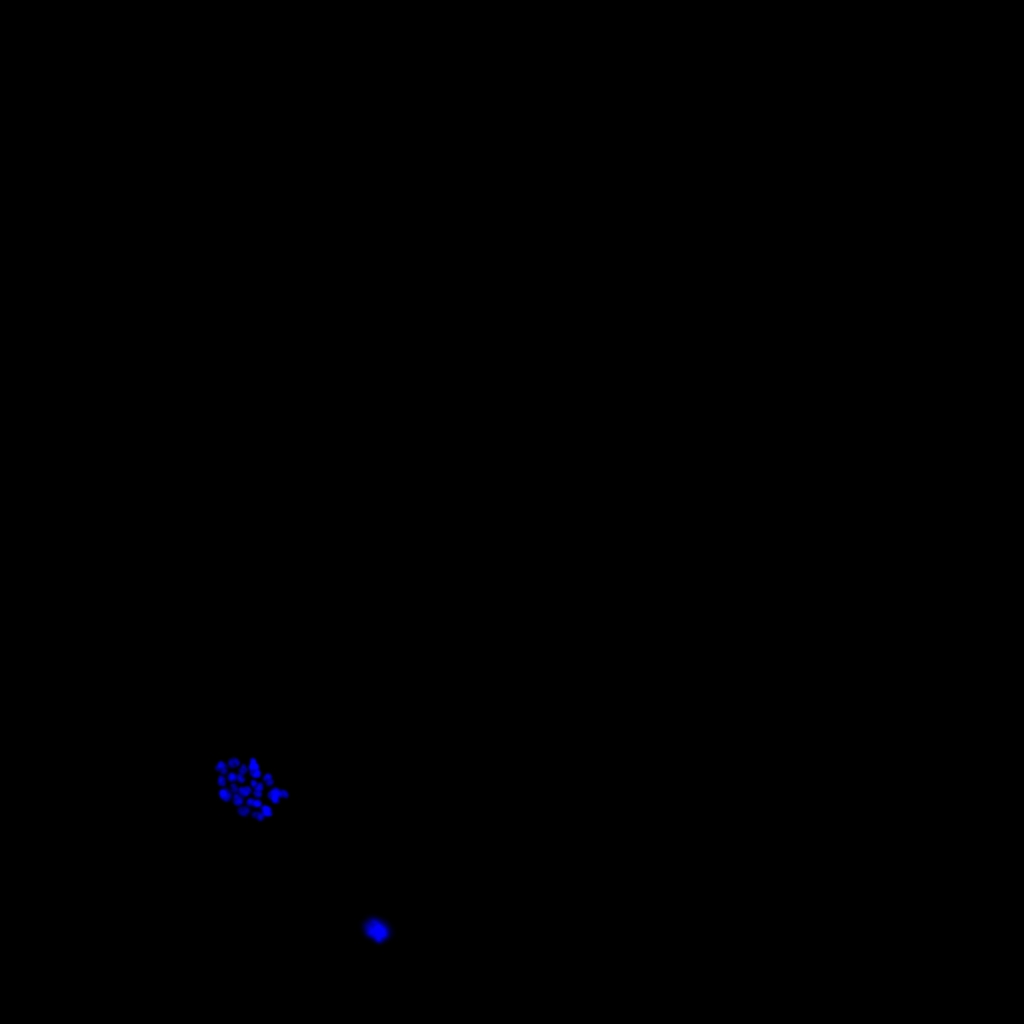

Supplement: S4 Raw images — (ZIP) [file pone.0277477.s006.zip › Fig2c_rot_c1.jpg]

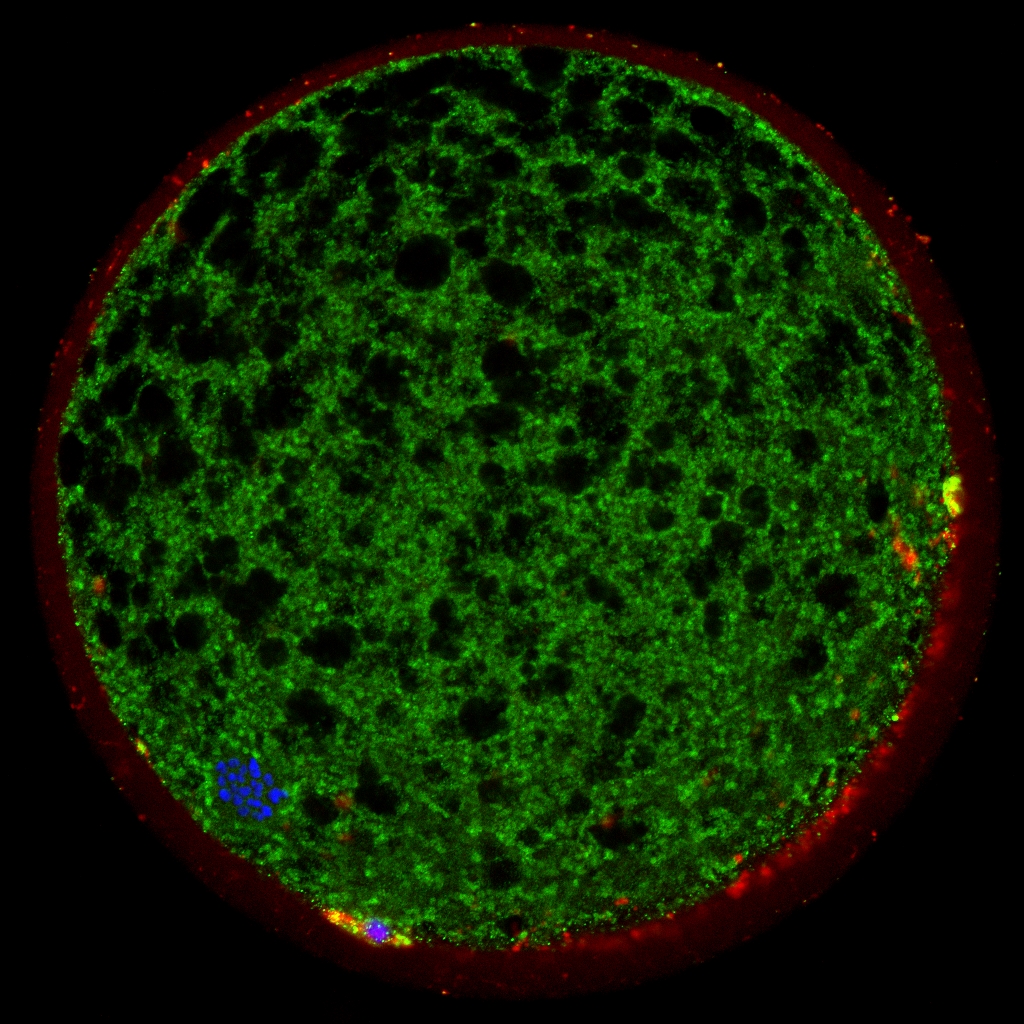

Supplement: S4 Raw images — (ZIP) [file pone.0277477.s006.zip › Fig2c_rot_c1+2+3.jpg]

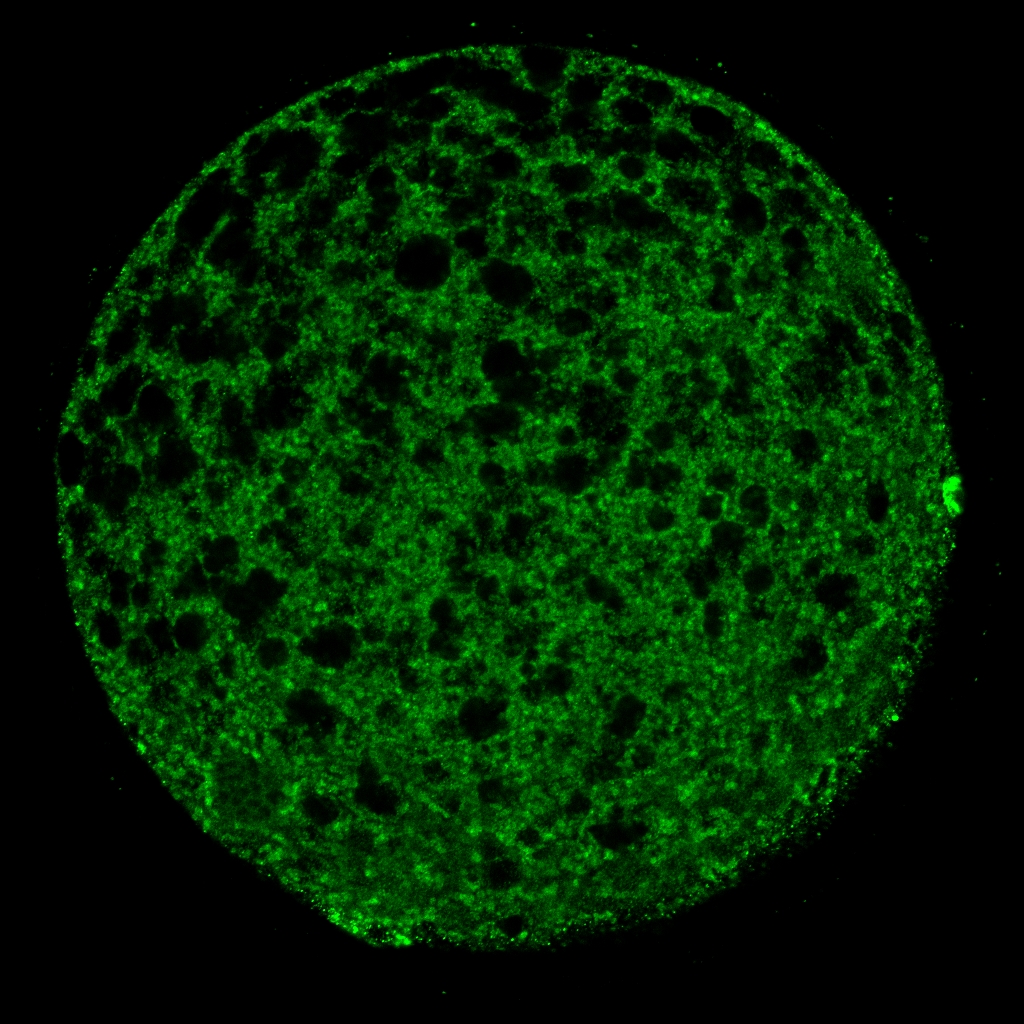

Supplement: S4 Raw images — (ZIP) [file pone.0277477.s006.zip › Fig2c_rot_c2.jpg]

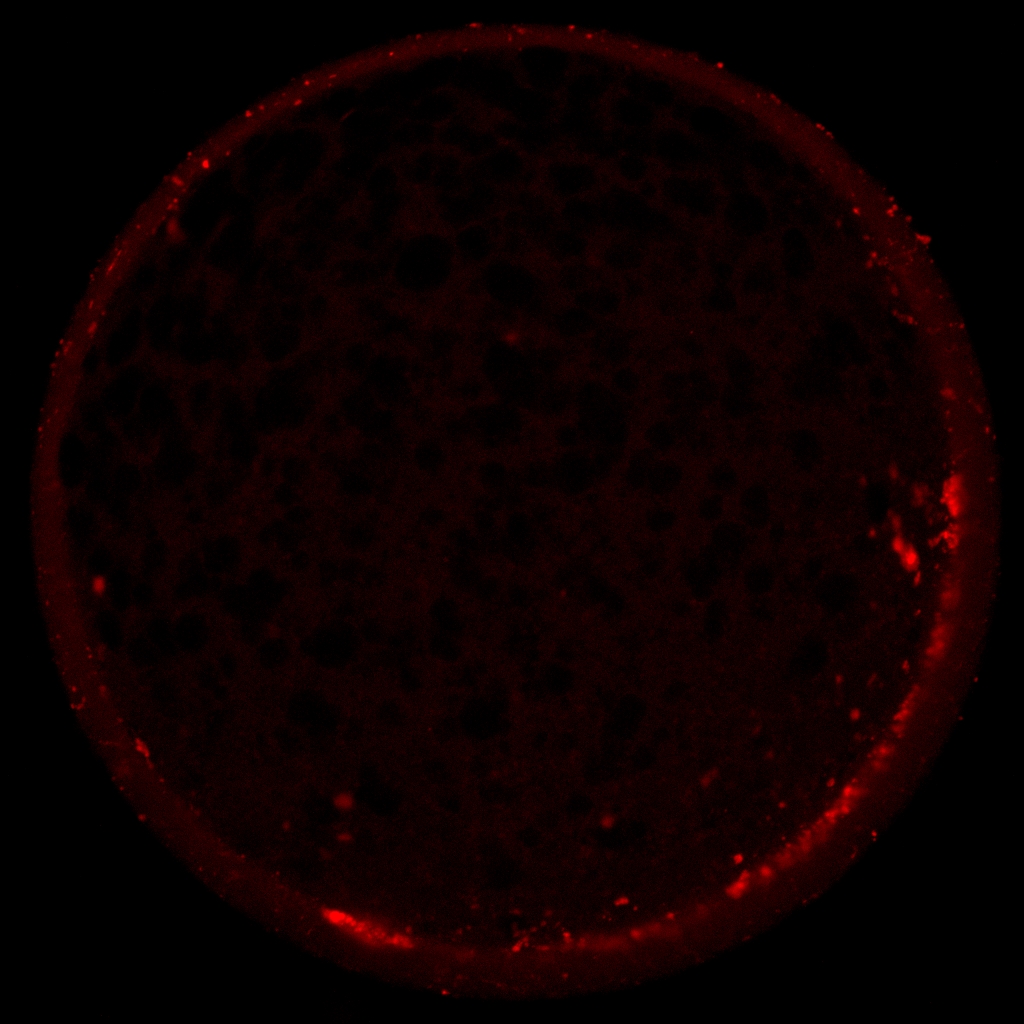

Supplement: S4 Raw images — (ZIP) [file pone.0277477.s006.zip › Fig2c_rot_c3.jpg]

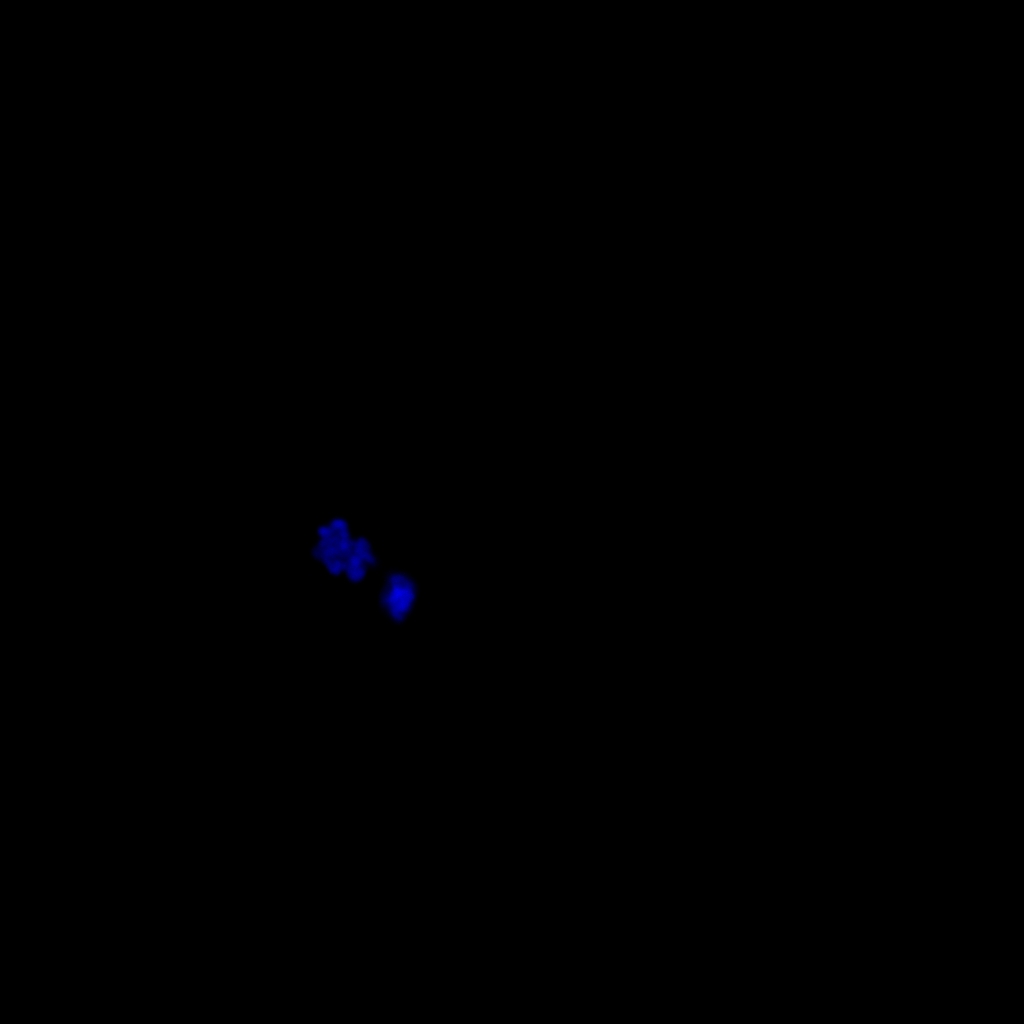

Supplement: S5 Raw images — (ZIP) [file pone.0277477.s007.zip › Fig3a_con_c1.jpg]

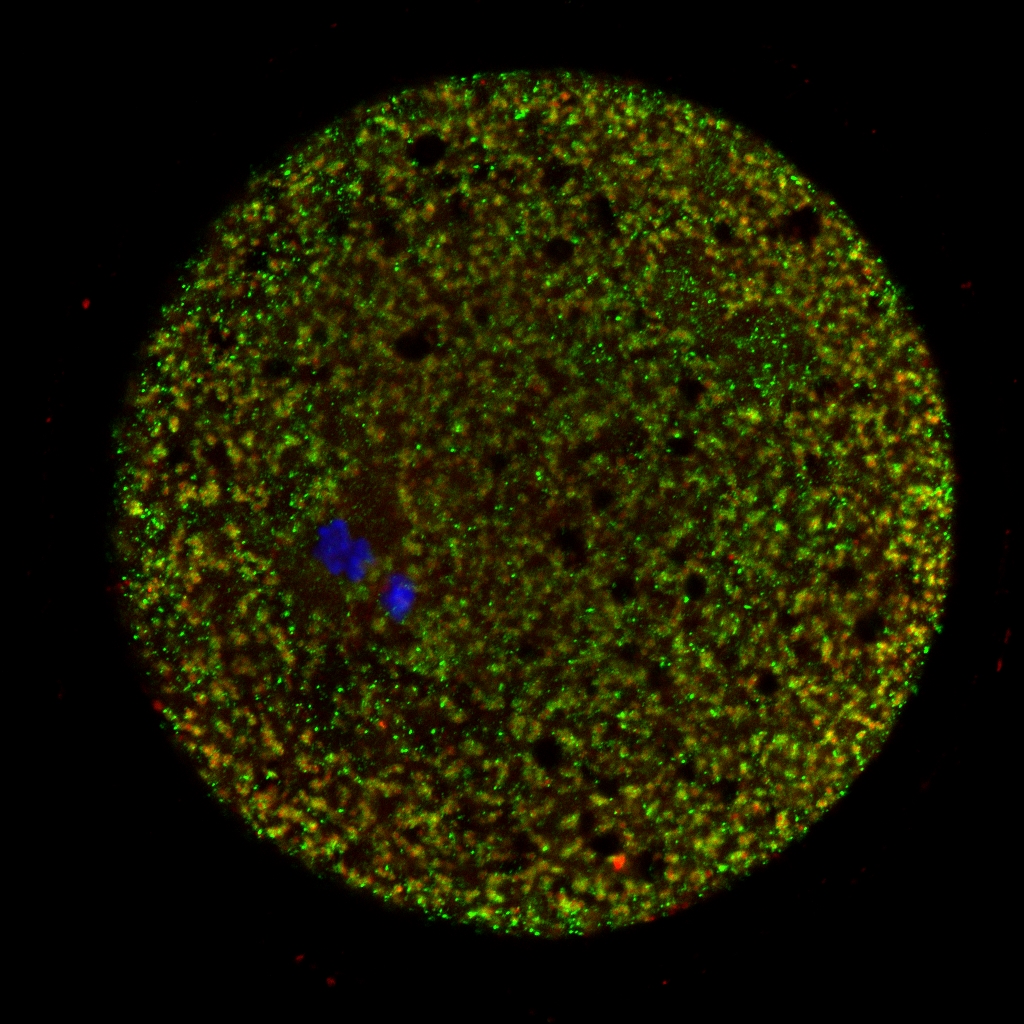

Supplement: S5 Raw images — (ZIP) [file pone.0277477.s007.zip › Fig3a_con_c1+2+3.jpg]

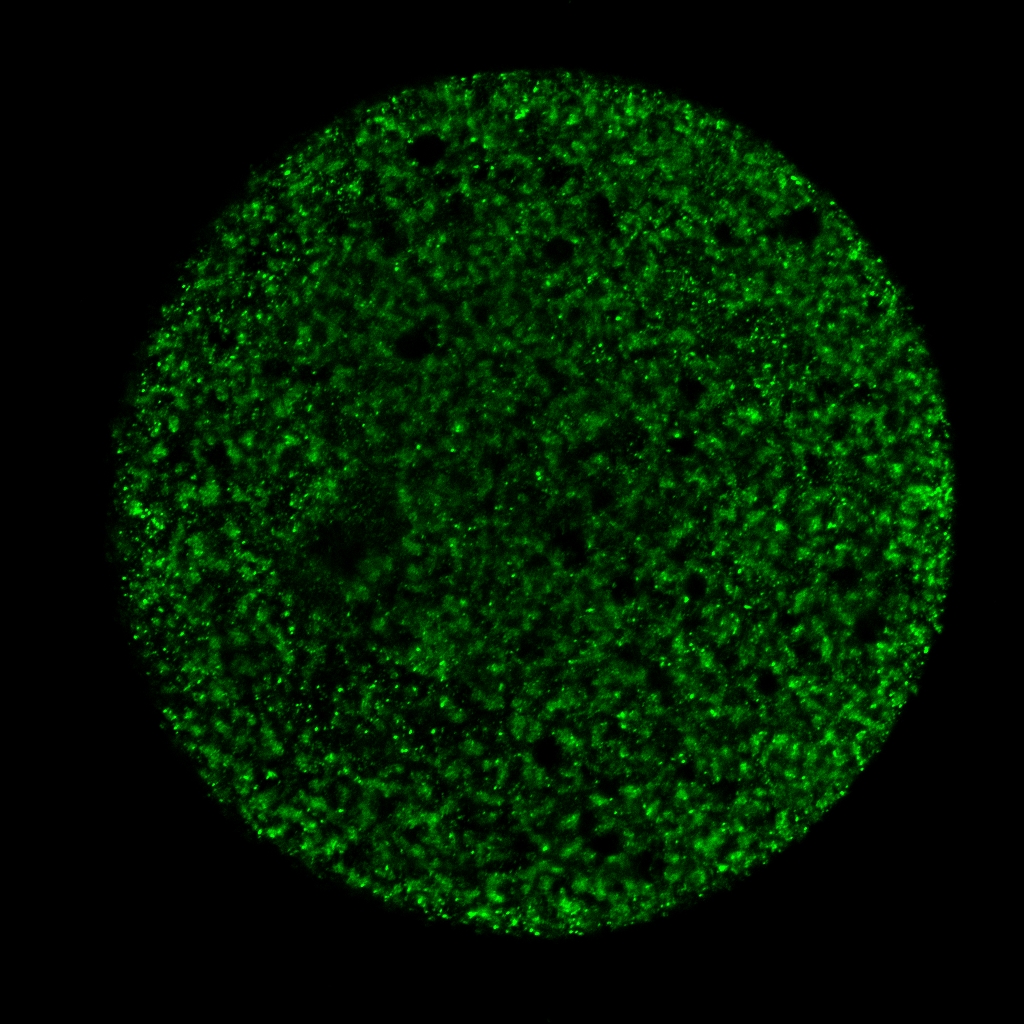

Supplement: S5 Raw images — (ZIP) [file pone.0277477.s007.zip › Fig3a_con_c2.jpg]

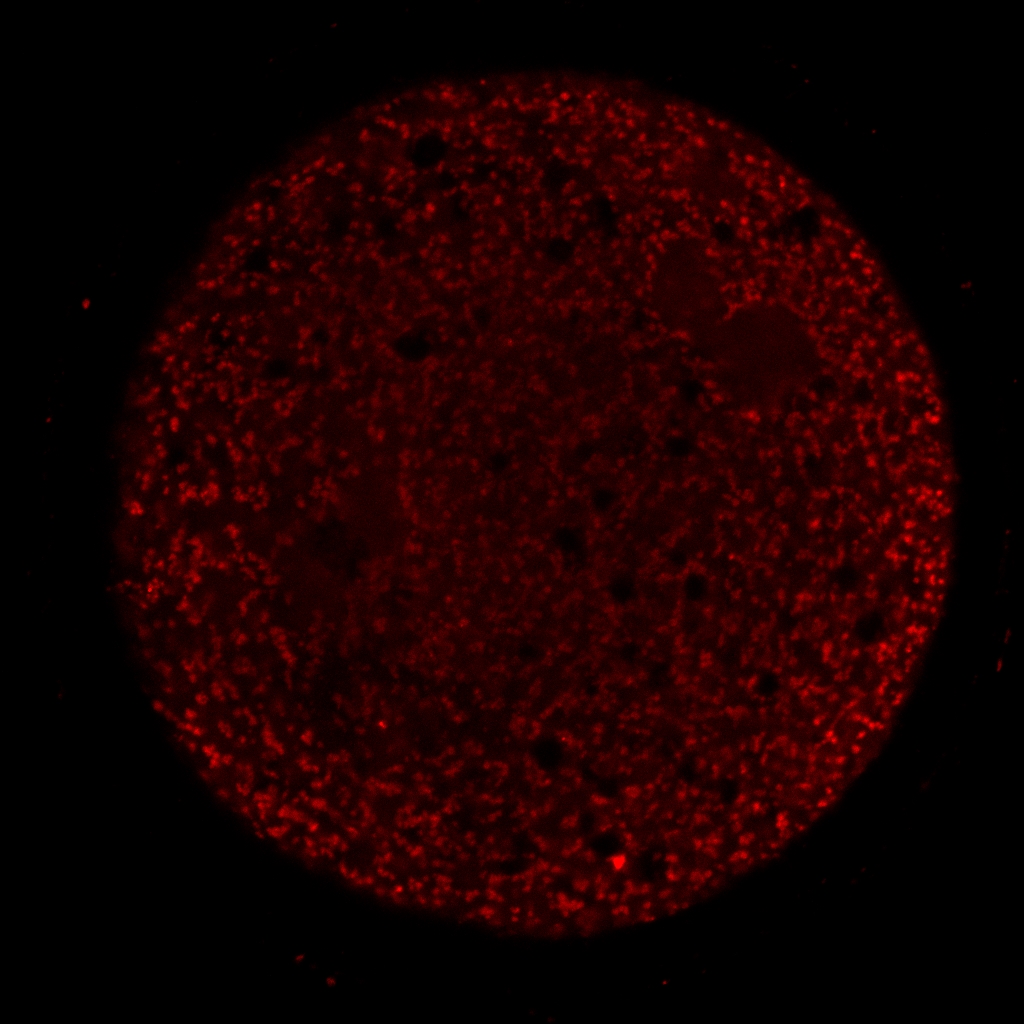

Supplement: S5 Raw images — (ZIP) [file pone.0277477.s007.zip › Fig3a_con_c3.jpg]

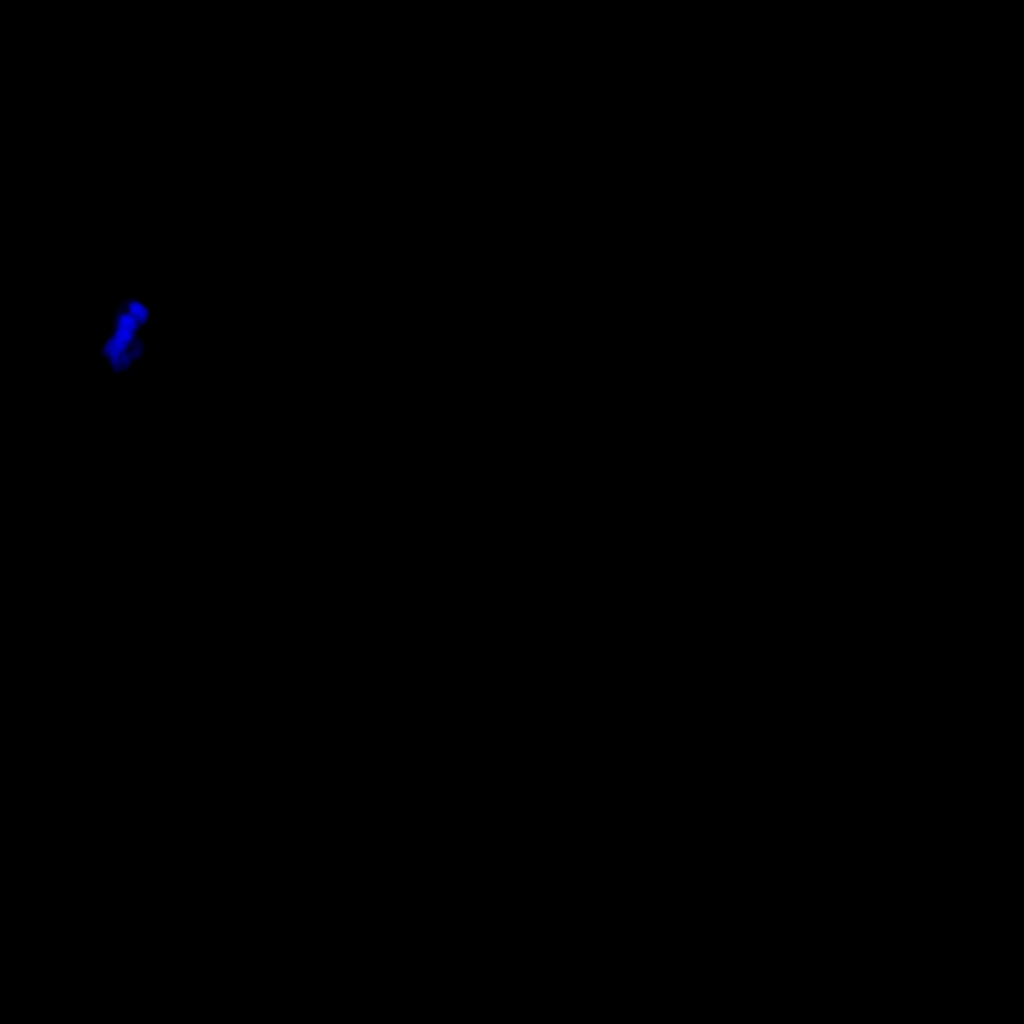

Supplement: S5 Raw images — (ZIP) [file pone.0277477.s007.zip › Fig3a_rot_c1.jpg]

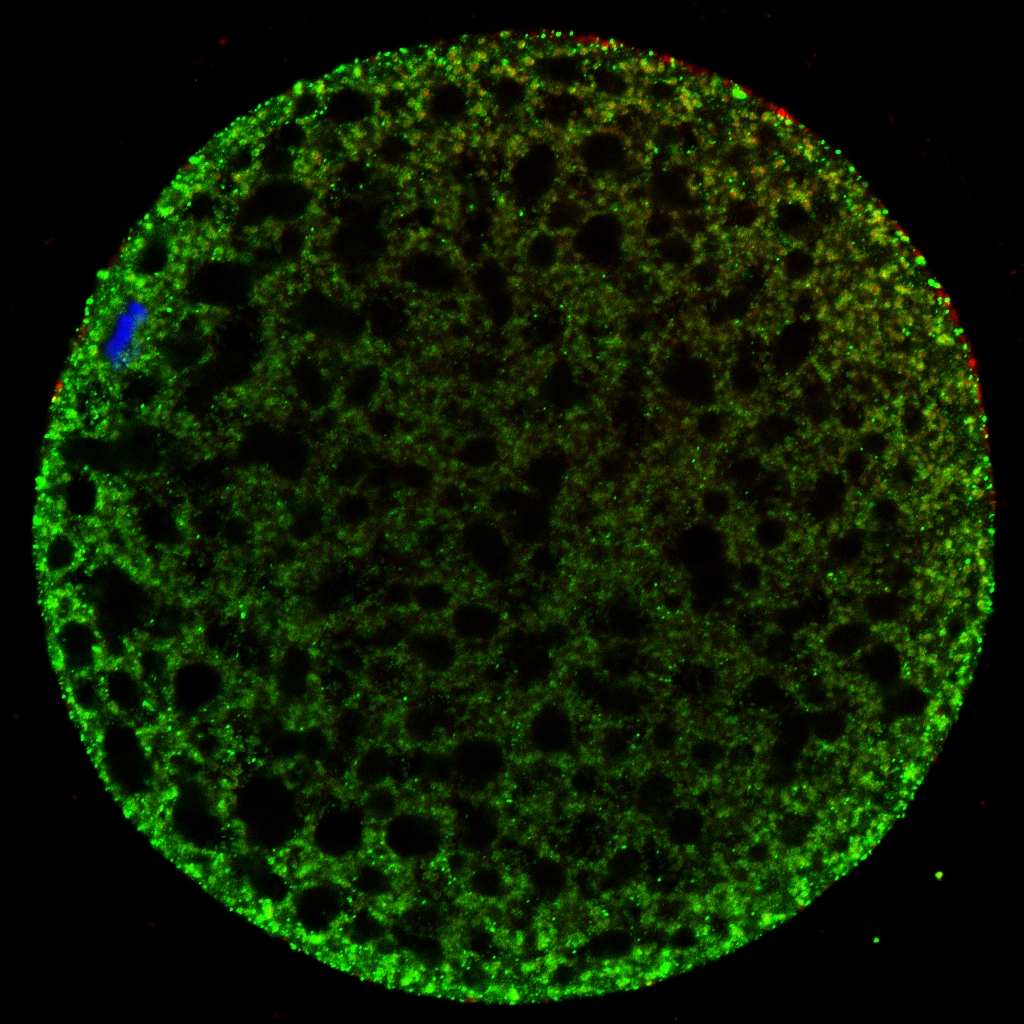

Supplement: S5 Raw images — (ZIP) [file pone.0277477.s007.zip › Fig3a_rot_c1+2+3.jpg]

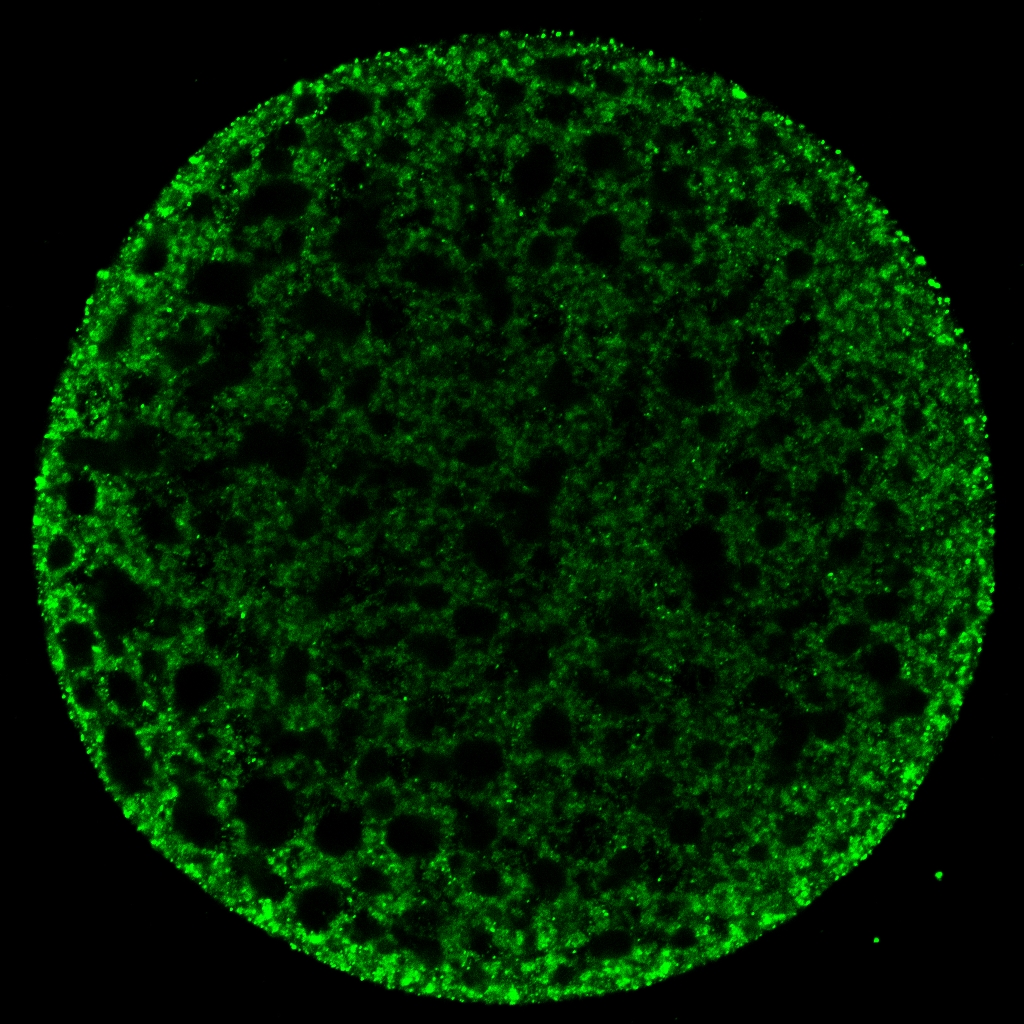

Supplement: S5 Raw images — (ZIP) [file pone.0277477.s007.zip › Fig3a_rot_c2.jpg]

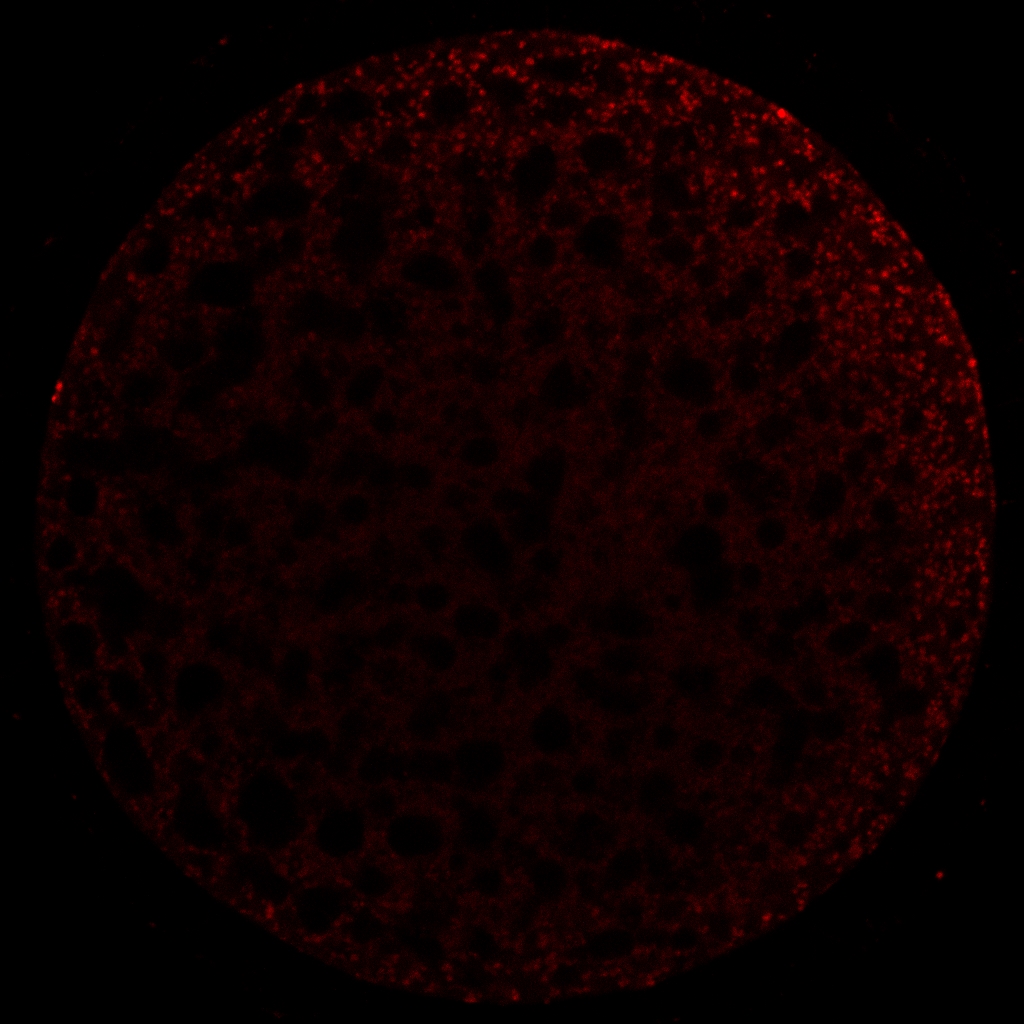

Supplement: S5 Raw images — (ZIP) [file pone.0277477.s007.zip › Fig3a_rot_c3.jpg]

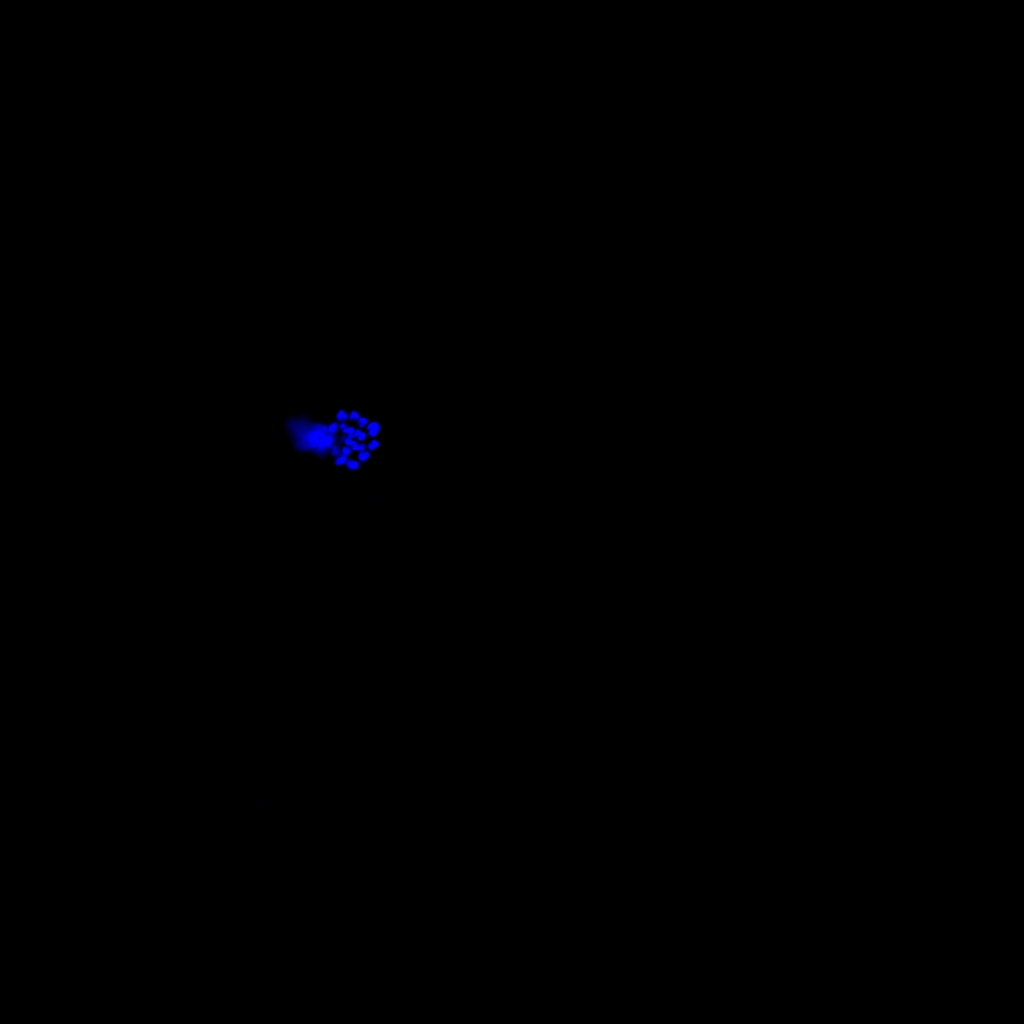

Supplement: S6 Raw images — (ZIP) [file pone.0277477.s008.zip › Fig4b_con_c1.jpg]

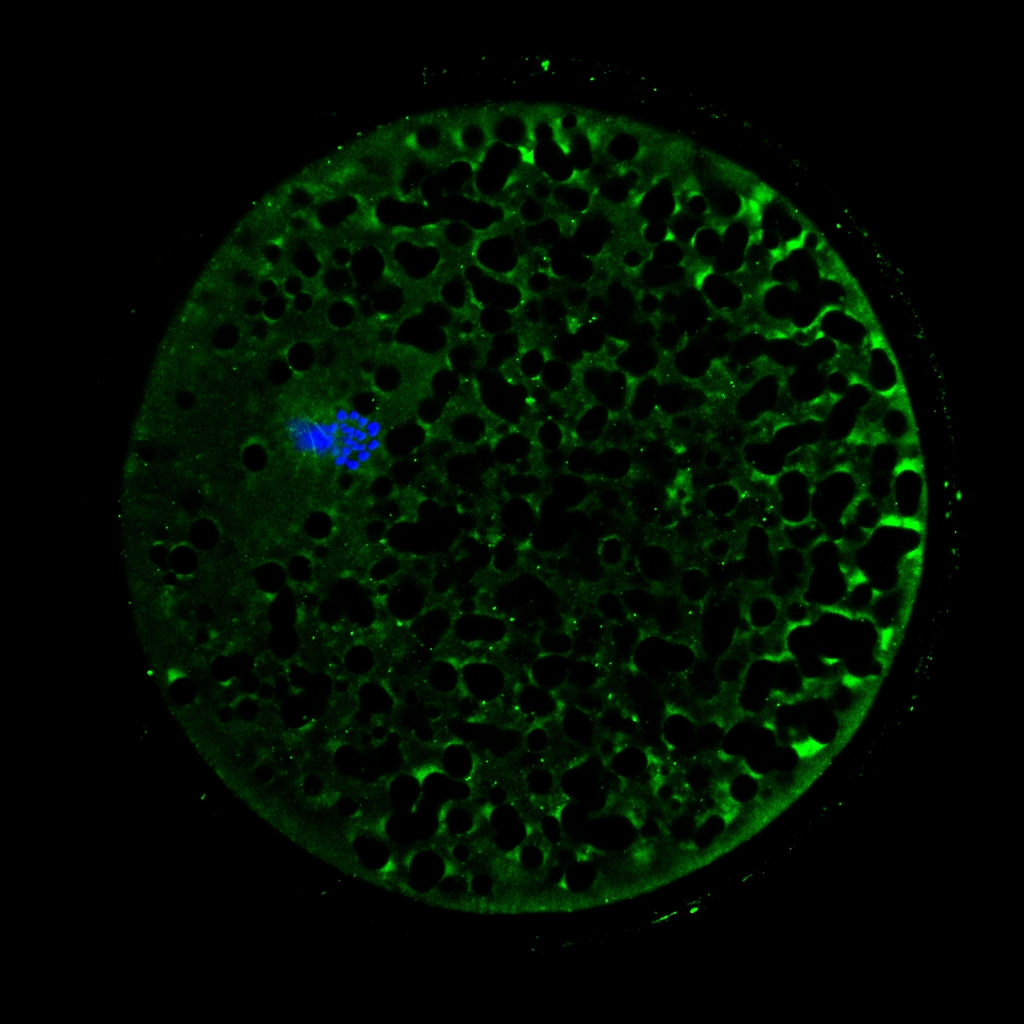

Supplement: S6 Raw images — (ZIP) [file pone.0277477.s008.zip › Fig4b_con_c1+2.jpg]

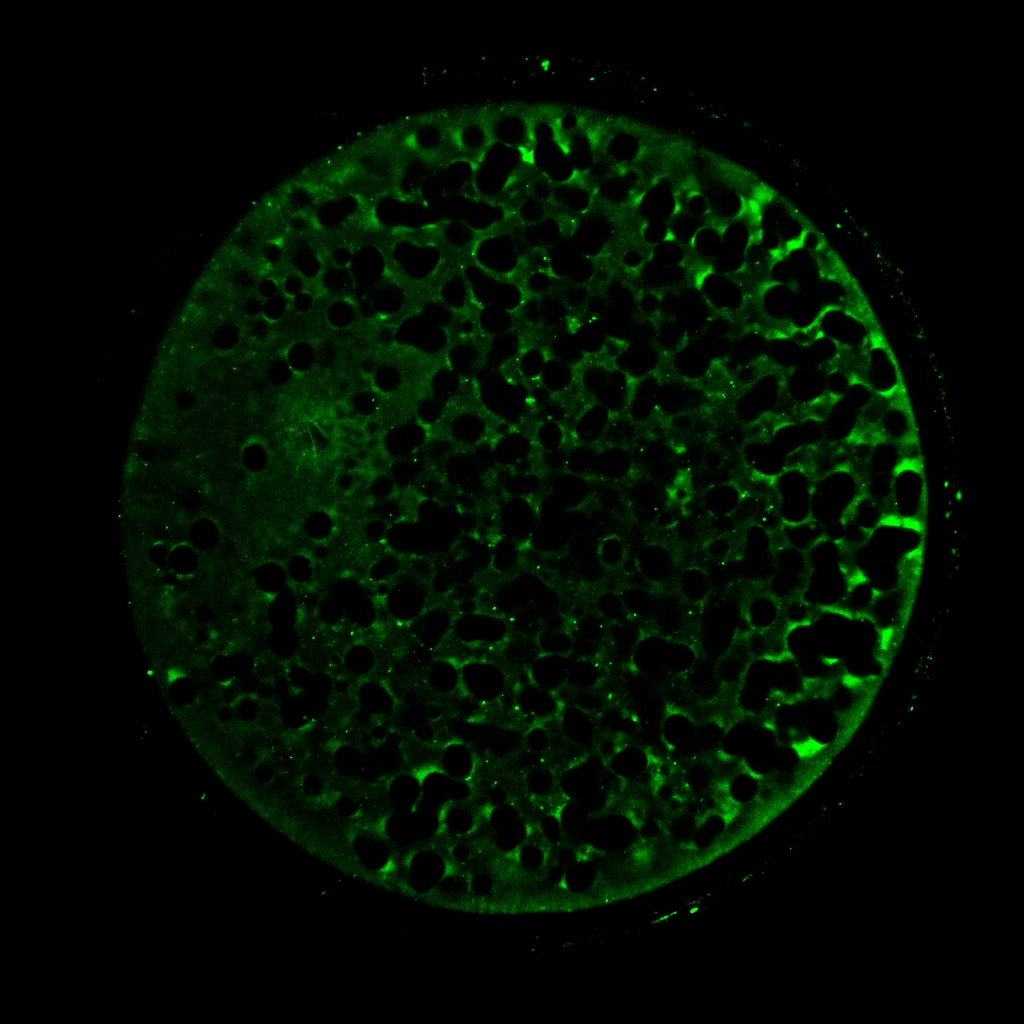

Supplement: S6 Raw images — (ZIP) [file pone.0277477.s008.zip › Fig4b_con_c2.jpg]

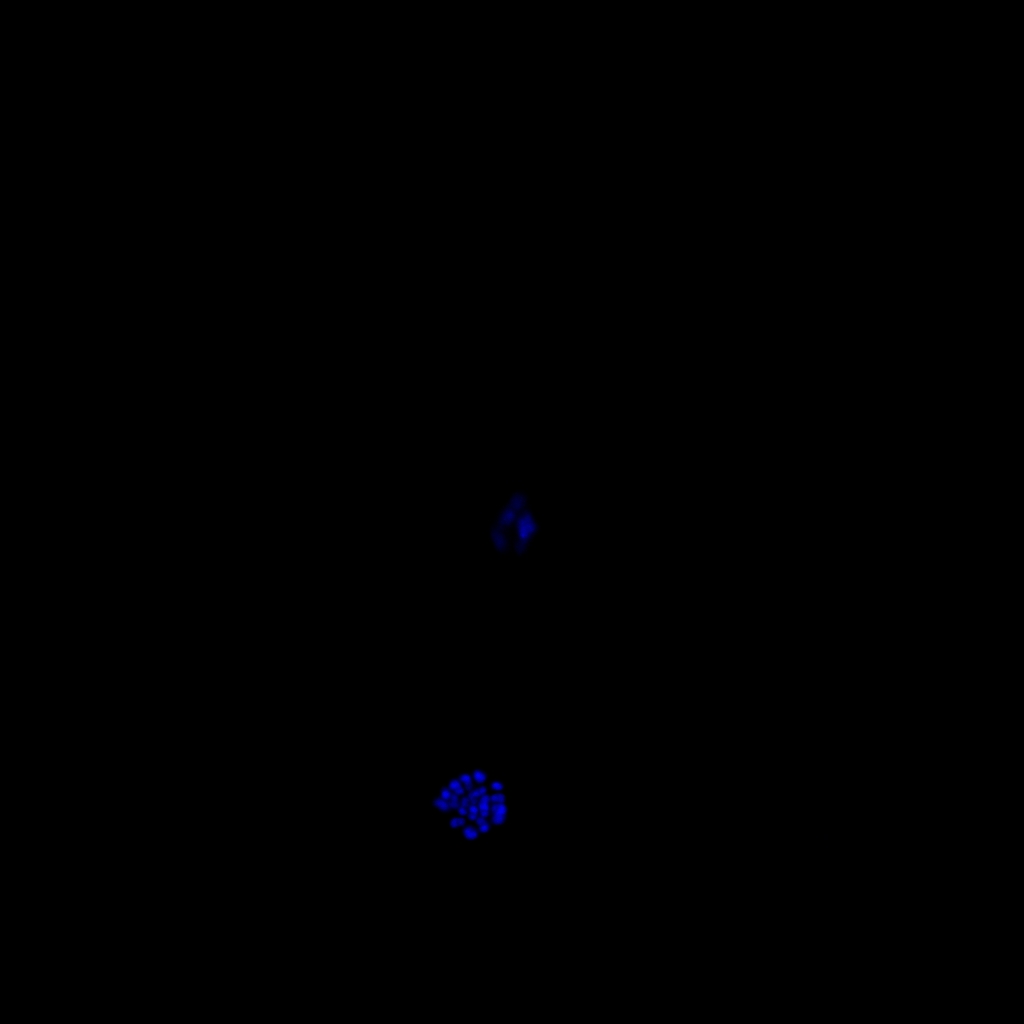

Supplement: S6 Raw images — (ZIP) [file pone.0277477.s008.zip › Fig4b_rot_c1.jpg]

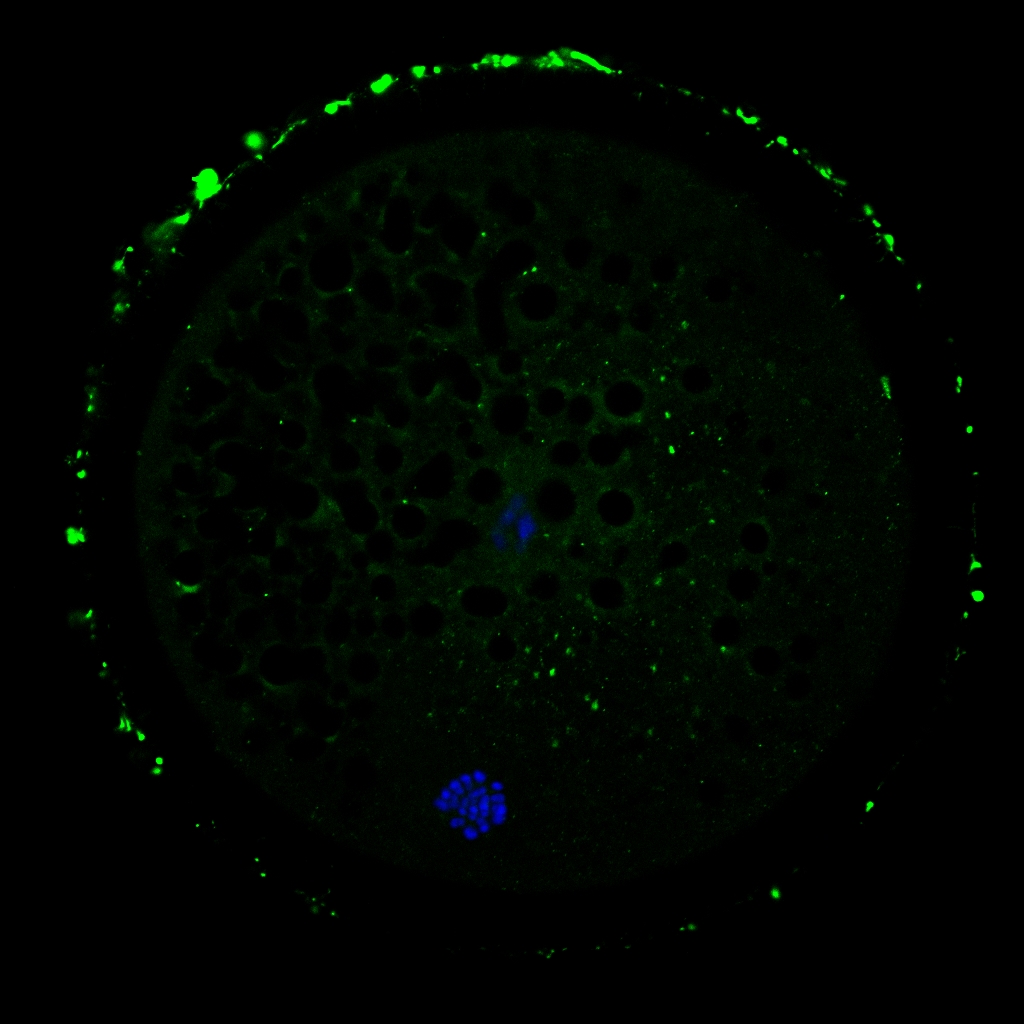

Supplement: S6 Raw images — (ZIP) [file pone.0277477.s008.zip › Fig4b_rot_c1+2.jpg]

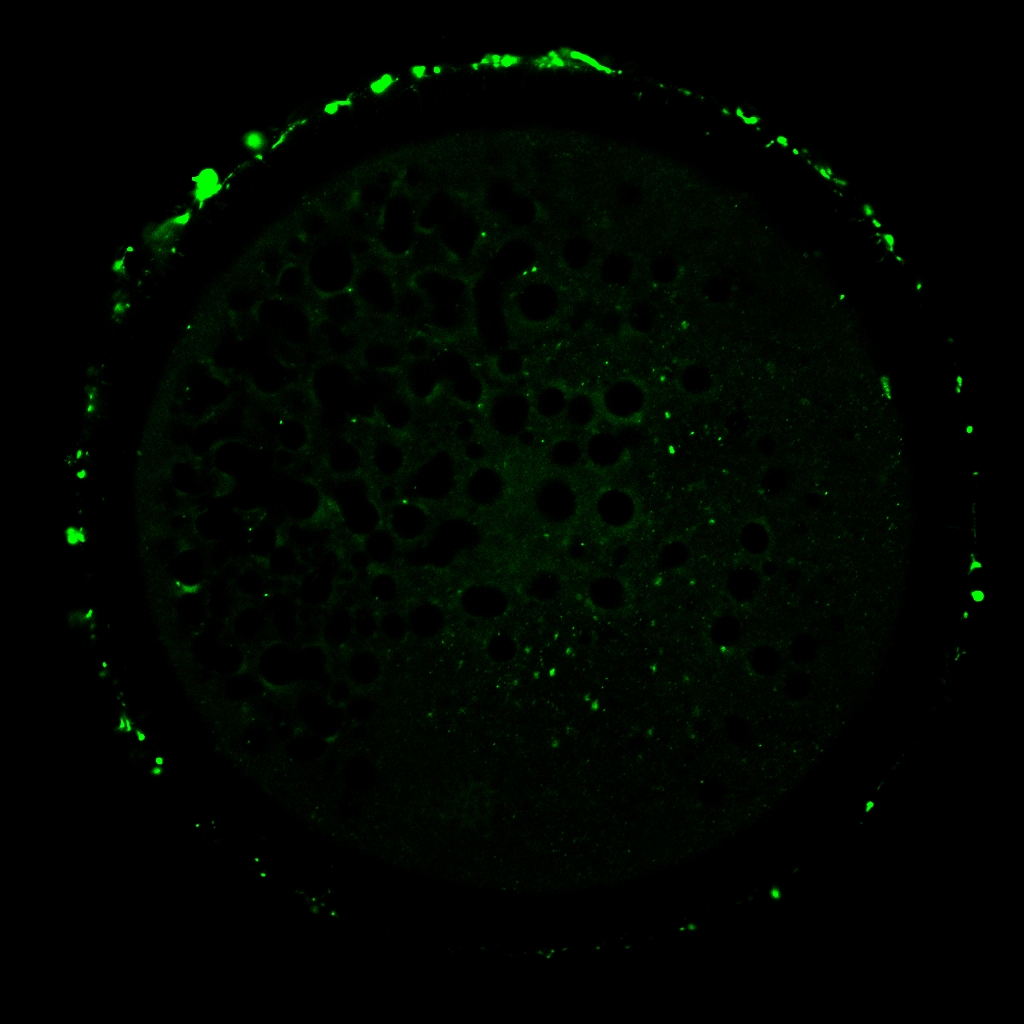

Supplement: S6 Raw images — (ZIP) [file pone.0277477.s008.zip › Fig4b_rot_c2.jpg]

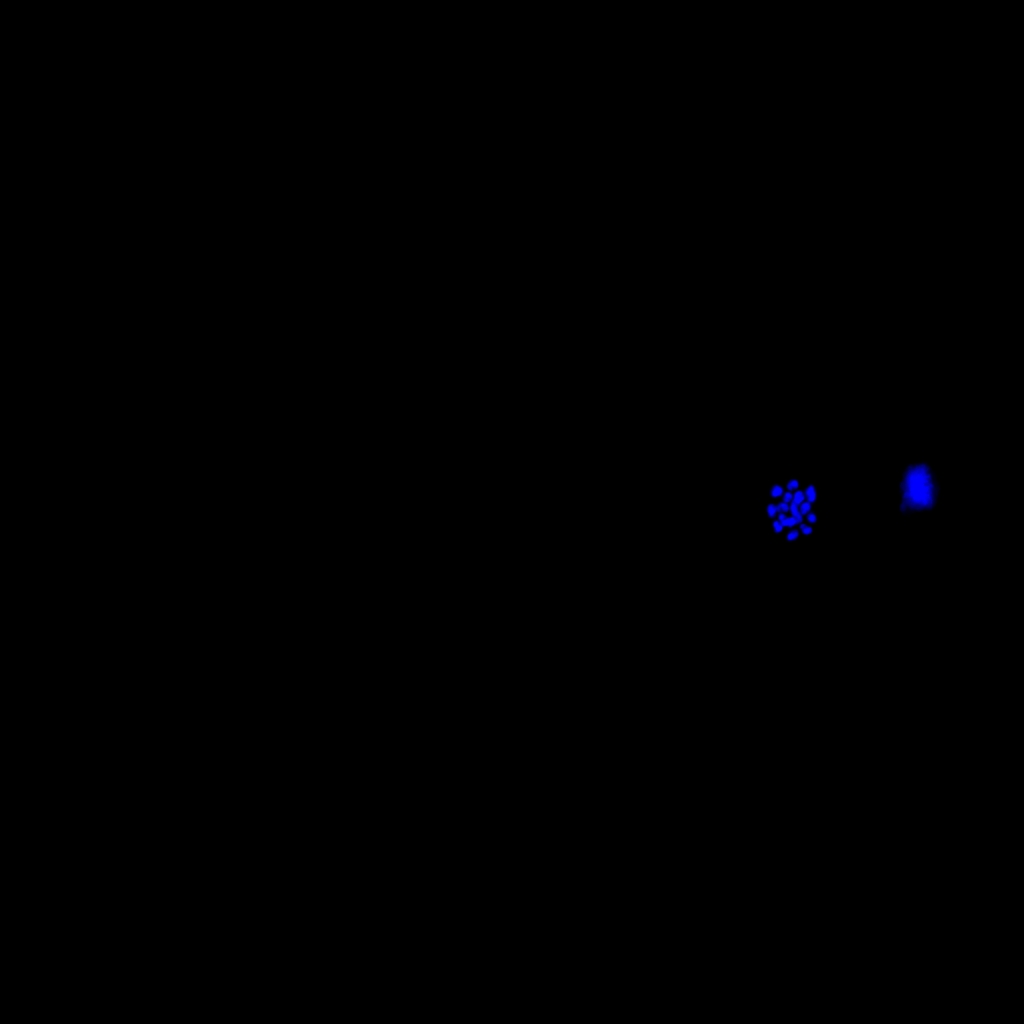

Supplement: S7 Raw images — (ZIP) [file pone.0277477.s009.zip › Fig4d_con_c1.jpg]

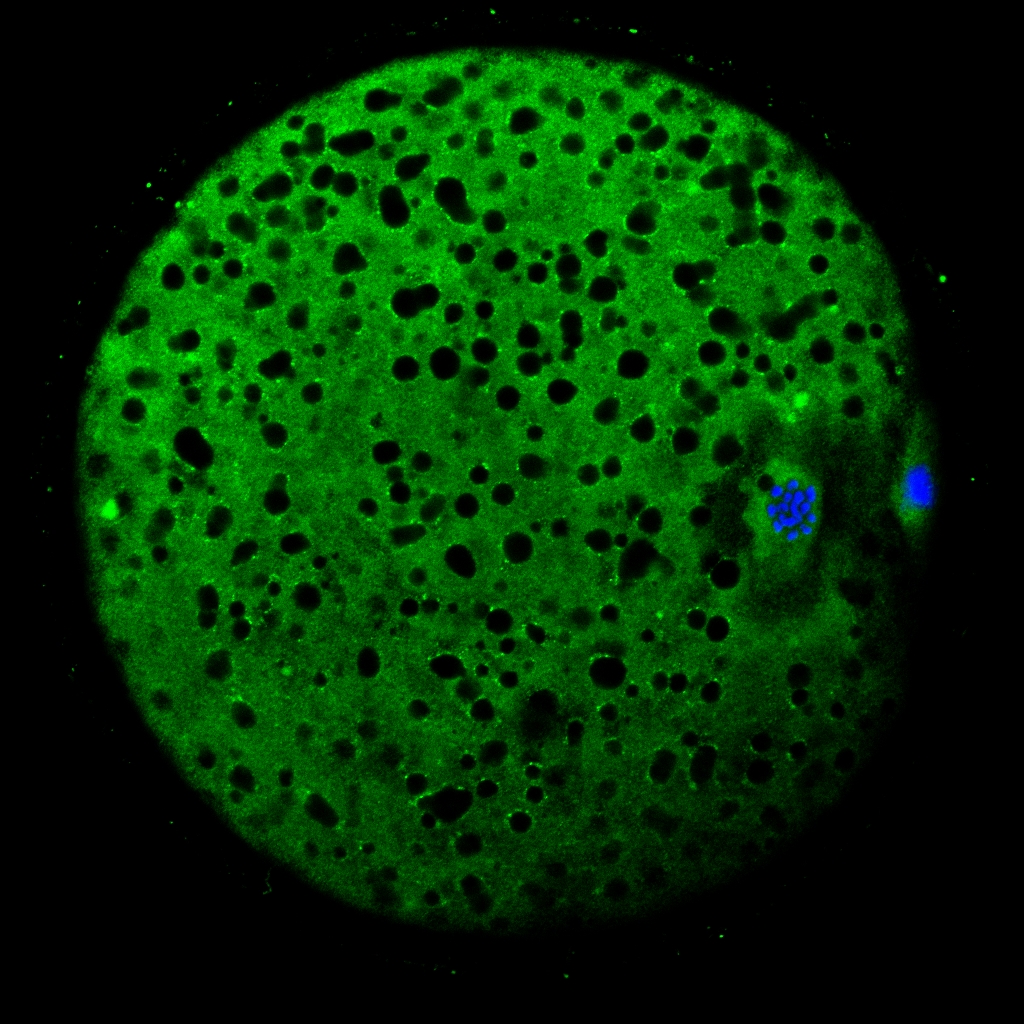

Supplement: S7 Raw images — (ZIP) [file pone.0277477.s009.zip › Fig4d_con_c1+2.jpg]

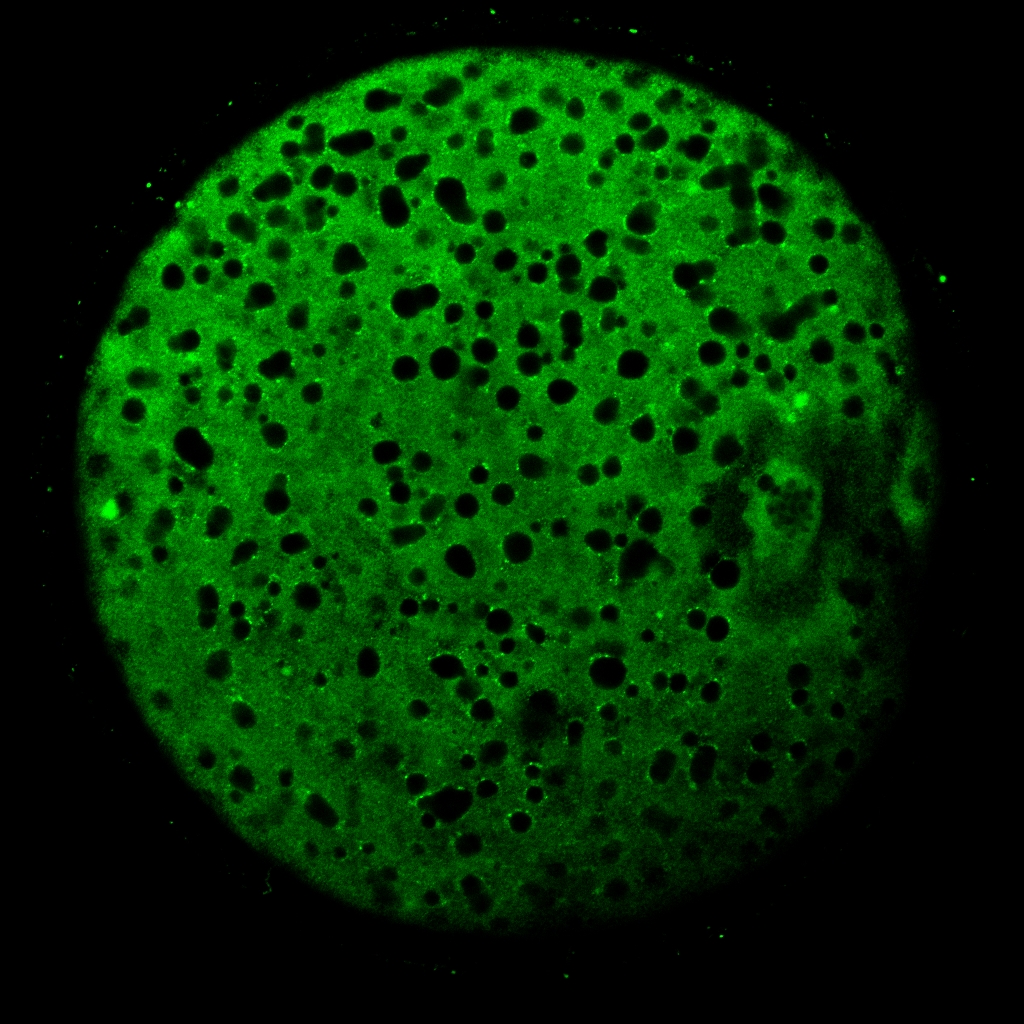

Supplement: S7 Raw images — (ZIP) [file pone.0277477.s009.zip › Fig4d_con_c2.jpg]

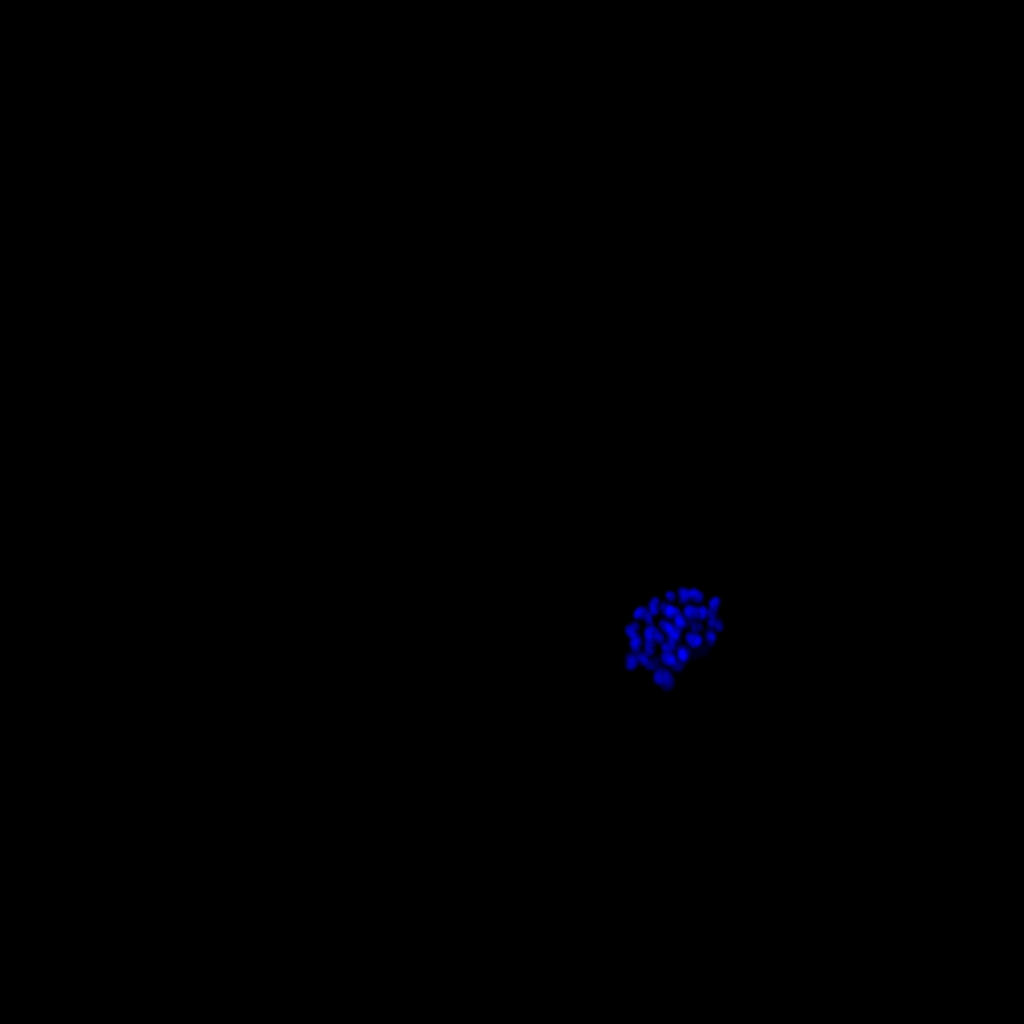

Supplement: S7 Raw images — (ZIP) [file pone.0277477.s009.zip › Fig4d_rot_c1.jpg]

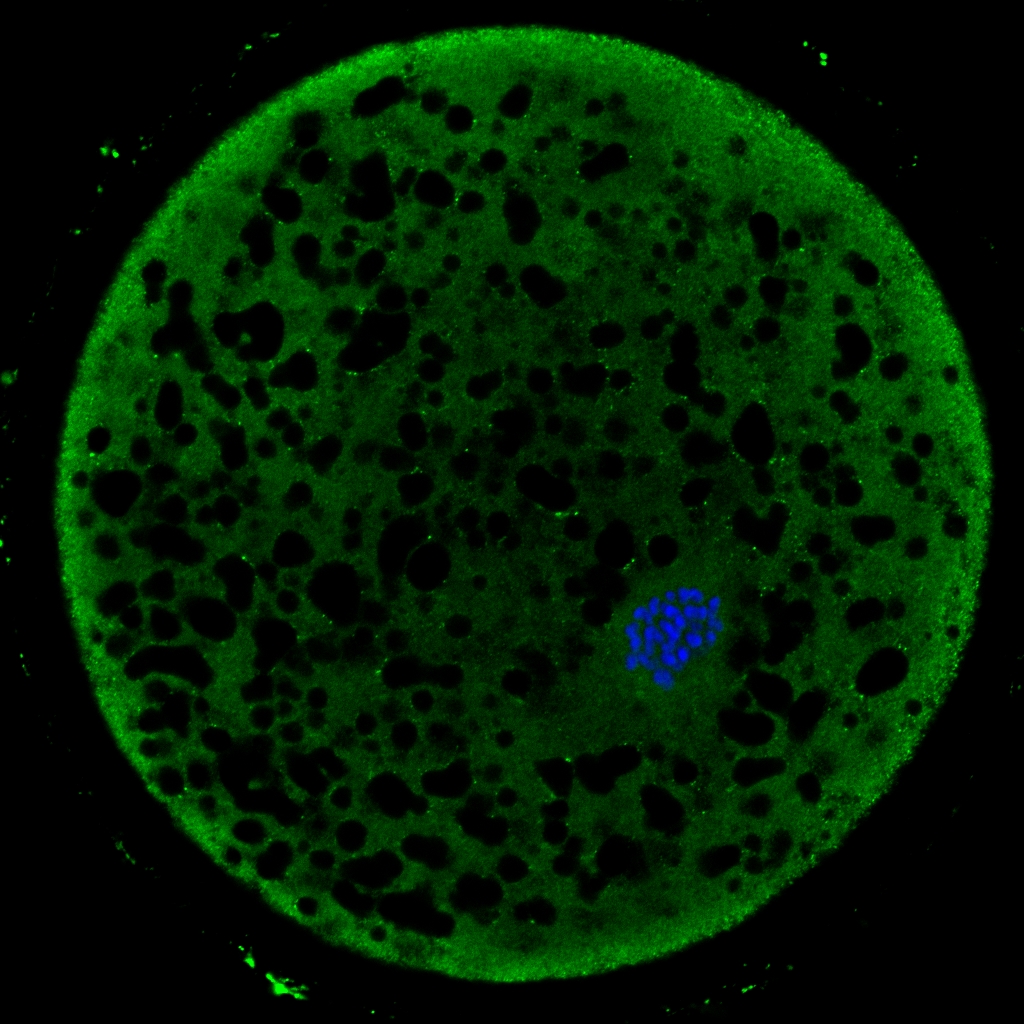

Supplement: S7 Raw images — (ZIP) [file pone.0277477.s009.zip › Fig4d_rot_c1+2.jpg]

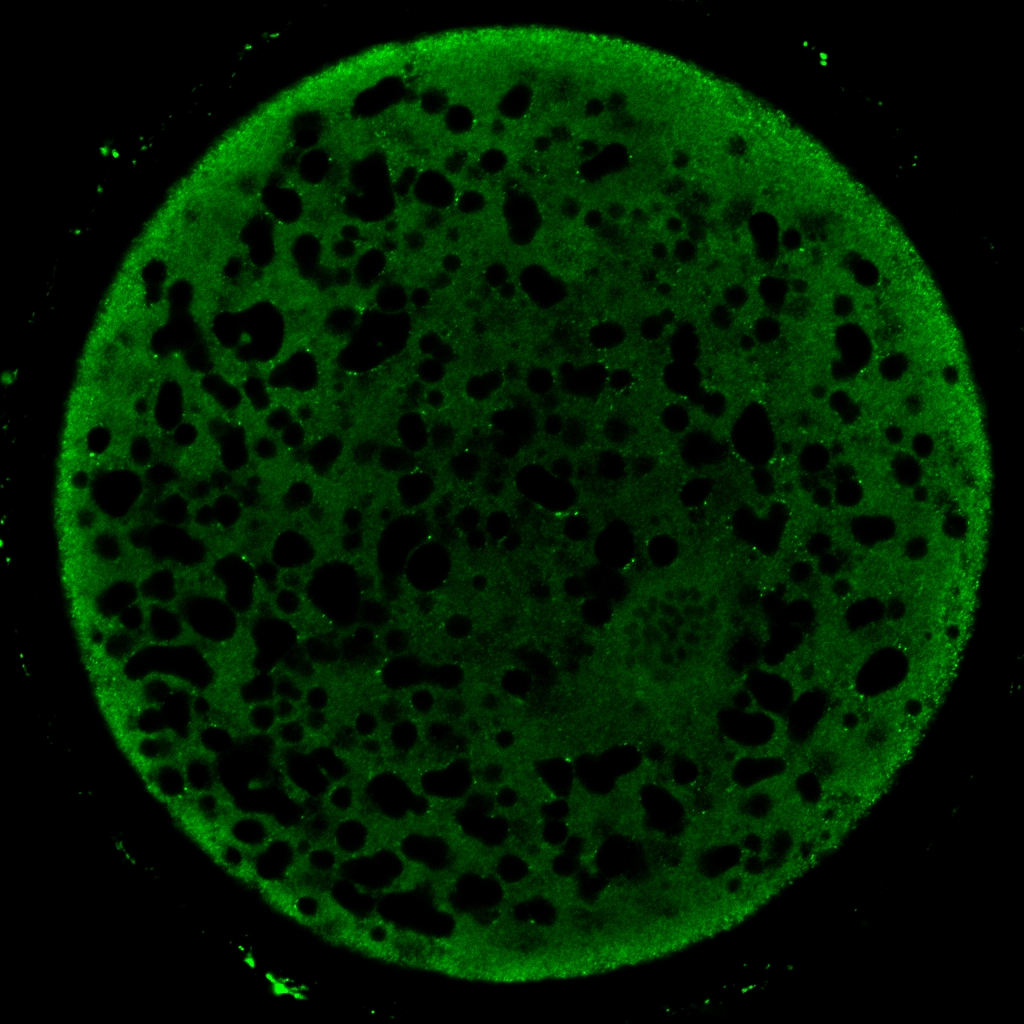

Supplement: S7 Raw images — (ZIP) [file pone.0277477.s009.zip › Fig4d_rot_c2.jpg]

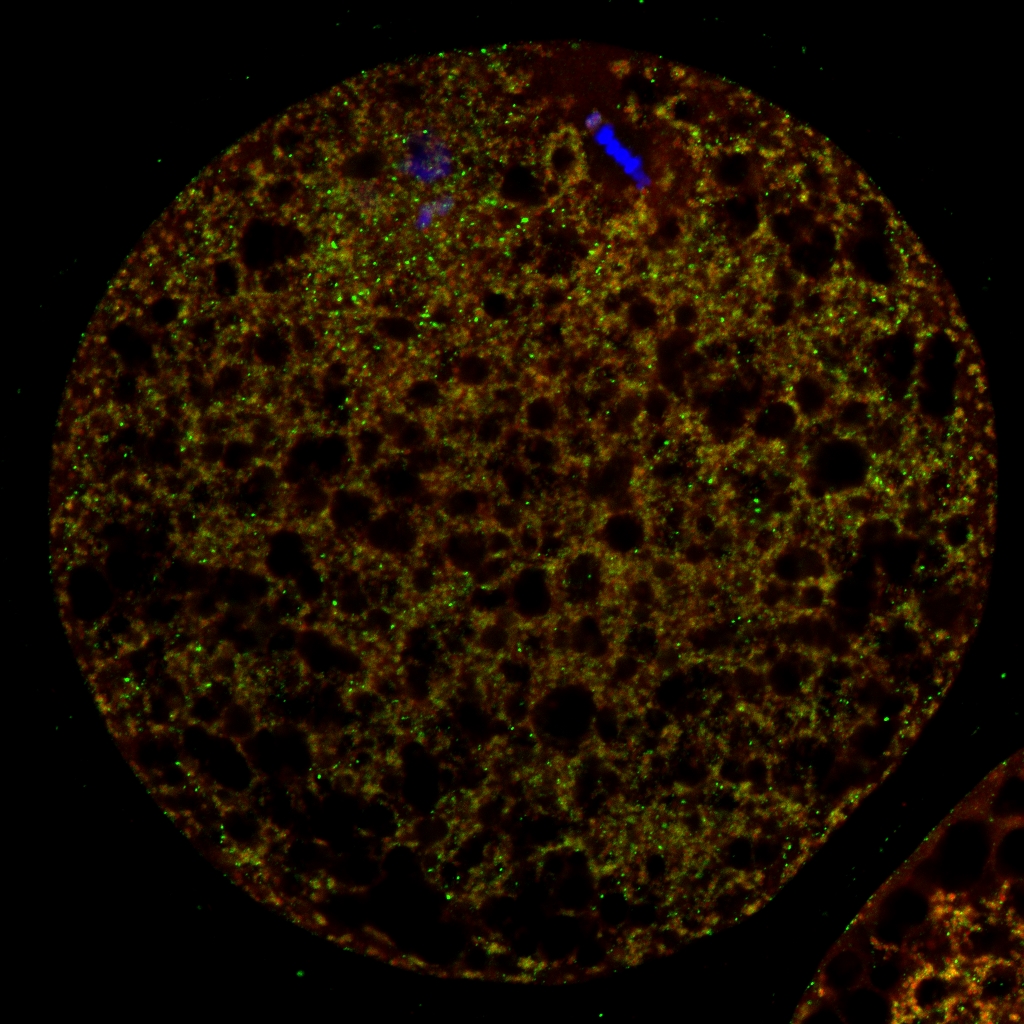

Supplement: S8 Raw images — (ZIP) [file pone.0277477.s010.zip › Fig5a_con_c1+2+3.jpg]

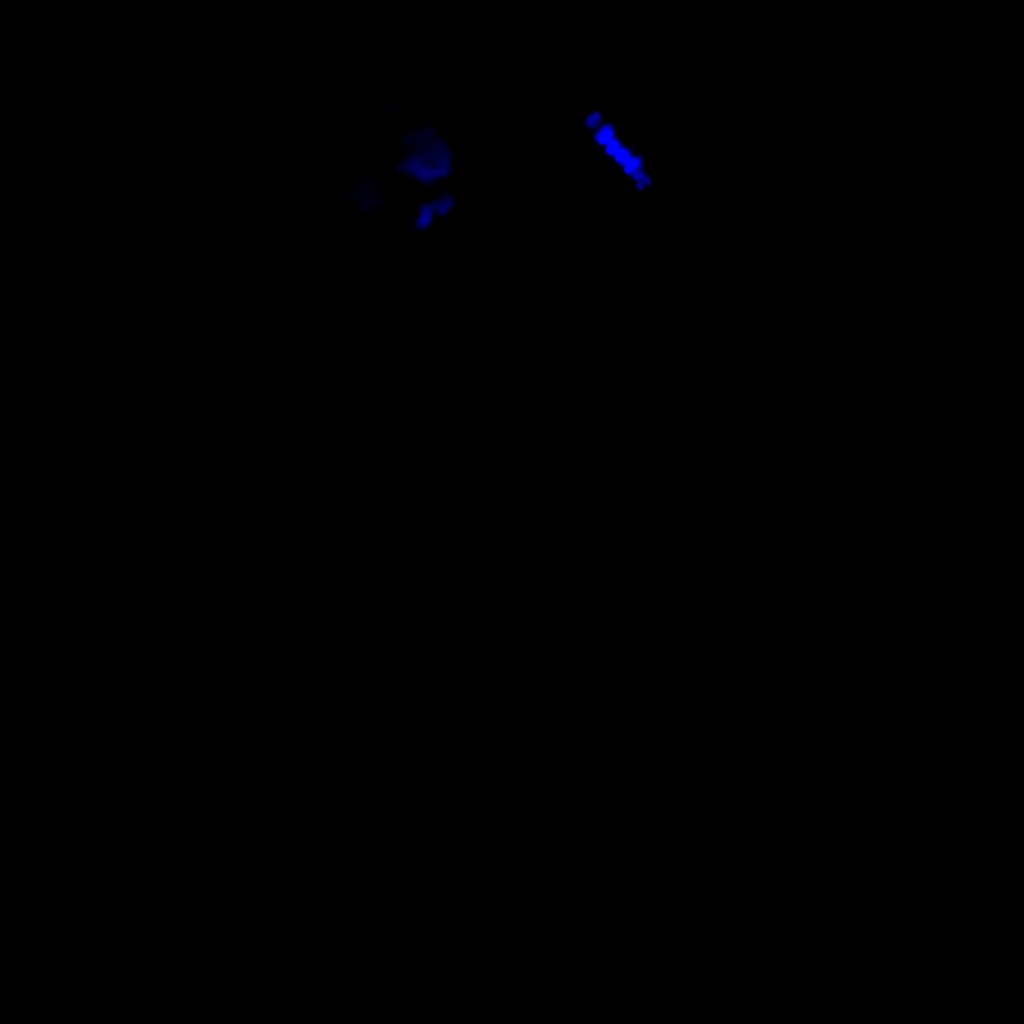

Supplement: S8 Raw images — (ZIP) [file pone.0277477.s010.zip › Fig5a_con_c1.jpg]

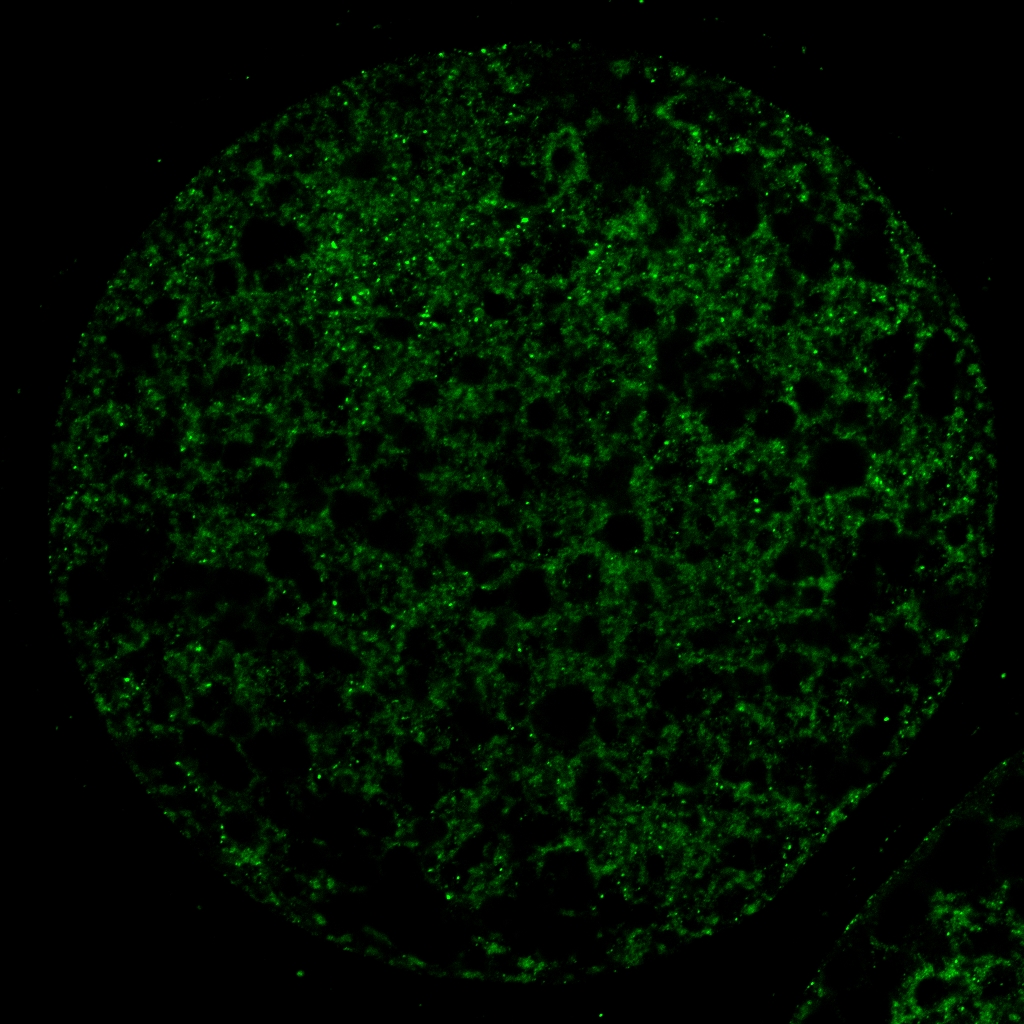

Supplement: S8 Raw images — (ZIP) [file pone.0277477.s010.zip › Fig5a_con_c2.jpg]

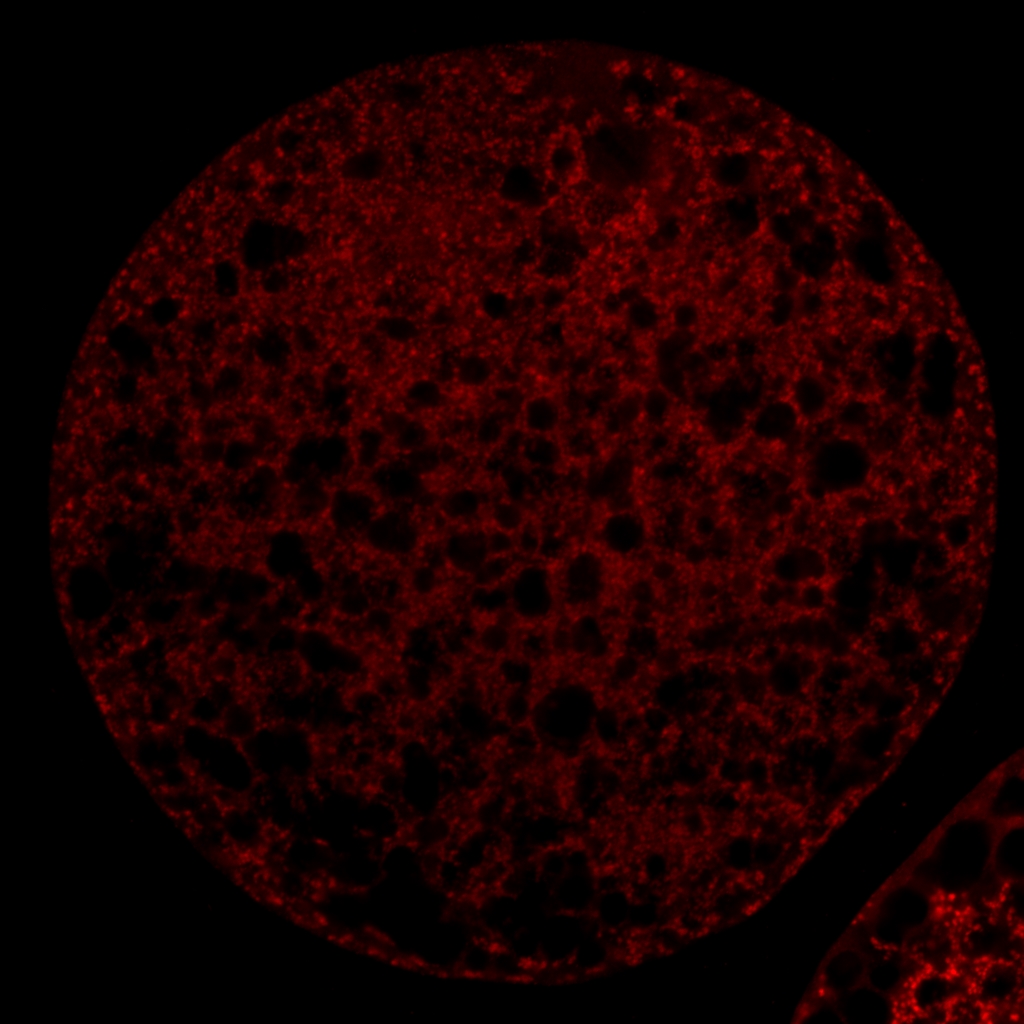

Supplement: S8 Raw images — (ZIP) [file pone.0277477.s010.zip › Fig5a_con_c3.jpg]

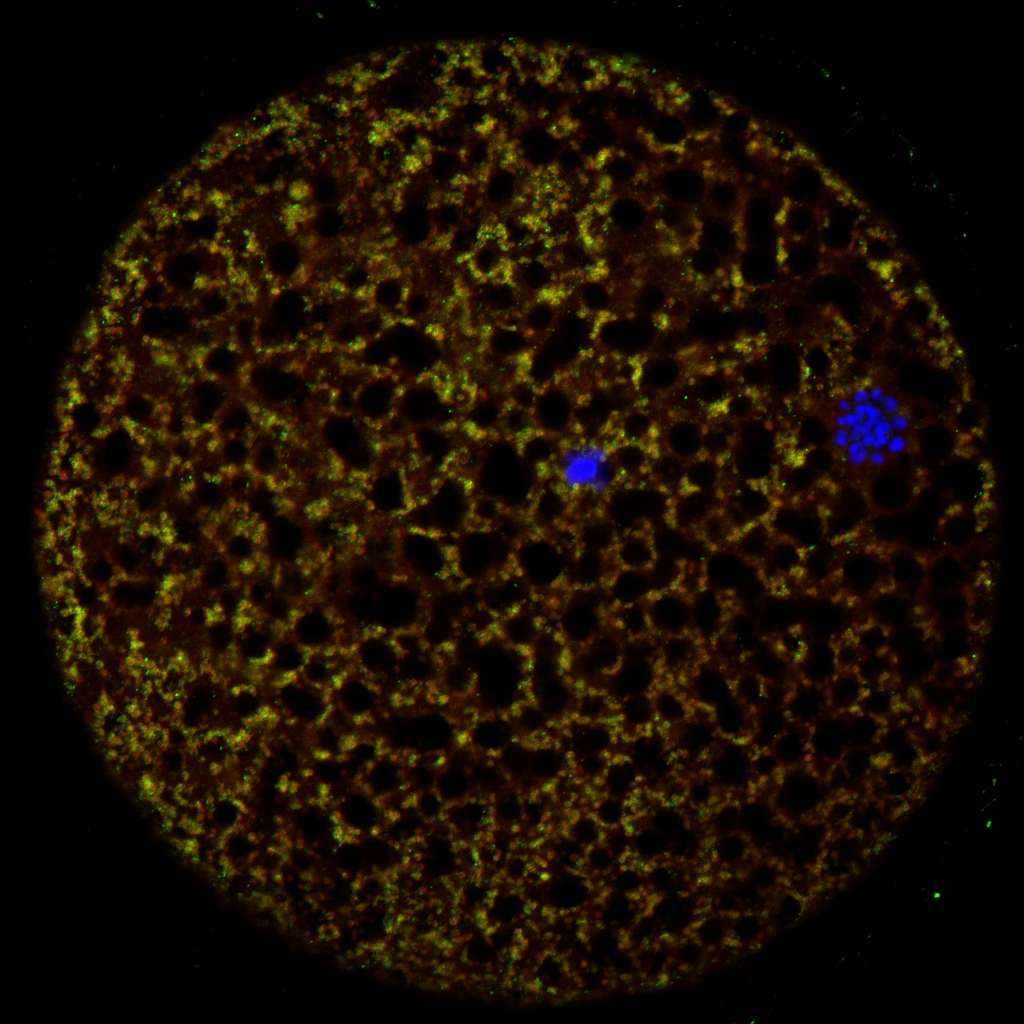

Supplement: S8 Raw images — (ZIP) [file pone.0277477.s010.zip › Fig5a_rot_c1+2+3.jpg]

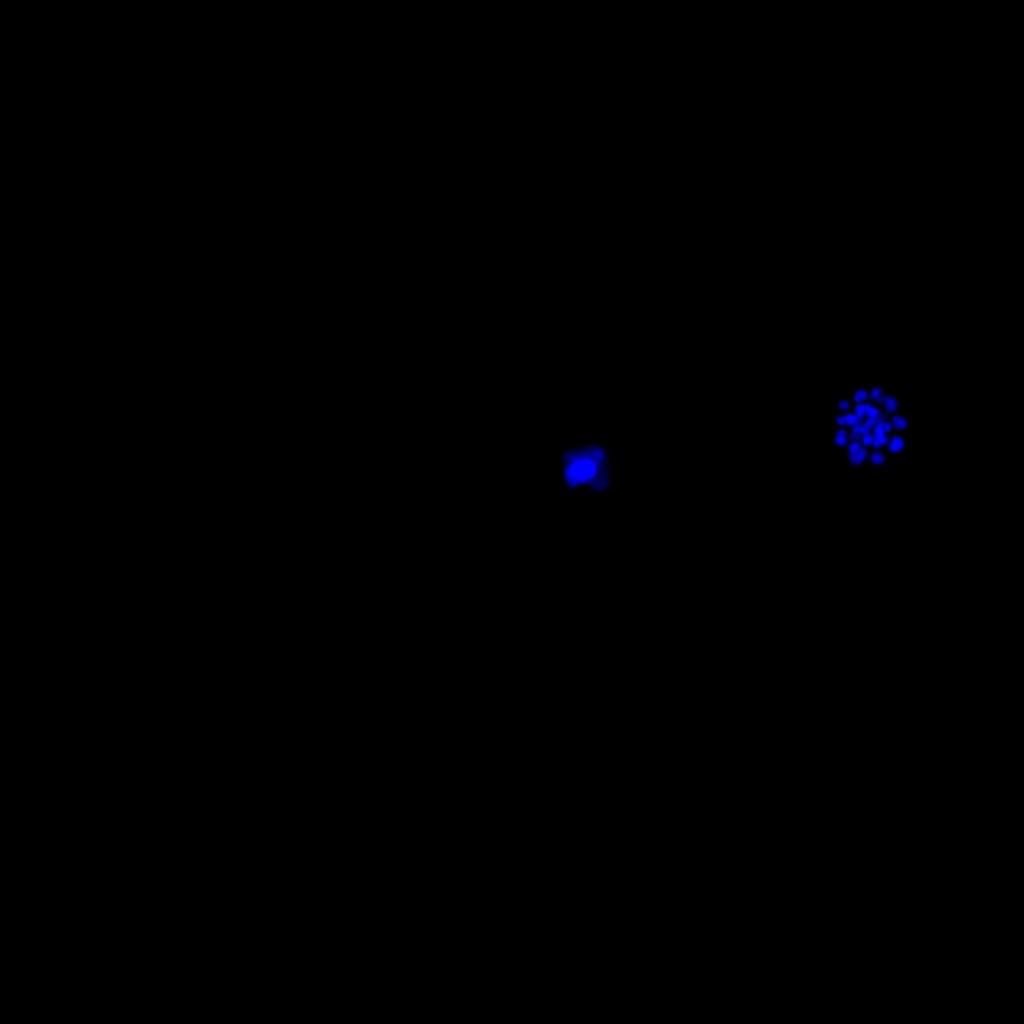

Supplement: S8 Raw images — (ZIP) [file pone.0277477.s010.zip › Fig5a_rot_c1.jpg]

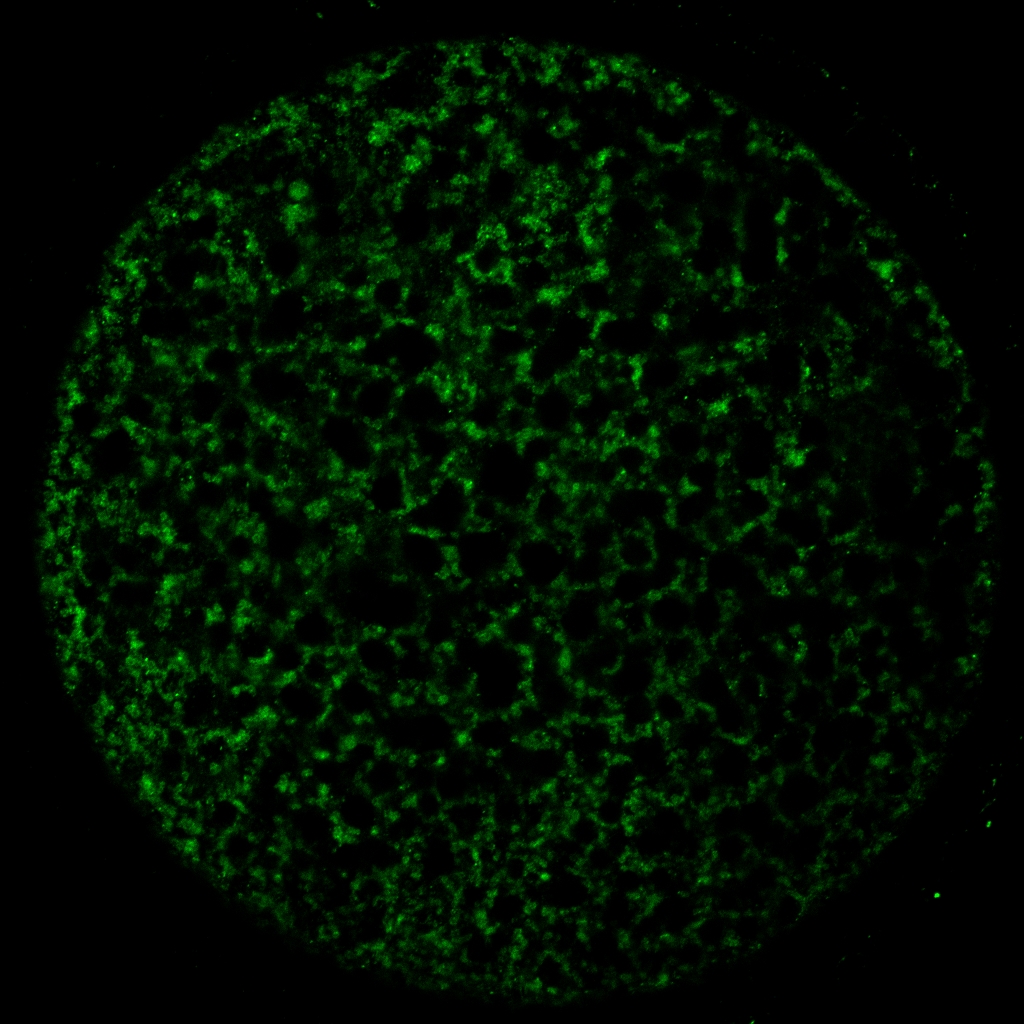

Supplement: S8 Raw images — (ZIP) [file pone.0277477.s010.zip › Fig5a_rot_c2.jpg]

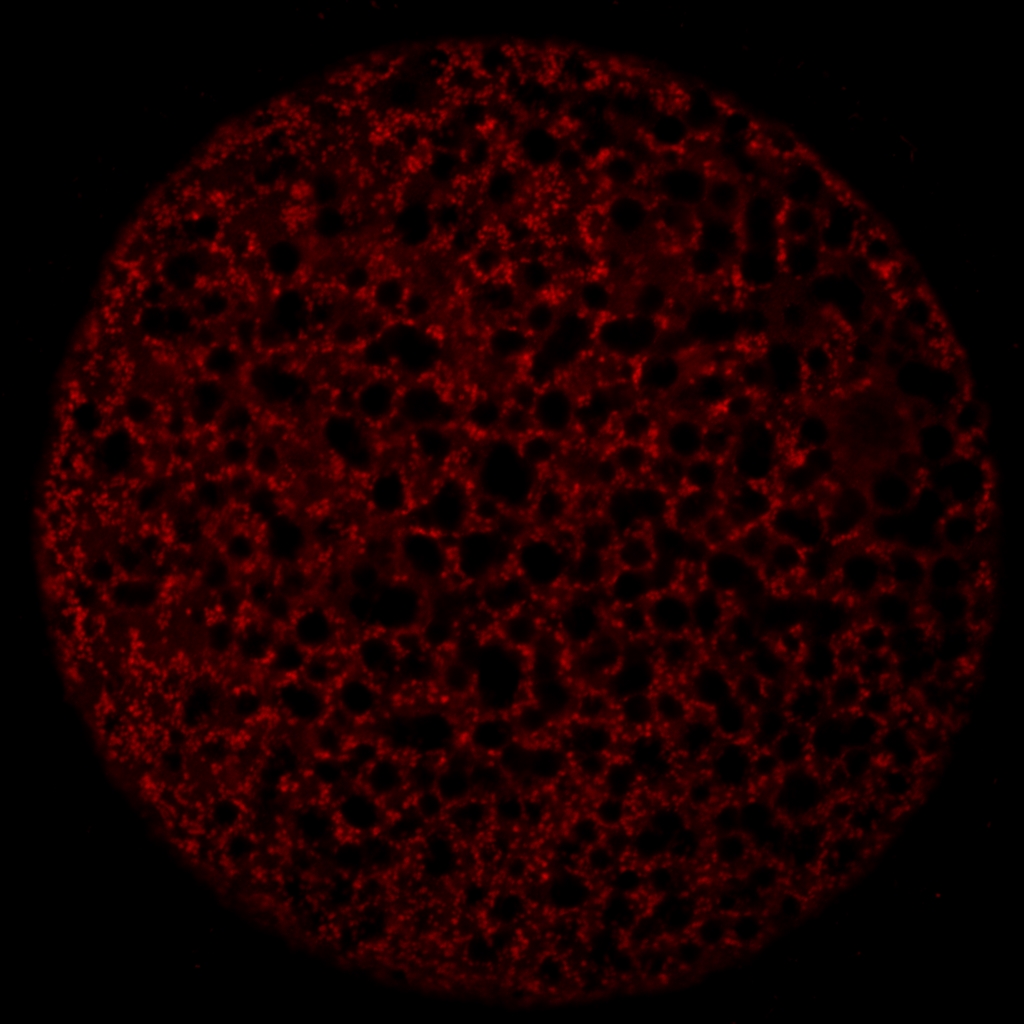

Supplement: S8 Raw images — (ZIP) [file pone.0277477.s010.zip › Fig5a_rot_c3.jpg]

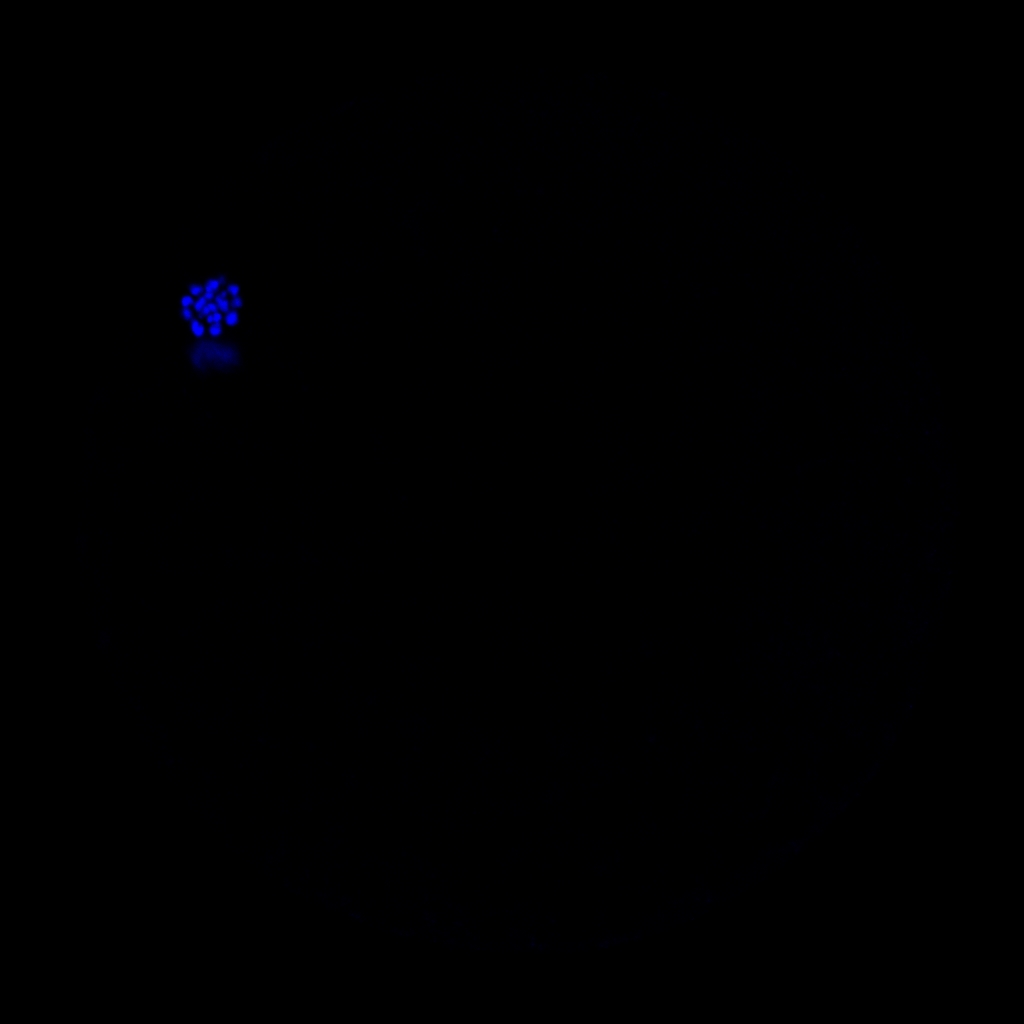

Supplement: S9 Raw images — (ZIP) [file pone.0277477.s011.zip › Fig5e_con_c1.jpg]

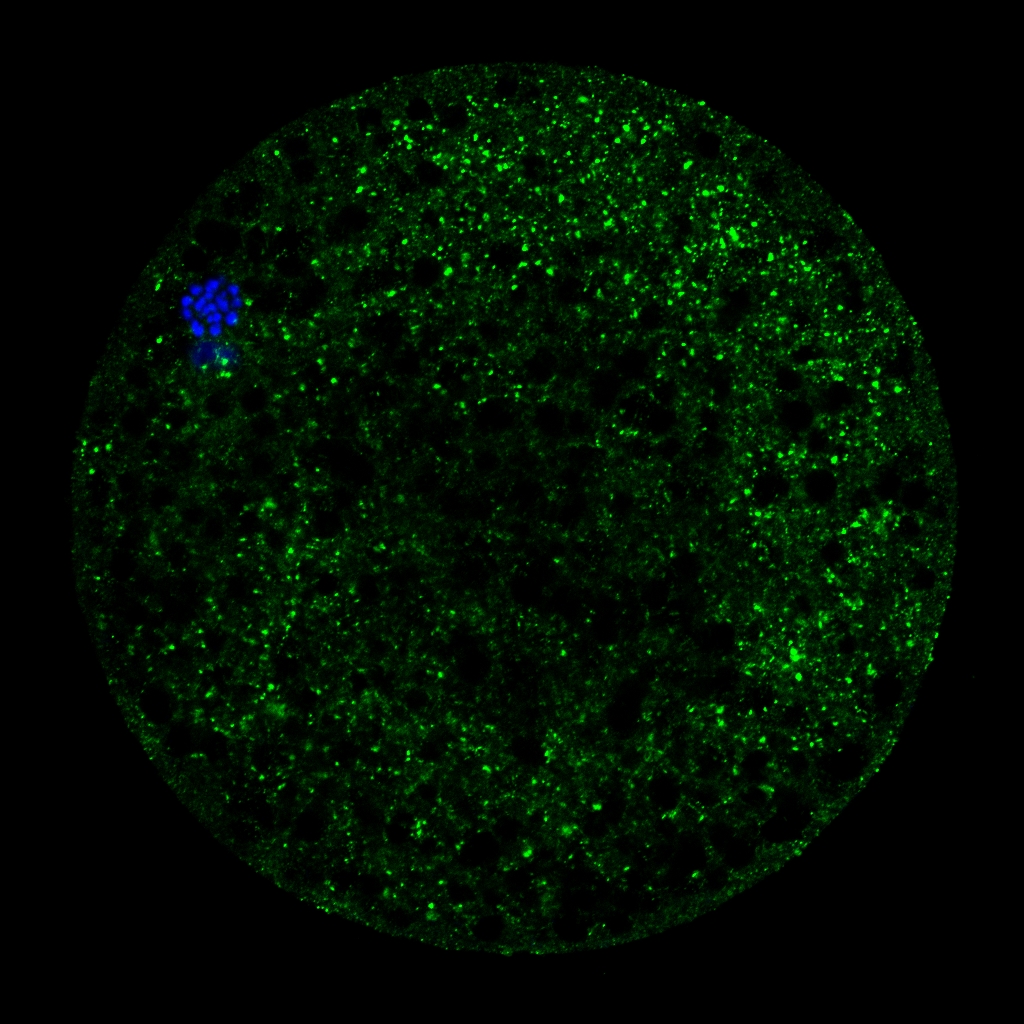

Supplement: S9 Raw images — (ZIP) [file pone.0277477.s011.zip › Fig5e_con_c1+2.jpg]

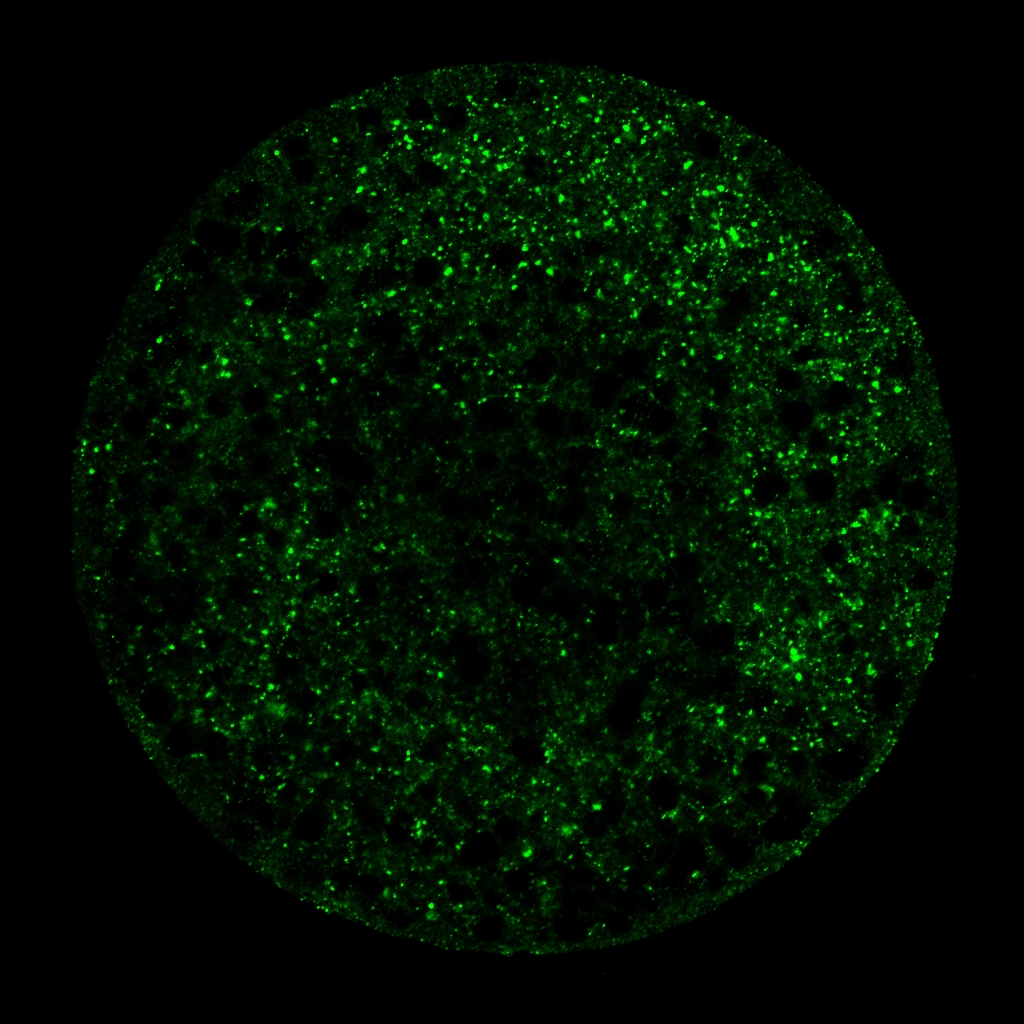

Supplement: S9 Raw images — (ZIP) [file pone.0277477.s011.zip › Fig5e_con_c2.jpg]

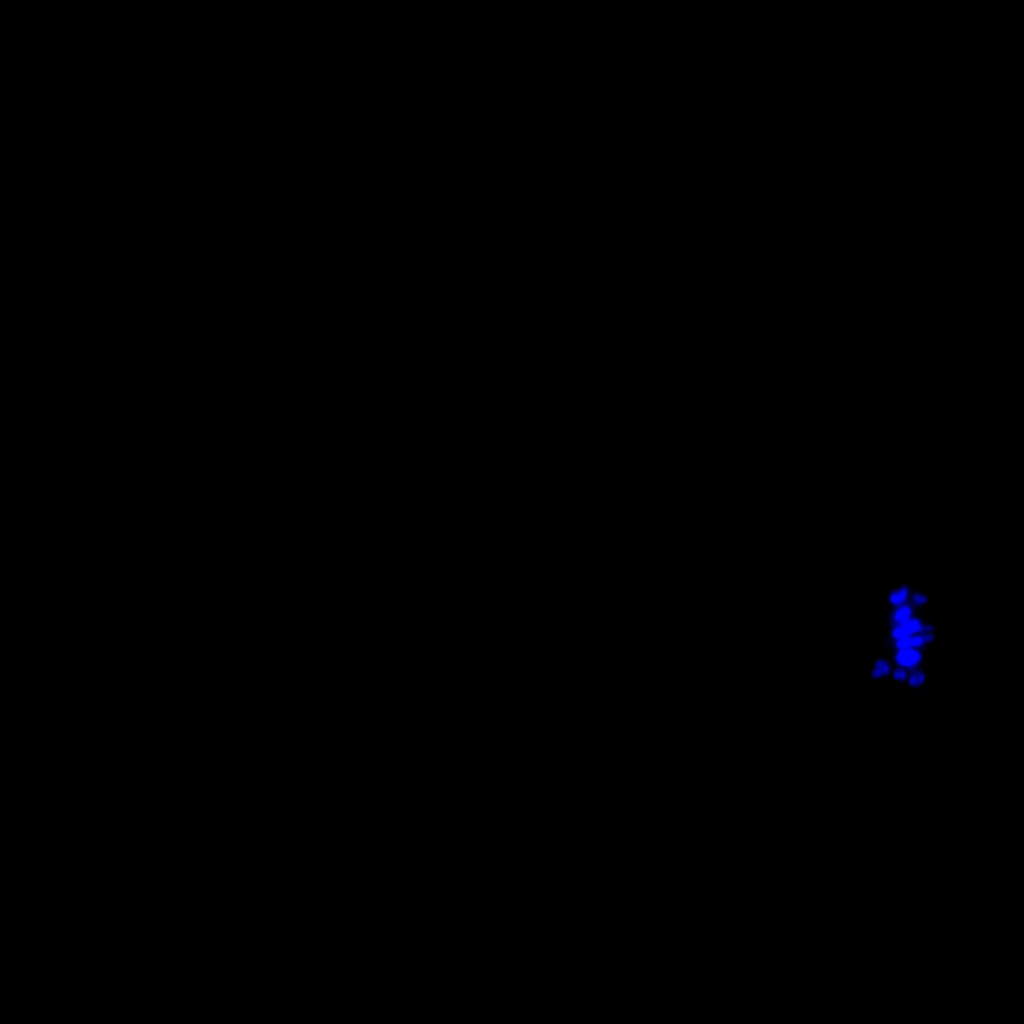

Supplement: S9 Raw images — (ZIP) [file pone.0277477.s011.zip › Fig5e_rot_c1.jpg]

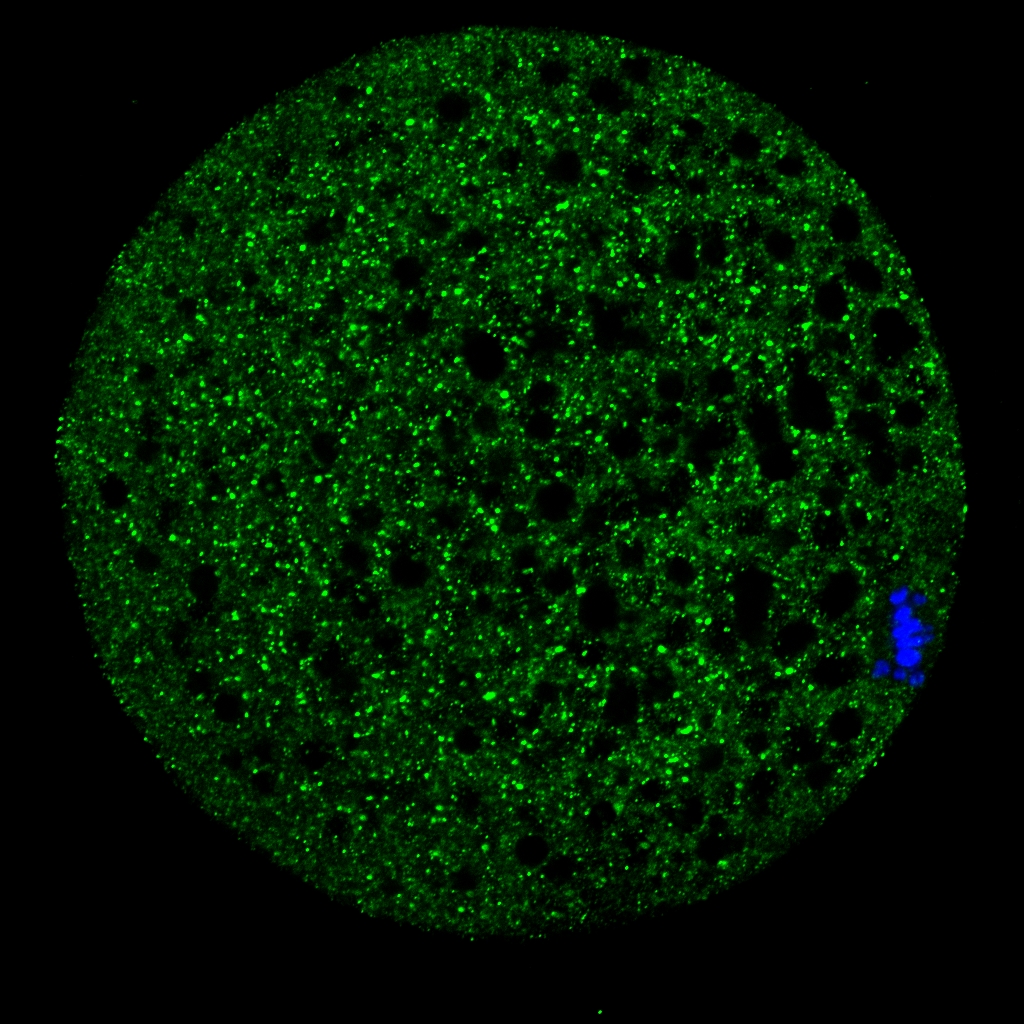

Supplement: S9 Raw images — (ZIP) [file pone.0277477.s011.zip › Fig5e_rot_c1+2.jpg]

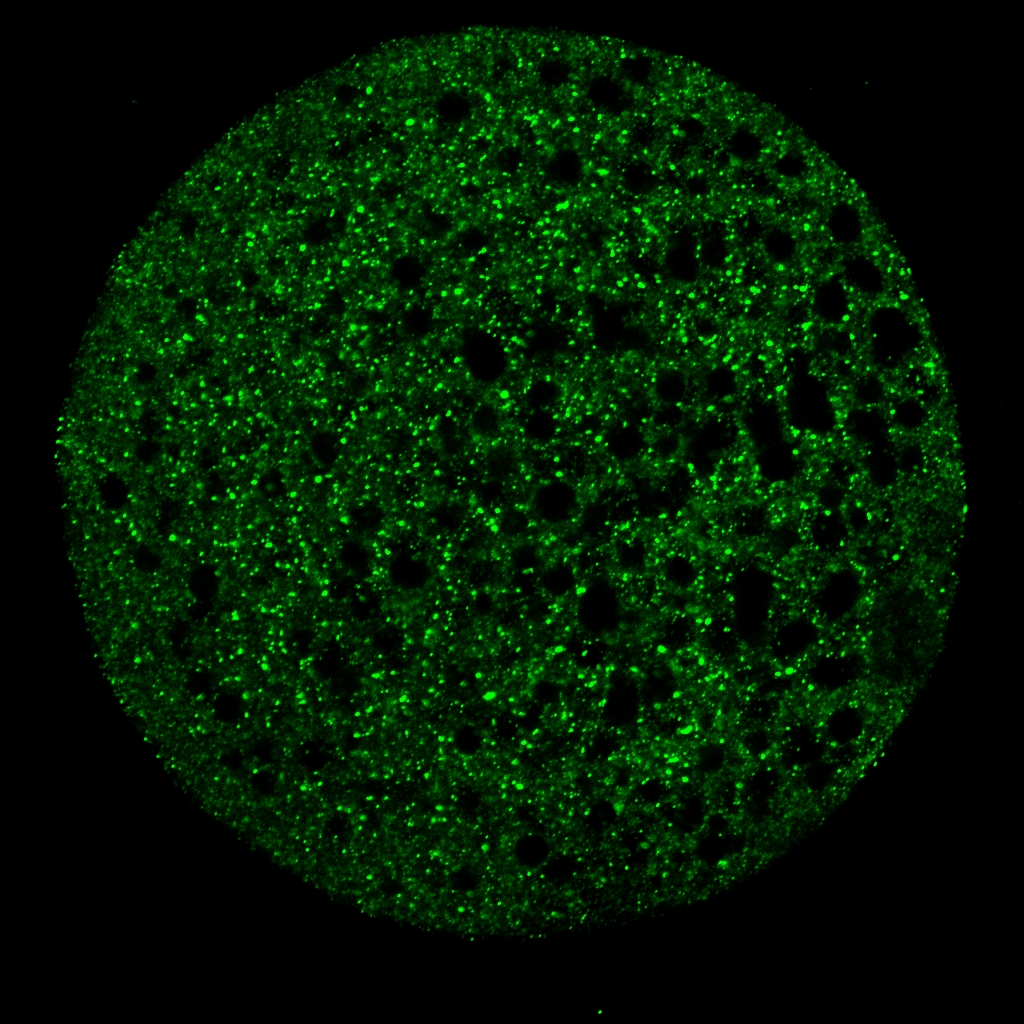

Supplement: S9 Raw images — (ZIP) [file pone.0277477.s011.zip › Fig5e_rot_c2.jpg]

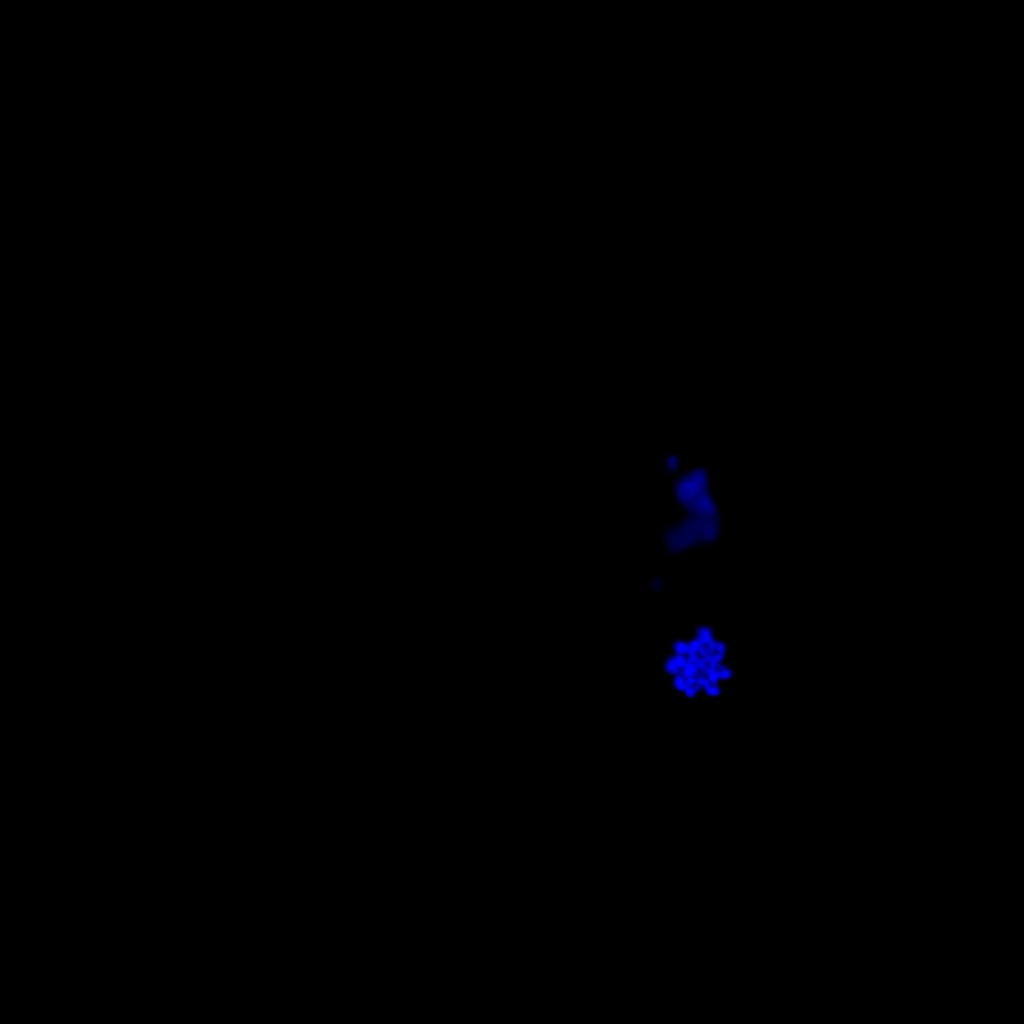

Supplement: S10 Raw images — (ZIP) [file pone.0277477.s012.zip › Fig5g_con_c1.jpg]

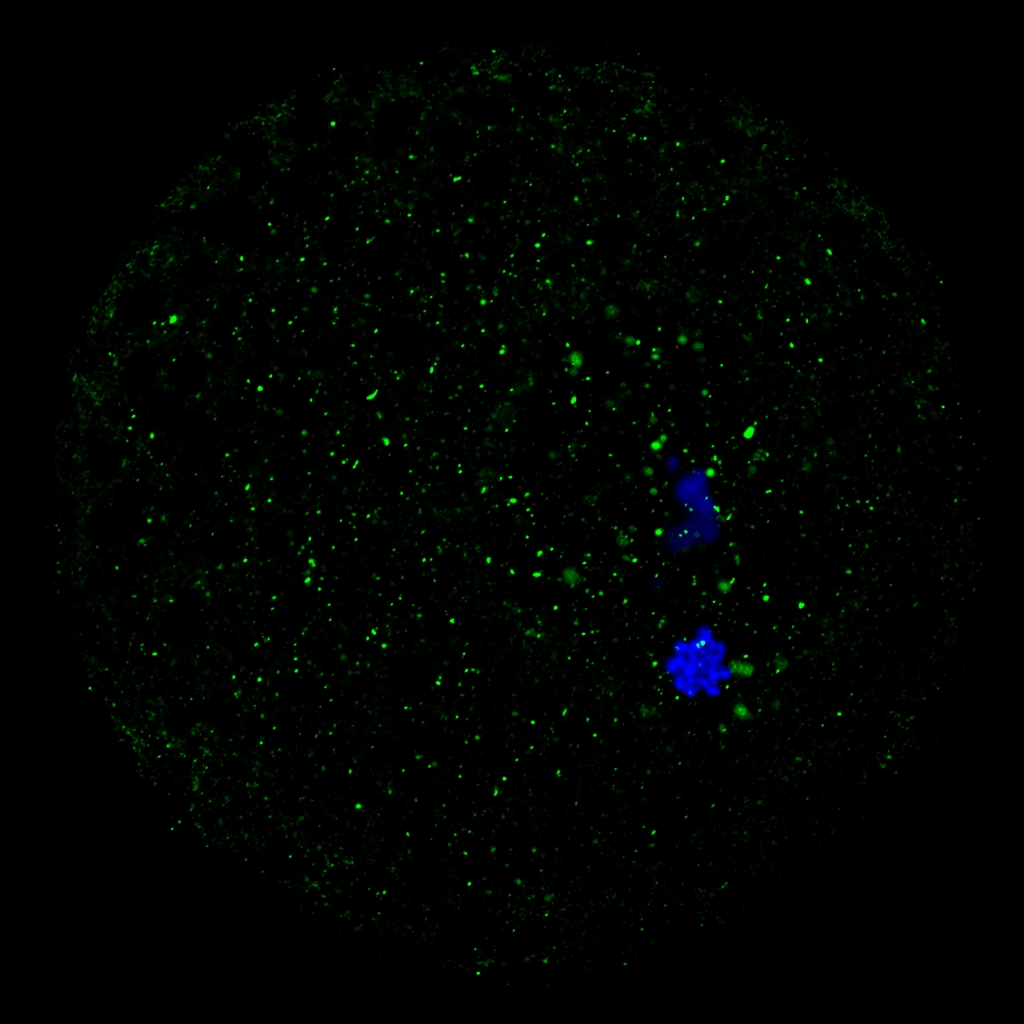

Supplement: S10 Raw images — (ZIP) [file pone.0277477.s012.zip › Fig5g_con_c1+2.jpg]

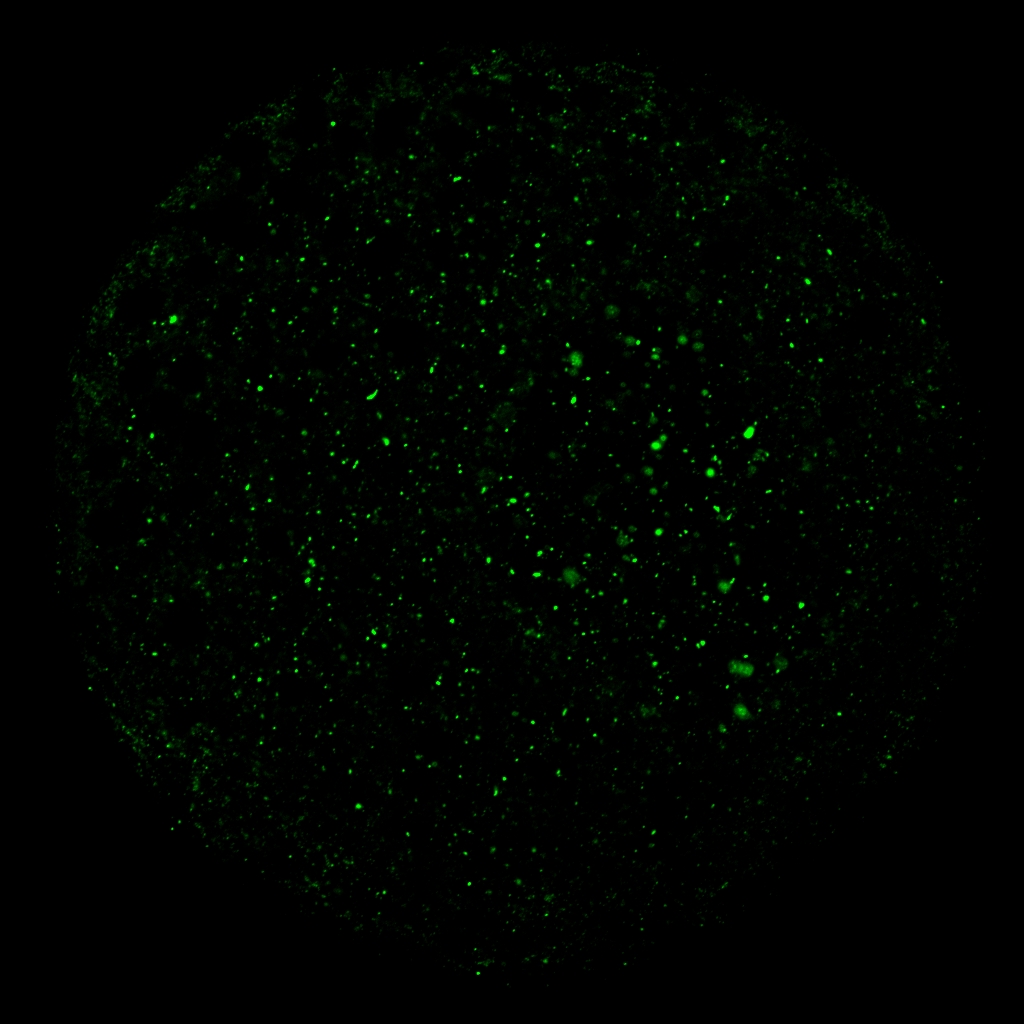

Supplement: S10 Raw images — (ZIP) [file pone.0277477.s012.zip › Fig5g_con_c2.jpg]

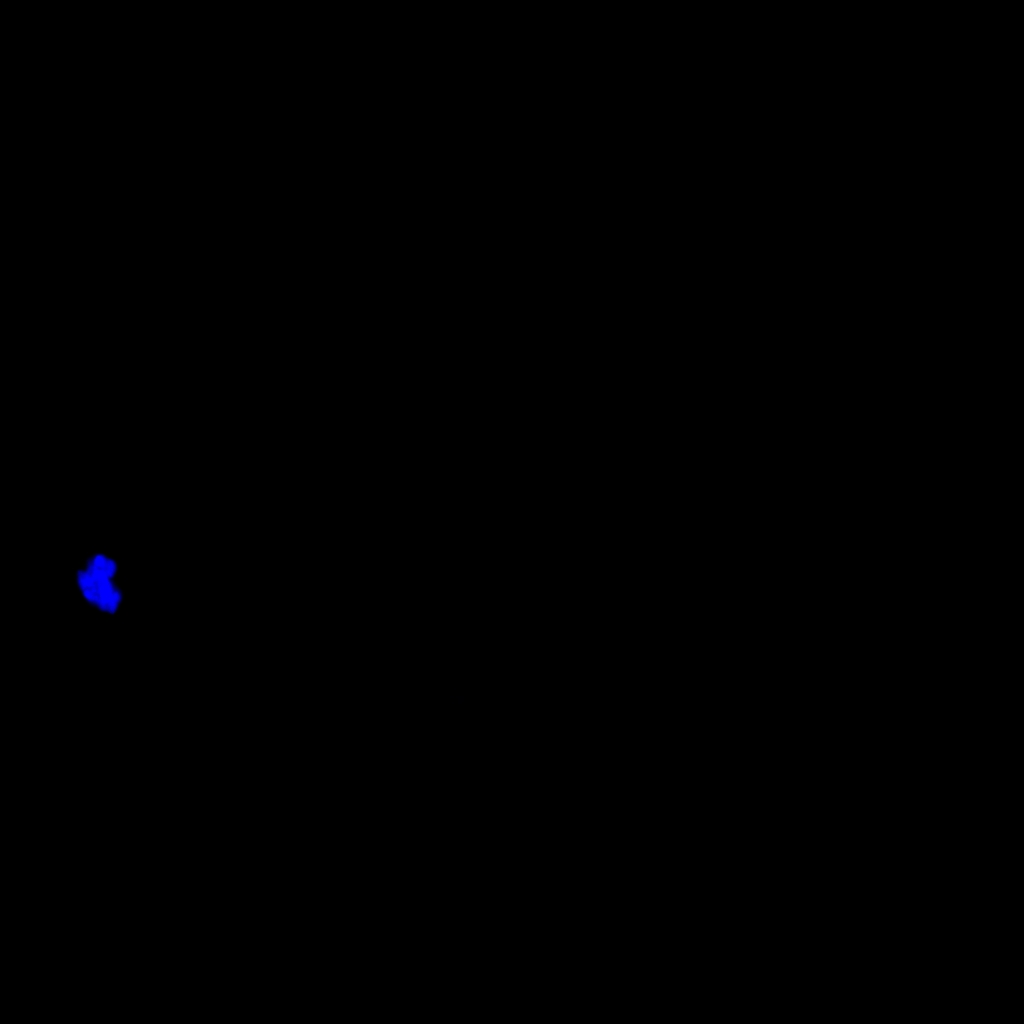

Supplement: S10 Raw images — (ZIP) [file pone.0277477.s012.zip › Fig5g_rot_c1.jpg]

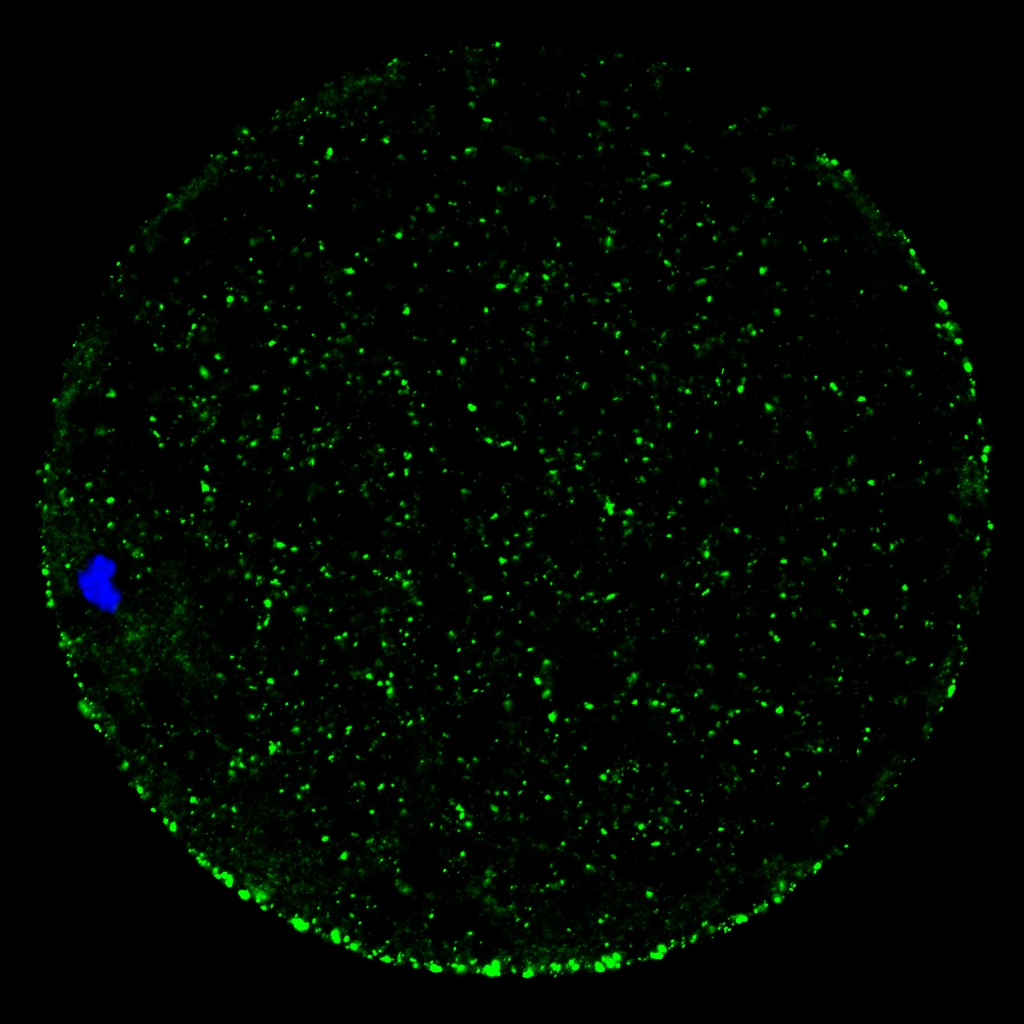

Supplement: S10 Raw images — (ZIP) [file pone.0277477.s012.zip › Fig5g_rot_c1+2.jpg]

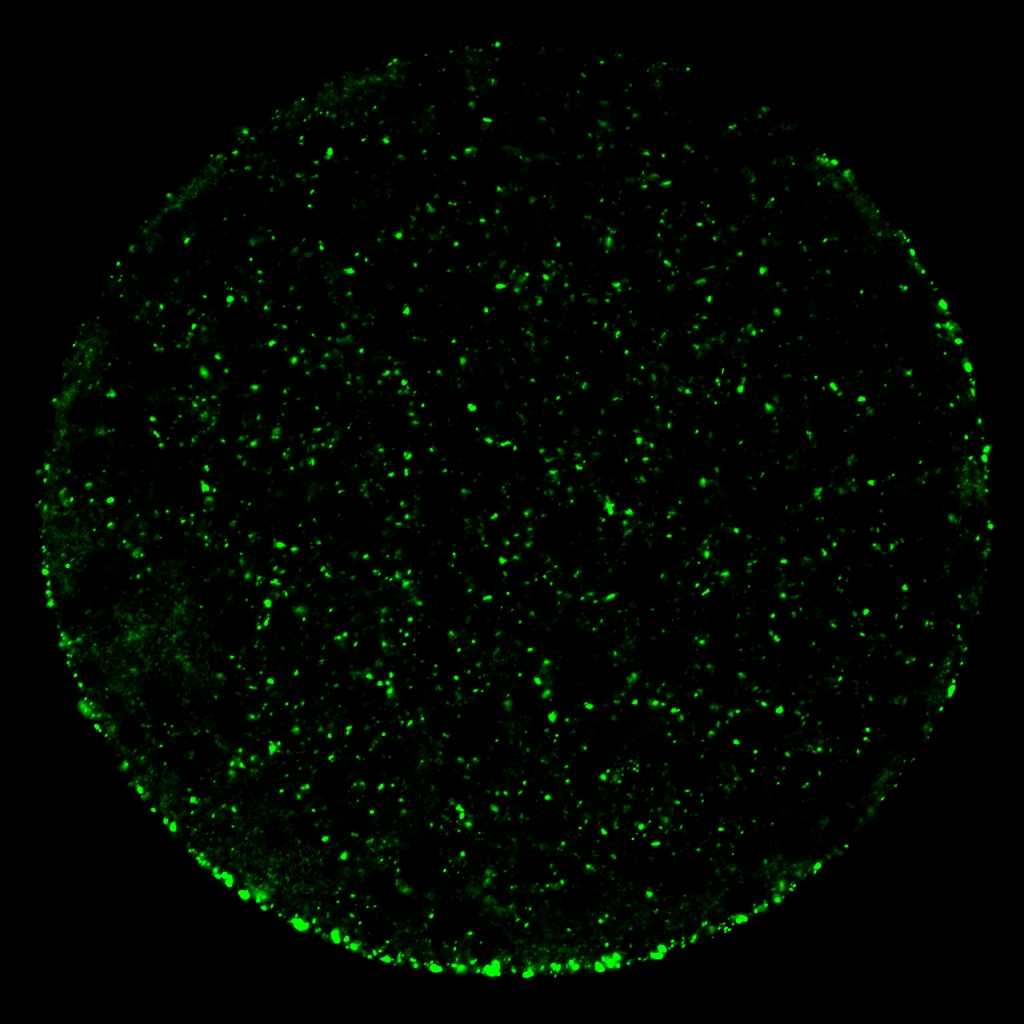

Supplement: S10 Raw images — (ZIP) [file pone.0277477.s012.zip › Fig5g_rot_c2.jpg]

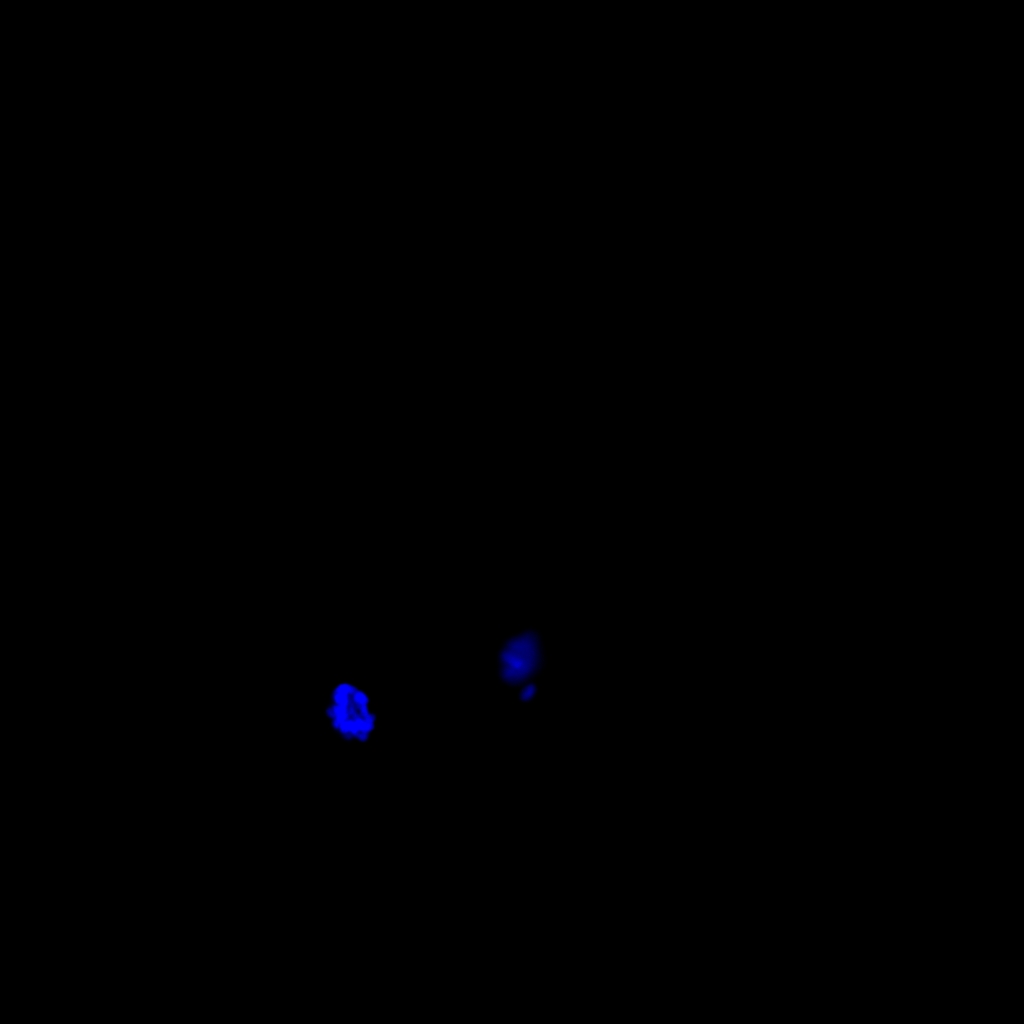

Supplement: S11 Raw images — (ZIP) [file pone.0277477.s013.zip › Fig6a_con_c1.jpg]

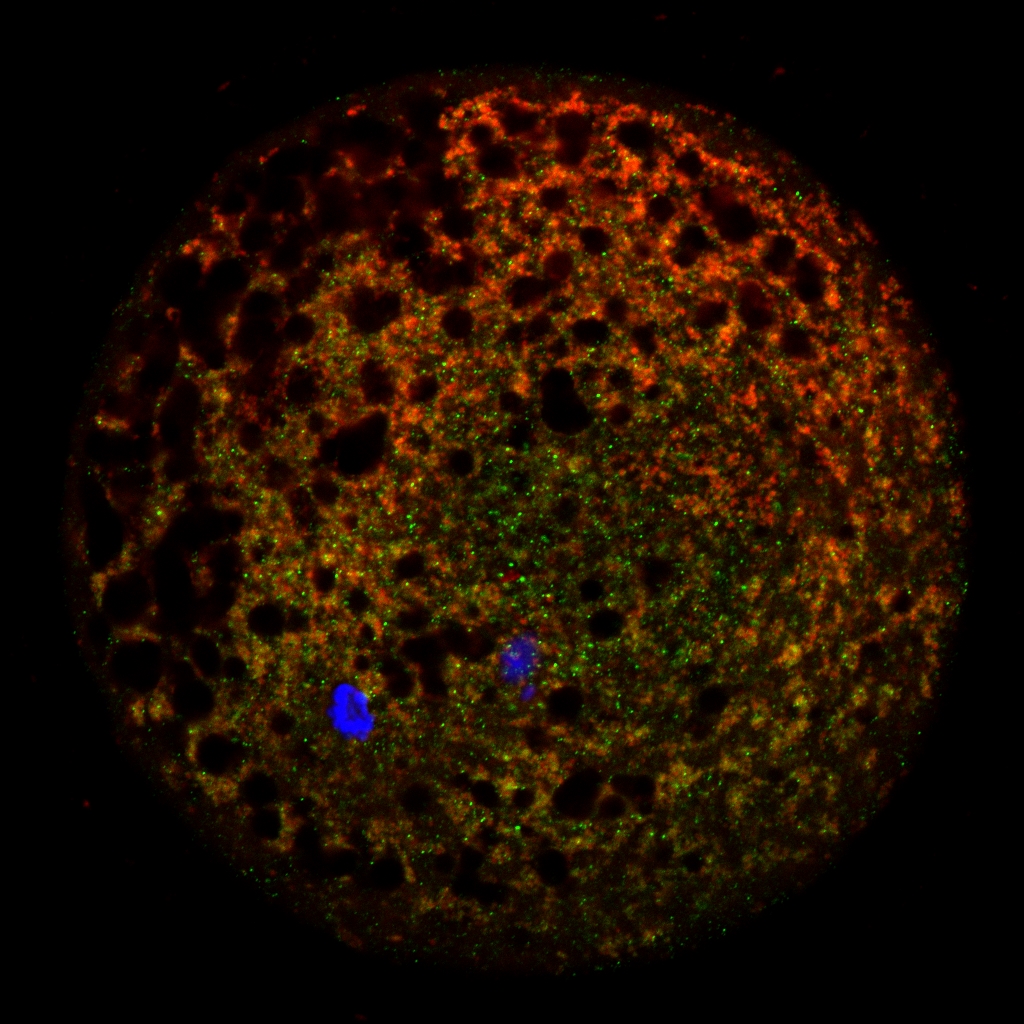

Supplement: S11 Raw images — (ZIP) [file pone.0277477.s013.zip › Fig6a_con_c1+2+3.jpg]

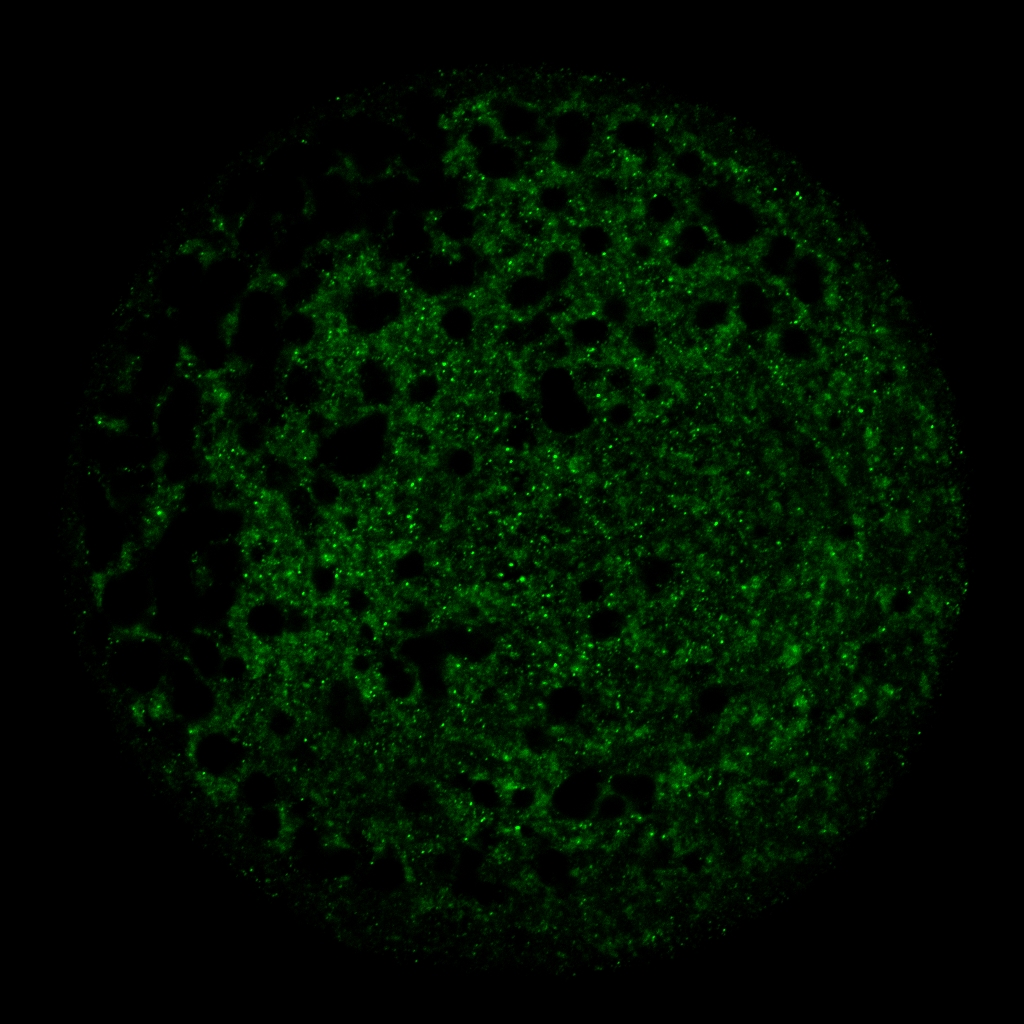

Supplement: S11 Raw images — (ZIP) [file pone.0277477.s013.zip › Fig6a_con_c2.jpg]

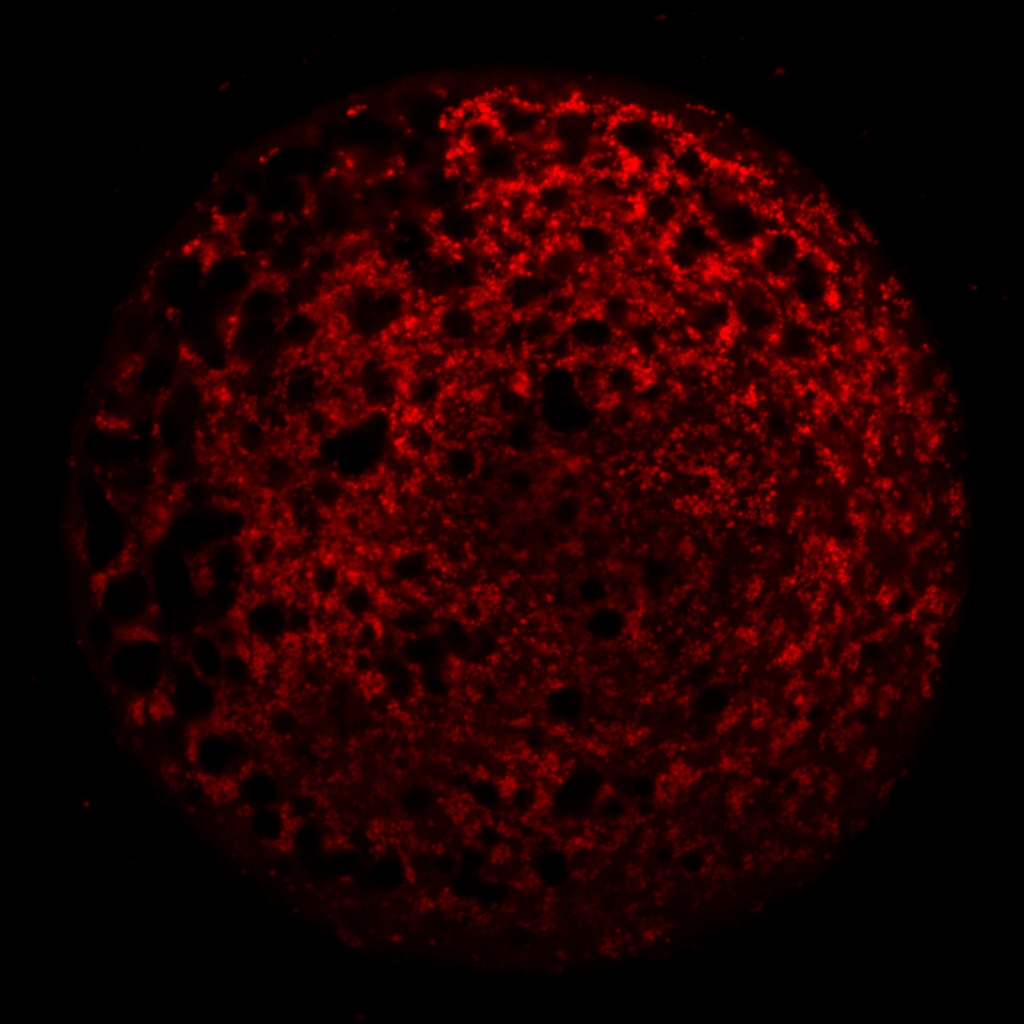

Supplement: S11 Raw images — (ZIP) [file pone.0277477.s013.zip › Fig6a_con_c3.jpg]

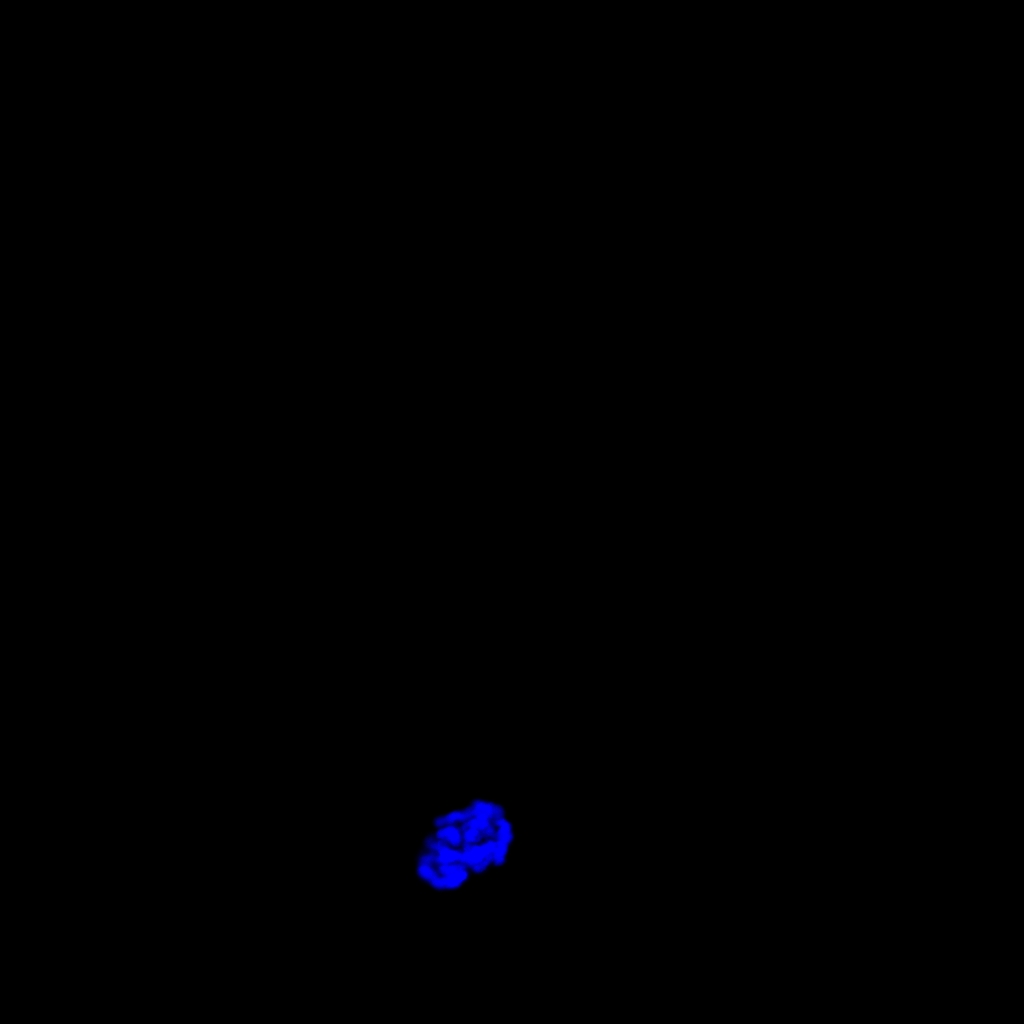

Supplement: S11 Raw images — (ZIP) [file pone.0277477.s013.zip › Fig6a_rot_c1.jpg]

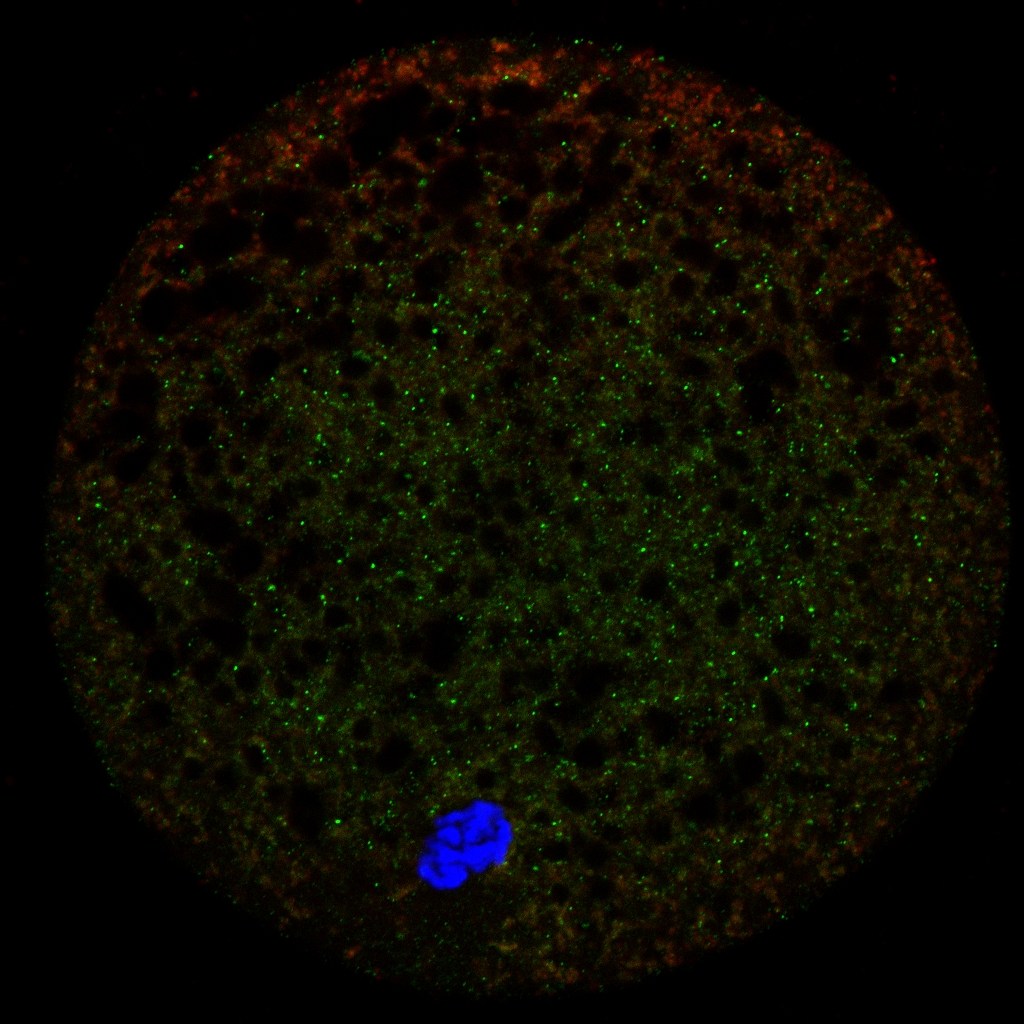

Supplement: S11 Raw images — (ZIP) [file pone.0277477.s013.zip › Fig6a_rot_c1+2+3.jpg]

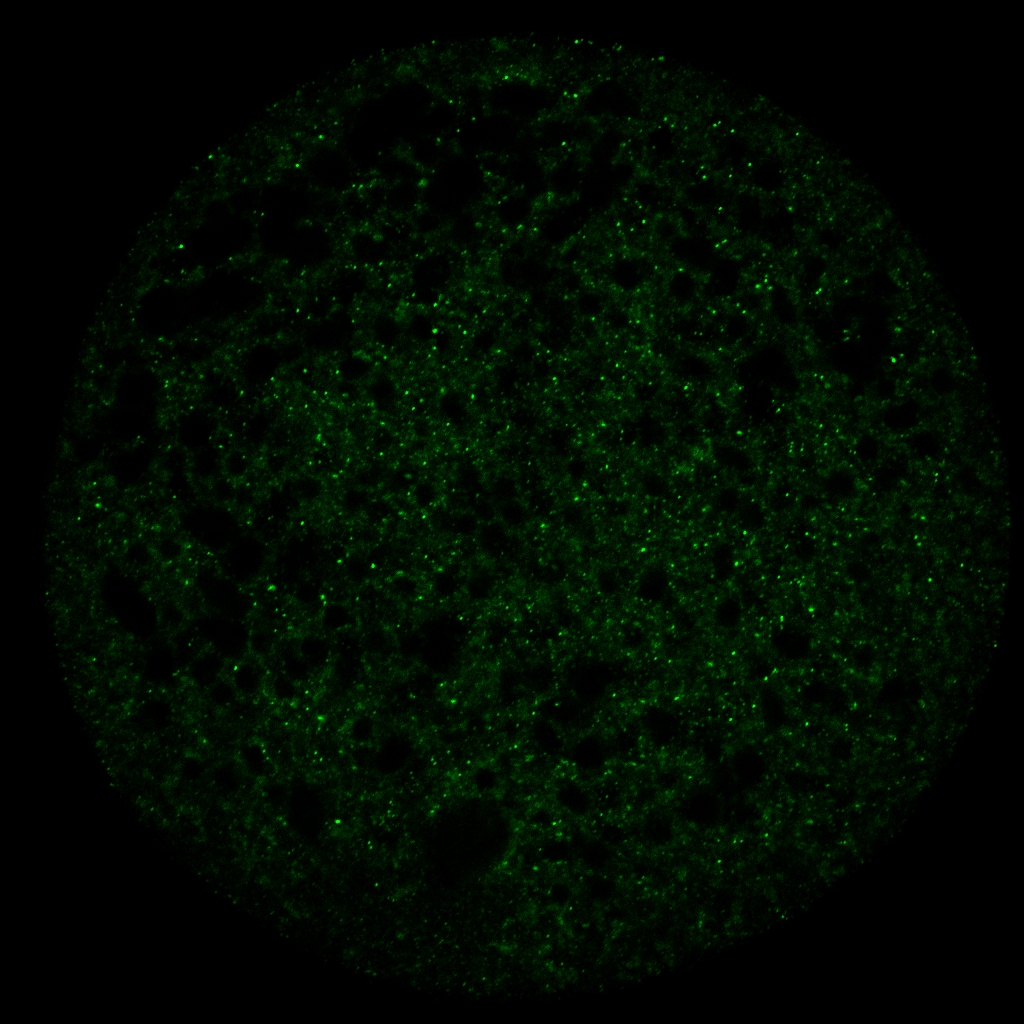

Supplement: S11 Raw images — (ZIP) [file pone.0277477.s013.zip › Fig6a_rot_c2.jpg]

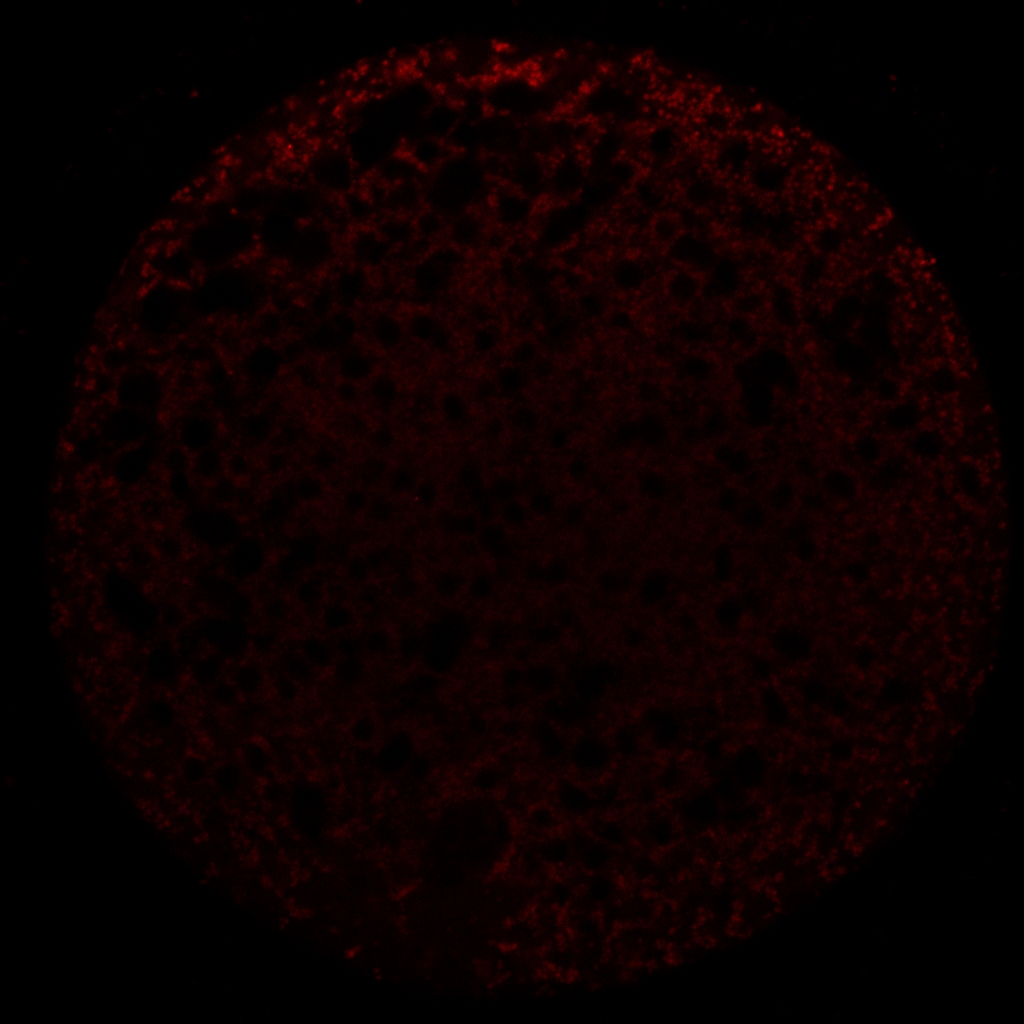

Supplement: S11 Raw images — (ZIP) [file pone.0277477.s013.zip › Fig6a_rot_c3.jpg]

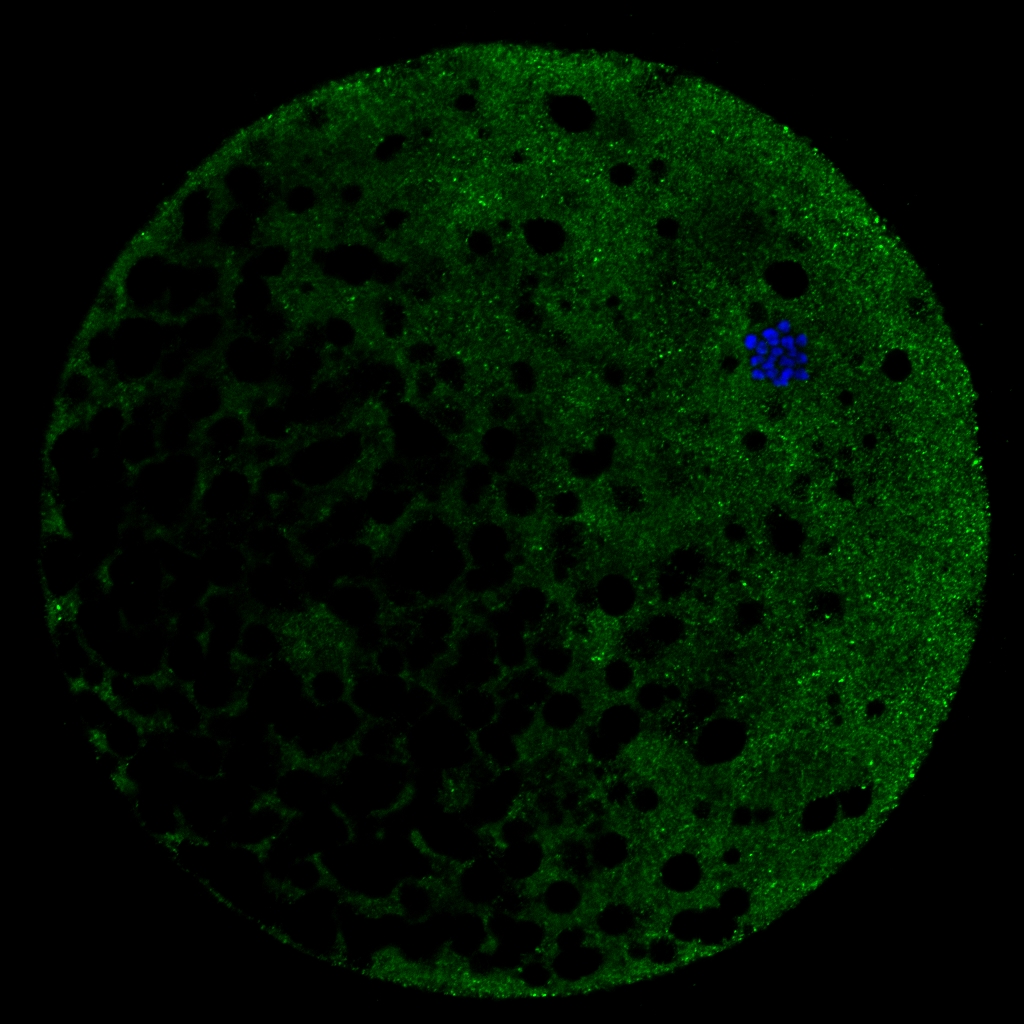

Supplement: S12 Raw images — (ZIP) [file pone.0277477.s014.zip › Fig6d_con_c1+2.jpg]

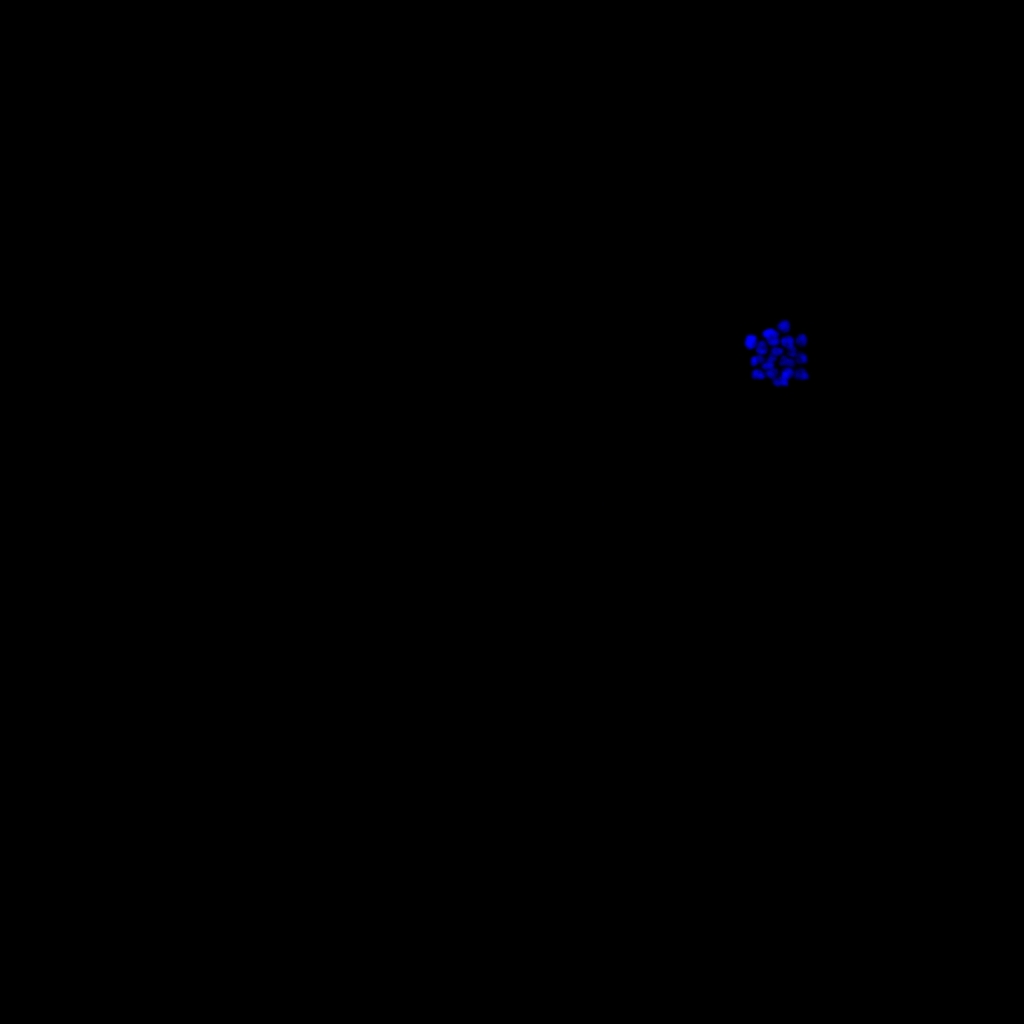

Supplement: S12 Raw images — (ZIP) [file pone.0277477.s014.zip › Fig6d_con_c1.jpg]

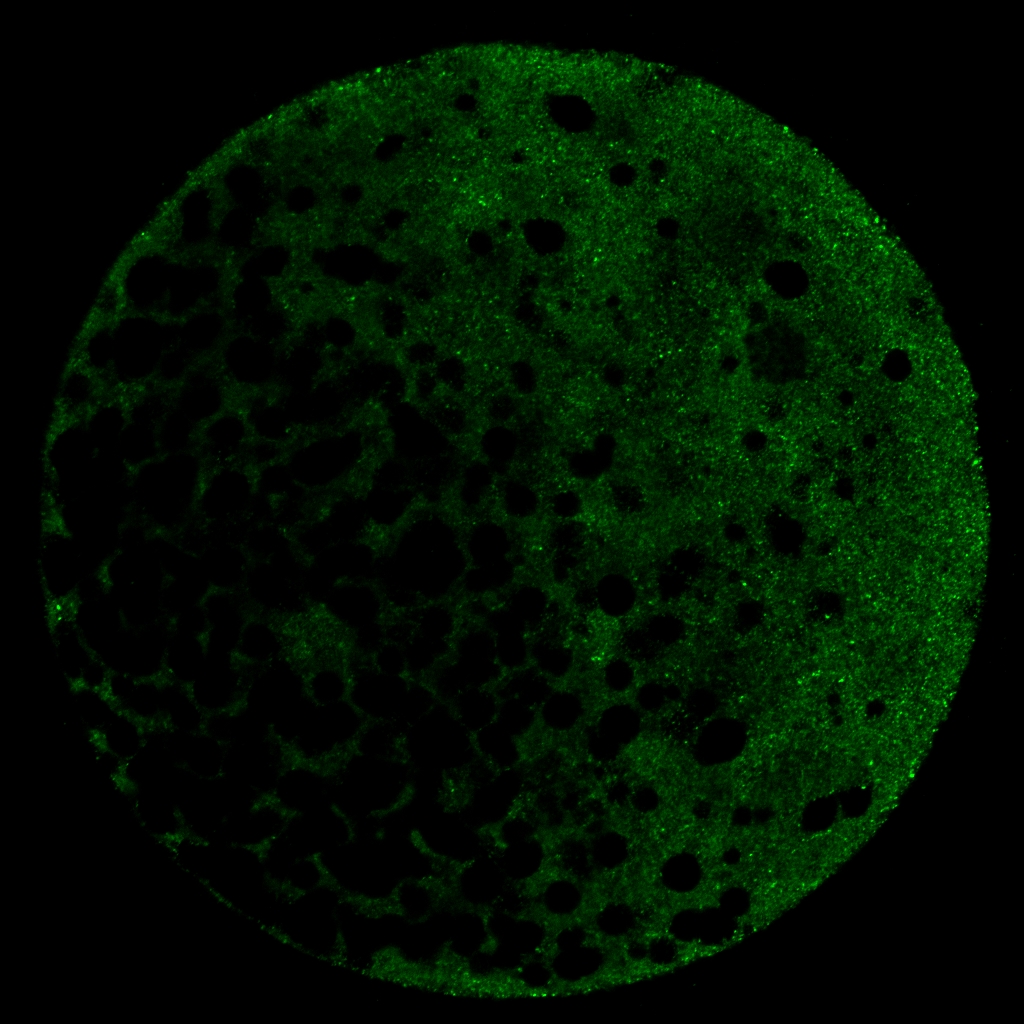

Supplement: S12 Raw images — (ZIP) [file pone.0277477.s014.zip › Fig6d_con_c2.jpg]

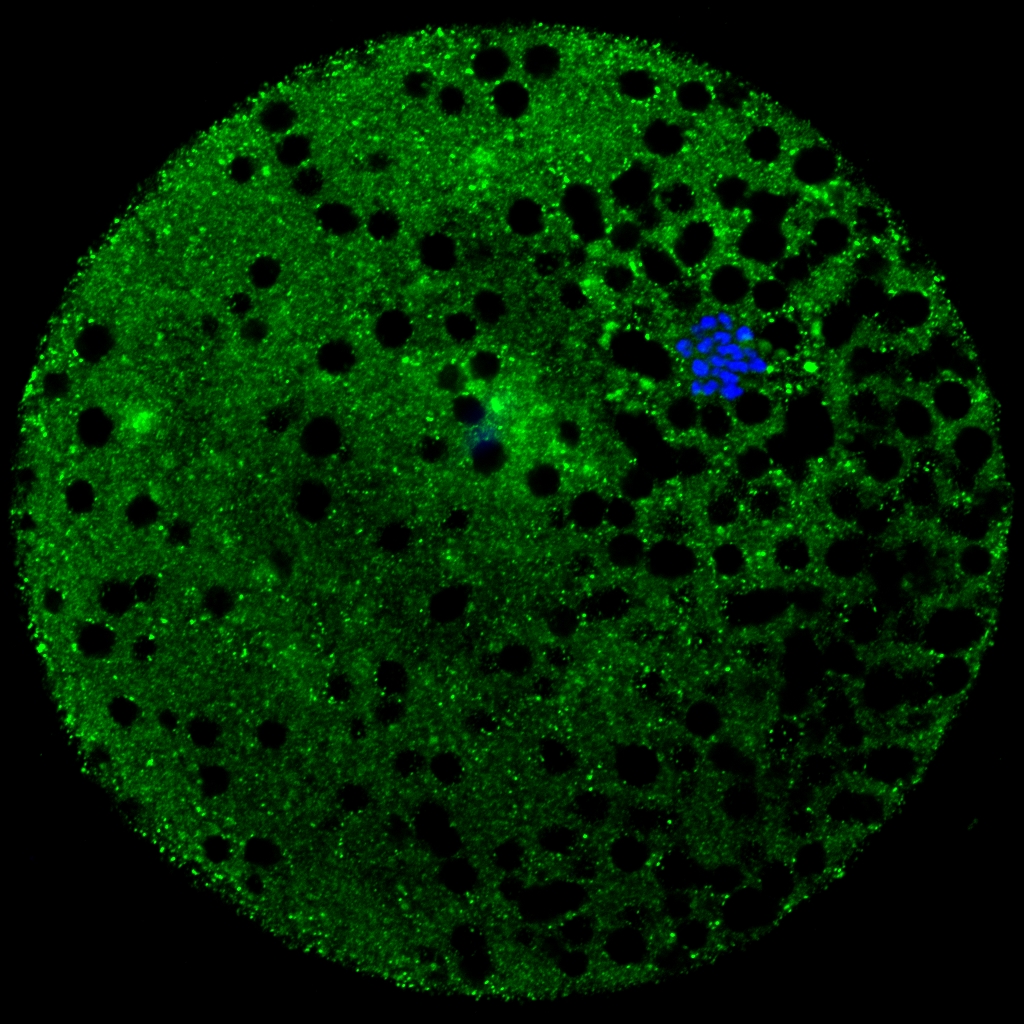

Supplement: S12 Raw images — (ZIP) [file pone.0277477.s014.zip › Fig6d_rot_c1+2.jpg]

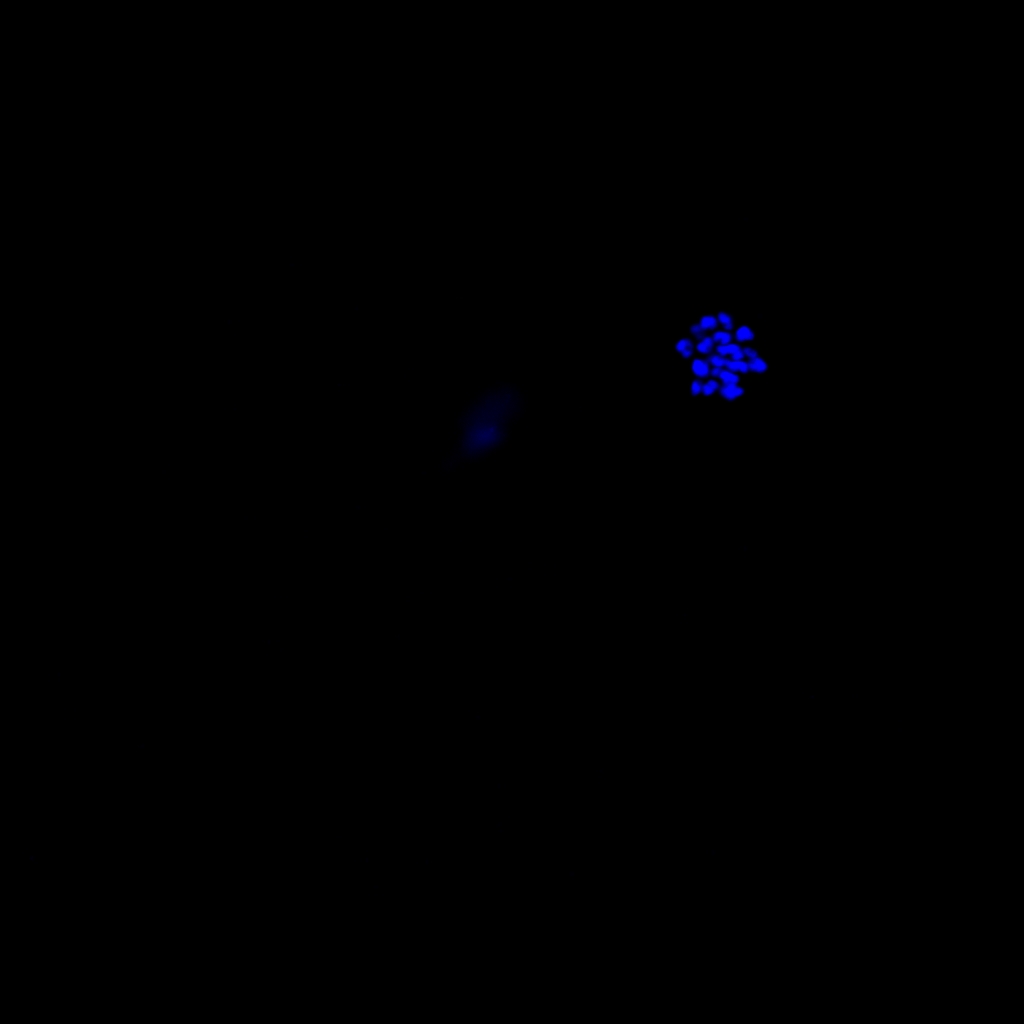

Supplement: S12 Raw images — (ZIP) [file pone.0277477.s014.zip › Fig6d_rot_c1.jpg]

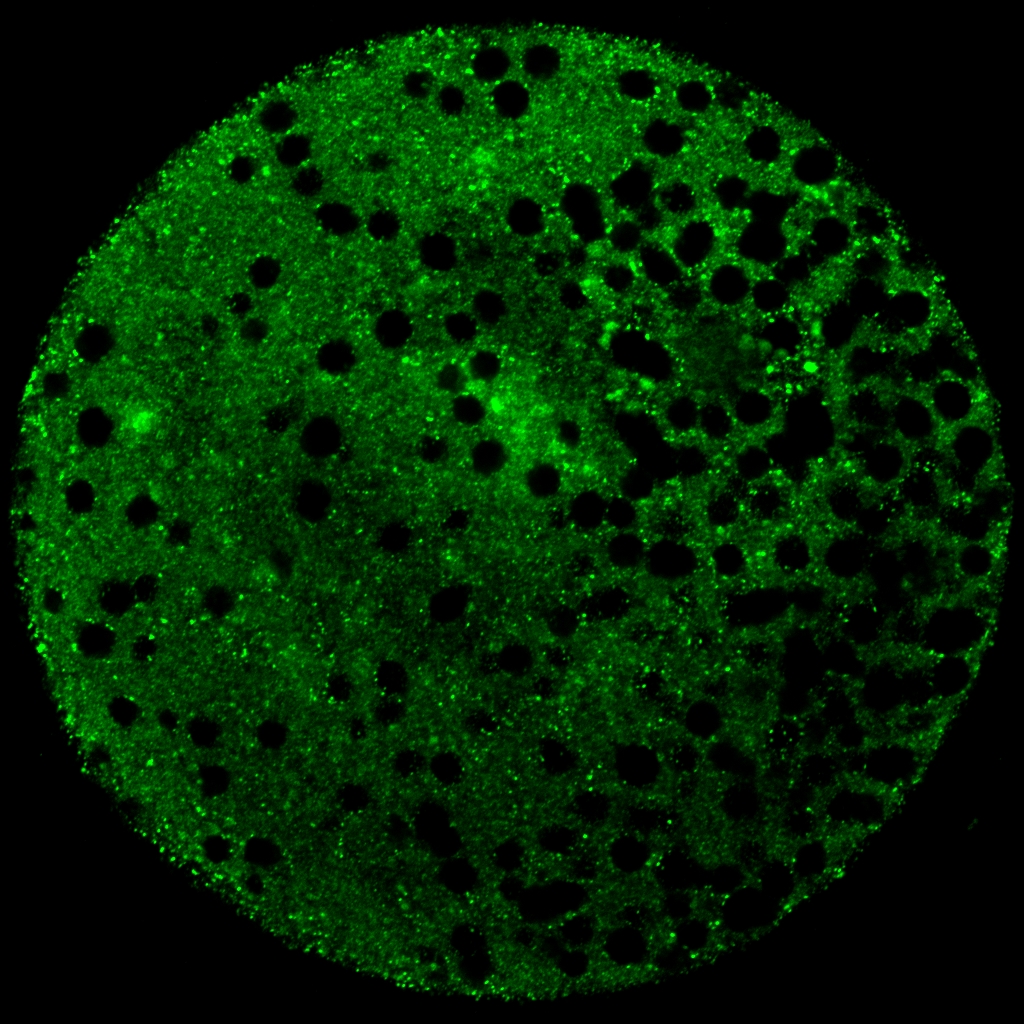

Supplement: S12 Raw images — (ZIP) [file pone.0277477.s014.zip › Fig6d_rot_c2.jpg]
