# Supplementary material for: Rotenone causes mitochondrial dysfunction and prevents maturation in porcine oocytes
Source: PLoS One. 2022 Nov 28;17(11):e0277477. doi: 10.1371/journal.pone.0277477 (PMC9704683; doi:10.1371/journal.pone.0277477)
Supplement: S1 Raw images — (PDF) [file pone.0277477.s003.pdf]

Rotenone Disrupts Meiotic Maturation via Inhibition of Mitochondrial Function in Porcine Oocytes

Original picture of western blot data

Figure 4F SIRT 1—120kDa

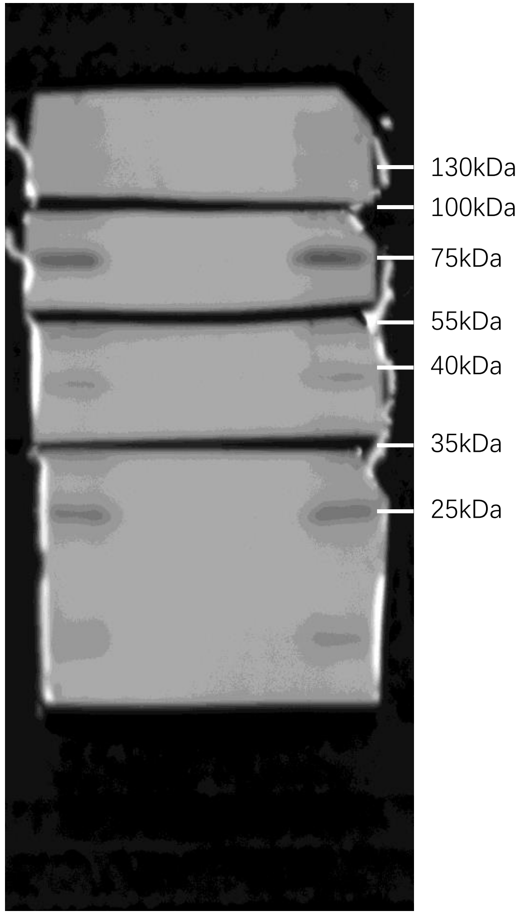

Protein marker

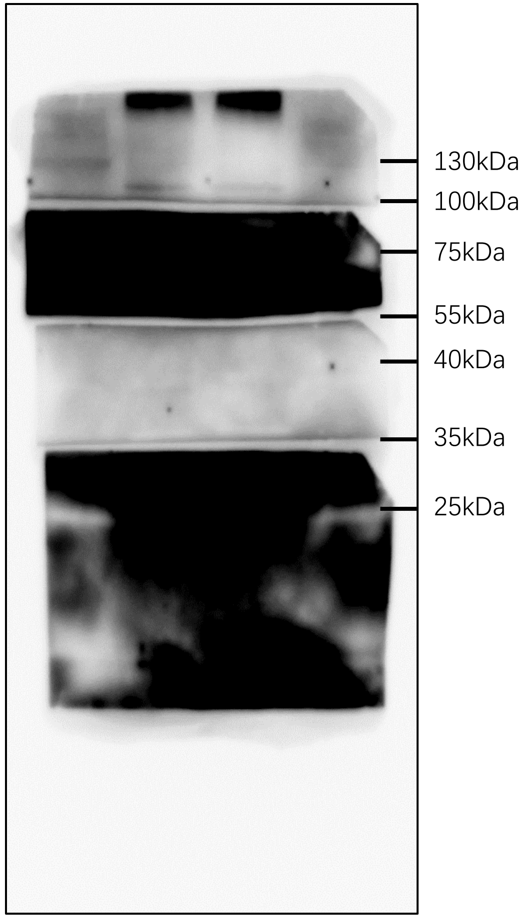

Antibody

Figure 4F GAPDH—36kDa

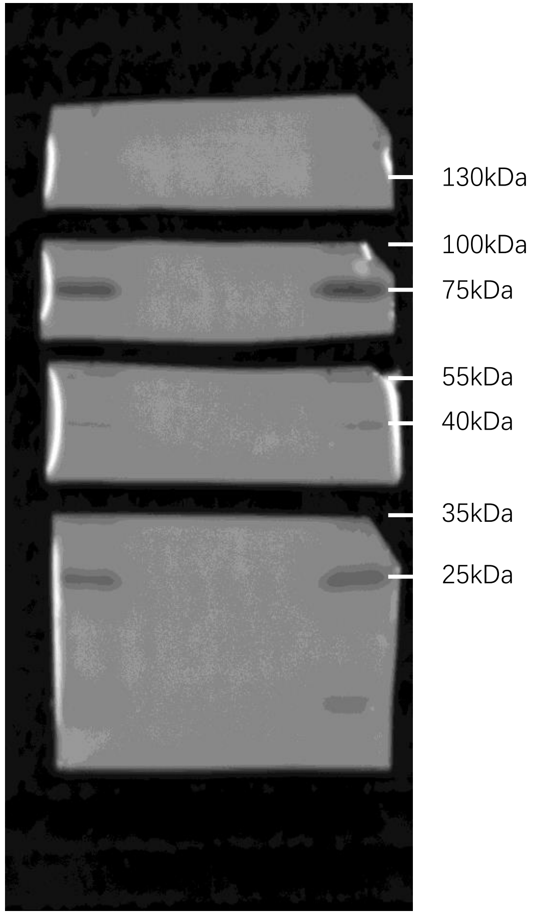

Protein marker

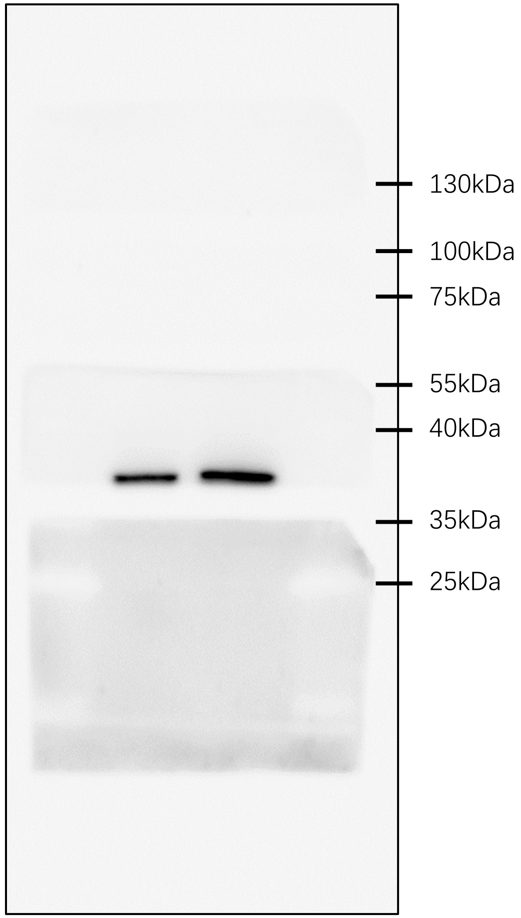

Antibody

- After stripping, re-blocking, cut membrane and GAPDH antibody incubation

Figure 5I   Ubiquitin—9kDa

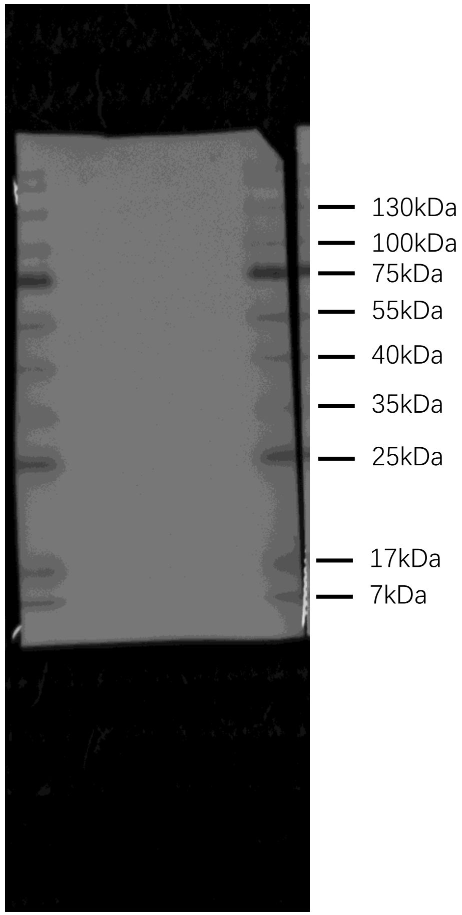

Protein marker

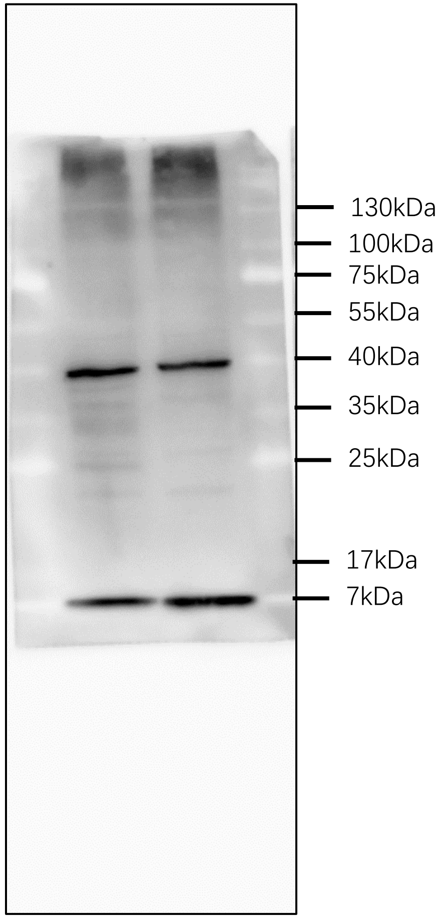

Antibody

Figure 5I GAPDH—36kDa

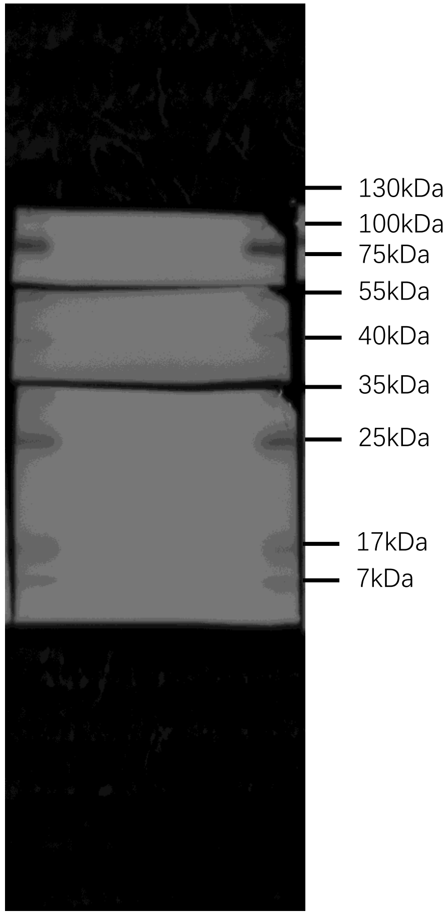

Protein marker

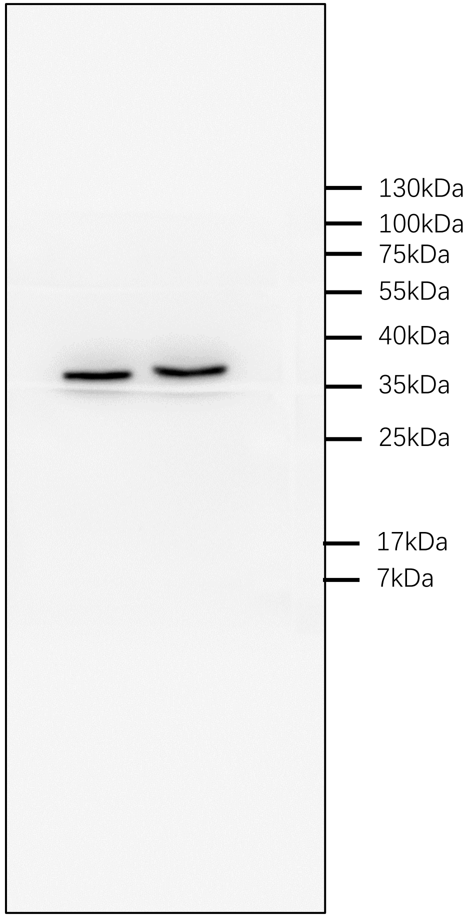

Antibody

- After ubiquitin, stripping, re-blocking, cut membrane and GAPDH antibody incubation
